# Phenolic Profile, Antioxidant Activity, and Wound-Healing Potential of *Oenothera rosea* and *Taxodium mucronatum* Extracts

**DOI:** 10.3390/plants15213391

**Published:** 2026-11-02

**Authors:** Lizett V. Bonifaz Delgado, Marilena Antunes Ricardo, Alberto Valdés, Daniel Guajardo Flores

**Affiliations:** 1 School of Engineering and Science, Tecnologico de Monterrey, Av. Eugenio Garza Sada 2501 Sur, Monterrey 64849, Nuevo León, Mexicomarilena.antunes@tec.mx (M.A.R.); 2 Institute for Obesity Research, Tecnologico de Monterrey, Av. Eugenio Garza Sada 2501 Sur, Monterrey 64849, Nuevo León, Mexico; 3 Laboratory of Foodomics, Institute of Food Science Research (CIAL), Spanish National Research Council (CSIC), Nicolas Cabrera 9, Cantoblanco Campus, 28049 Madrid, Spain; a.valdes@csic.es

**Keywords:** ethnobotany, phytomedicine, bioactivity, metabolomics, medicinal plants, diabetes, ulcer treatment, antioxidants

## Abstract

*Oenothera rosea* and *Taxodium mucronatum* are traditionally used in the Americas for wound healing; however, their bioactive properties remain insufficiently standardized and scientifically validated. Therefore, this study aimed to establish extraction conditions that maximize the antioxidant potential of *O. rosea* and *T. mucronatum* extracts, characterize their phenolic profiles, and evaluate their biological effects in vitro. Extraction temperature, solvent, and time were evaluated using a full factorial design, with total phenolic content and radical scavenging capacity (DPPH and ABTS) as response variables. Extracts’ phenolic profiles were characterized by UHPLC-PDA-Orbitrap-MS/MS. Nitric oxide production was assessed in RAW 264.7 macrophages by quantifying nitrite using the Griess assay. Fibroblast migration was evaluated under standard- and high-glucose conditions as an in vitro wound-healing model. Extracts obtained with 70% ethanol at 60 °C for 24 h exhibited the highest overall antioxidant activity. *O. rosea* was rich in phenolic acids and flavonoids, whereas *T. mucronatum* predominantly contained bioflavonoids and diterpenes. *T. mucronatum* significantly reduced nitric oxide production, whereas *O. rosea* showed no significant difference from the control. Under standard-glucose conditions, both extracts significantly enhanced fibroblast migration compared with the negative control. However, under high-glucose conditions, only the 50:50 mixture induced partial wound closure. These findings support the potential of both species as sources of bioactive compounds for wound management.

## 1. Introduction

Wound healing is a complex biological process that involves four overlapping phases: hemostasis, inflammation, proliferation, and tissue remodeling. Progression through these stages depends on a regulated balance between cellular signaling, inflammatory regulation, and redox homeostasis. Excessive production of reactive oxygen species (ROS) and prolonged inflammation can disrupt the process, resulting in delayed wound closure and the development of chronic wounds. These complications are particularly common in individuals with metabolic disorders such as diabetes mellitus, where elevated glucose levels promote oxidative stress which further affects cellular migration, angiogenesis, and extracellular matrix deposition [1].

Medicinal plants and their derivatives have historically been used for the treatment of a wide range of ailments, including wound management. Among their bioactive constituents, phenolic compounds have attracted particular interest due to their antioxidant and anti-inflammatory properties. These metabolites can scavenge reactive oxygen species (ROS) and modulate signaling pathways involved in inflammation and tissue repair, including those associated with nitric oxide regulation [2]. As oxidative stress and inflammatory imbalance are major contributors to impaired healing, plant-derived polyphenols have emerged as promising candidates for promoting tissue regeneration. Consequently, the identification and characterization of bioactive phytochemicals capable of supporting wound healing remains an active area of research.

*Oenothera rosea* L’Hér. ex Ait (syn. *Hartmannia rosea*), commonly known as pink evening primrose or hierba del golpe, and *Taxodium mucronatum* Ten. (Cupressaceae), known as Montezuma cypress or ahuehuete, are acknowledged as medicinal plants with healing effects according to the Mexican United States Pharmacopeia [3]. *O. rosea* is traditionally employed for the treatment of bruises and wounds [4,5,6]. It is an American native wild plant that comes from the *Onagraceae* family. It is noted for its anti-inflammatory [7,8], antinociceptive [9], and antibacterial properties [10,11]. A previous in-vivo study [12] demonstrated the wound-healing efficacy of *O. rosea* extract applied to incised wounds in rats, showing significantly improved healing compared to controls; however, its reliance on toxic solvents like chloroform and petroleum ether warrants further investigation to validate the clinical applicability of extracts obtained using safer and accessible alternatives. On the other hand, *T. mucronatum* has a long history of traditional medicinal use, especially in Mexico. Extracts from its bark and leaves have demonstrated anti-inflammatory effects [13], as well as additional reported healing and antimicrobial benefits [14].

Despite their traditional use and reported biological activities, both species remain insufficiently characterized from a phytochemical perspective. Existing studies provide only a partial description of their secondary metabolites, and the extraction conditions required to maximize the recovery of bioactive compounds have not been systematically investigated. Because extraction parameters strongly influence both phytochemical composition and biological activity, optimization is essential for the development of standardized plant-derived preparations. Furthermore, recent advances in metabolomics and high-resolution mass spectrometry have enabled a more comprehensive characterization of complex plant extracts, facilitating the identification of metabolites potentially responsible for their biological effects.

Therefore, the aim of the present study was to identify extraction conditions that maximize the antioxidant potential of *O. rosea* and *T. mucronatum* within a practically relevant range of processing variables using a full factorial design. The selected extracts were subsequently characterized by UHPLC-PDA-Orbitrap-MS/MS and evaluated for antioxidant and anti-inflammatory activities, as well as in vitro fibroblast migration. As an exploratory study, the objective was to establish associations between phytochemical composition and biological activity, identify promising bioactive extracts, and generate hypotheses for future mechanistic investigations. By integrating extraction optimization, metabolomic characterization, and biological evaluation, this work contributes to the scientific validation of these traditionally used species and provides a foundation for their potential application in wound management.

## 2. Results

### 2.1. Total Yield

Extraction yields varied according to the conditions evaluated in the factorial design for both *O. rosea* and *T. mucronatum* (Table 1). For *O. rosea*, the yield was significantly affected (*p* < 0.05) by all three independent variables: solvent type, extraction time, and temperature. The average yield of extracts using water was 117.39 mg/g, compared to 66.53 mg/g for those using 70% ethanol. Extractions conducted for 1 h yielded an average of 75.81 mg/g, whereas those conducted for 24 h yielded an average of 108.12 mg/g. Regarding temperature, the average yields were 84.02 mg/g at 25 °C, 87.32 mg/g at 40 °C, and 104.55 mg/g at 60 °C. For *T. mucronatum*, only the solvent type and extraction time had significant effects (*p* < 0.05) on yield. The average yield for extracts using water was 71.19 mg/g, while those using 70% ethanol averaged 91.01 mg/g. Extractions lasting 1 h had an average yield of 75.75 mg/g, compared to 86.45 mg/g for those lasting 24 h. In terms of temperature, the average yields were 78.14 mg/g at 25 °C, 79.75 mg/g at 40 °C, and 85.42 mg/g at 60 °C; however, these differences were not statistically significant (*p* > 0.05). When analyzing individual runs, the sample 24, extracted for 24 h at 60 °C with 70% ethanol, had a yield of 117.3 mg/g, statistically higher (*p* < 0.05) than the rest of the samples.

These results indicate that the extraction conditions associated with maximum yield differed between species, with *O. rosea* favoring aqueous extraction and *T. mucronatum* favoring hydroethanolic extraction.

### 2.2. Total Phenolic Content and In Vitro Antioxidant Capacity

**Figure 1:**
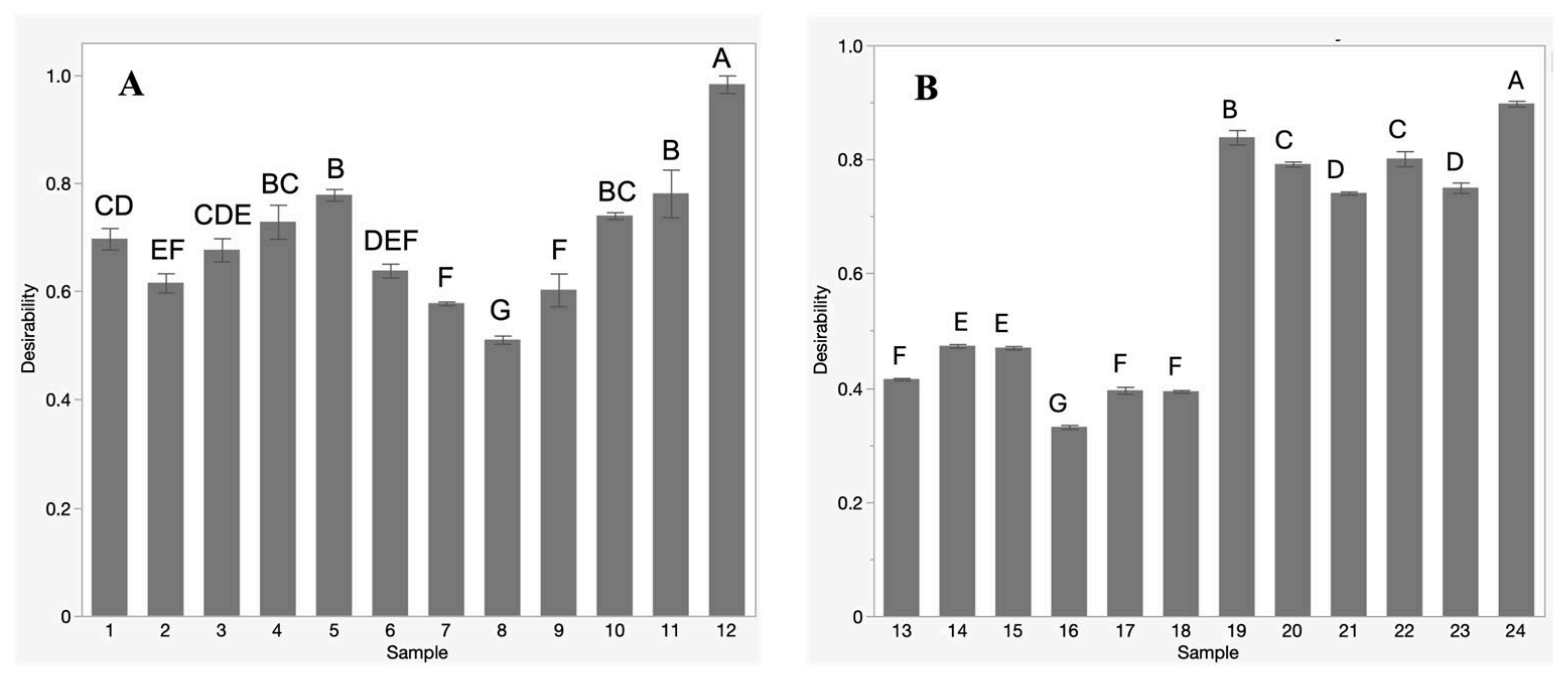
Overall desirability for all antioxidant ability assays calculated with arithmetic mean. (**A**) Results for *O. rosea*. (**B**) Results for *T. mucronatum*. Bars represent mean ± standard deviation of *n* = 3 independent experiments. Different letters indicate differences among treatment conditions using Tukey test (*p* < 0.05).

The individual antioxidant activity per assay of *O. rosea* extracts are reported in Table 2. It can be observed that TPC ranged from 90.9 to 220.2 mg GAE/g dry weight of extract. Notably, samples 12, 4, and 3 demonstrated significantly higher levels (*p* < 0.05), with TPC values of 220.2, 210.6, and 203.6 mg GAE/g extract, respectively. In the DPPH radical scavenging assay, antioxidant values varied between 45.6 and 99.0 μmol Trolox/g extract, with sample 12 having a significantly higher values (*p* < 0.05) in comparison to all other samples. For the ABTS scavenging assay, values ranged from 140.5 to 268.4 μmol Trolox/g extract, where samples 12 and 11 exhibited the highest antioxidant activities with 268.4 and 239.2 μmol Trolox/g extract, respectively. Previous researchers have reported similar results [15] evaluated the antioxidant capacity of *O. rosea*, resulting in a TPC of 135.68 mg GAE/g for the ethanolic extract and 26.78 mg GAE/g for the aqueous extract. The DPPH radical scavenging activity was 72.9% and 76.97% for the ethanolic and aqueous extracts, respectively. In another study, ref. [16] reported a TPC of 65.8 mg GAE/g, a DPPH value of 615.5 μmol TE/g, and an ABTS value of 438 μmol TE/g for a sample extracted under acidic conditions using methanol 80% as the solvent. The highest antioxidant activity for *O. rosea*, obtained by overall desirability (Figure 1A), was achieved using 70% ethanol at 60 °C for 24 h, corresponding to sample 12 (O100).

For *T. mucronatum*, the antioxidant activity of individual extracts obtained under each treatment is shown in Table 3. TPC varied from 70.3 to 189.6 mg GAE/g extract; sample 19 had significantly higher TPC (*p* < 0.05) compared to other samples (Table 3). For the DPPH radical scavenging assay, values ranged from 30.2 to 88.8 μmol Trolox/g extract, with samples 24, 22, and 20 showing the highest values of 88.8, 83.4, and 84.1 μmol Trolox/g extract, respectively. The ABTS assay results ranged from 132.7 to 316.8 μmol Trolox/g extract, with sample 24 demonstrating a statistically significantly higher value (*p* < 0.05) compared to the other samples. For *T. mucronatum*, there are no results published directly; however, the antioxidant activity of similar bald cypress *Taxodium distichum* aqueous extract resulted in a TPC of 70.99 mg GAE/g dw and DPPH radical scavenging of 13.1% [17]. The antioxidant activity of *T. mucronatum*, as determined by the overall desirability analysis (Figure 1B), was significantly lower (*p* < 0.05) in water extracts than in hydroethanolic extracts, suggesting a reduced recovery of antioxidant compounds. This difference may be related to the lower extraction efficiency of water for certain antioxidant constituents, as well as differences in solvent penetration and mass transfer within the plant matrix.

These findings indicate that the effect of solvents on antioxidant activity was species dependent. While hydroethanolic extraction consistently enhanced the antioxidant activity of *T. mucronatum*, the response of *O. rosea* was influenced by the interaction between solvent, temperature, and extraction time, with aqueous extracts exhibiting higher activity under some extraction conditions.

### 2.3. UHPLC-PDA-ESI-MS Analysis of Individual Phenolic Compounds

**Figure 2:**
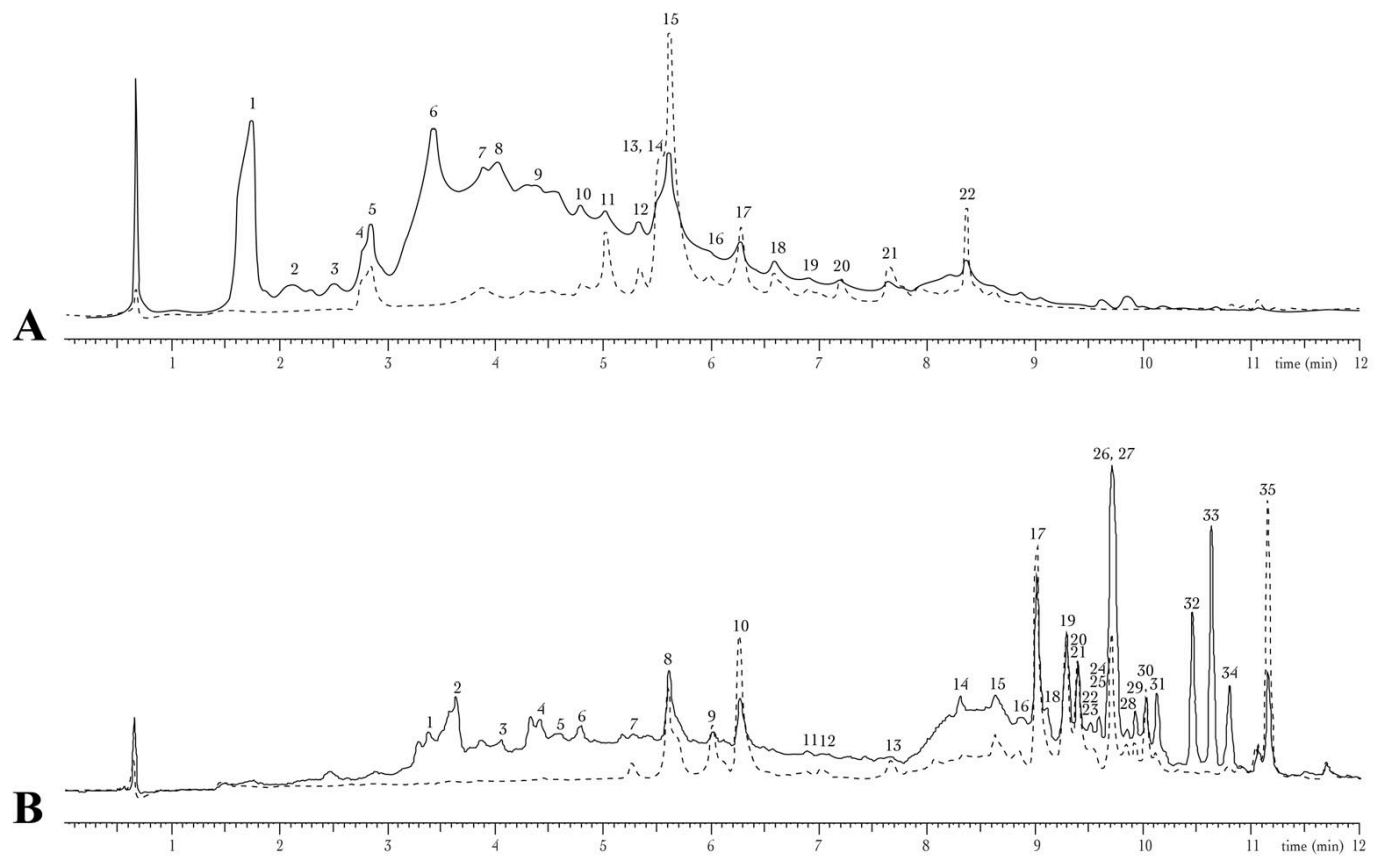
Chromatographic profile of plant extracts obtained by UHPLC-PDA. Continuous line represents absorbance at 280 nm; dotted line represents absorbance at 320 nm. (**A**) *Oenothera rosea*; (**B**) *Taxodium mucronatum*. Both extracts were obtained with 70% ethanol at 60 °C for 24 h.

*O. rosea* extract obtained at 60 °C for 24 h with 70% ethanol was analyzed, and the resulting chromatograms obtained at two different wavelengths (280 and 320 nm) are presented in Figure 2A. The relevant features were tentatively annotated using NIST and MoNA databases, complemented by SIRIUS for the elucidation of molecular formulas and compound families of unknowns, yielding varying levels of identification. This multi-tool approach resulted in different identification levels, as summarized in Table 4. Peaks 1, 6 and 15 are the most prominent ones, tentatively identified as gallic acid, a coumaric acid derivative, and a quercetin-3-*O*-hexoside, respectively. Additional significant peaks were noted at retention times ranging from 3 to 7 min, exhibiting a diverse and chemically rich profile of secondary metabolites, particularly phenolic compounds and flavonoids. Among the compounds tentatively identified are hydroxybenzoic acids (e.g., protocatechuic acid, ethyl gallate), hydroxycinnamic acids (e.g., caftaric acid, neochlorogenic acid, 4-*O*-*p*-coumaroylquinic acid), various quercetin derivatives, and other complex flavonoid glycosides such as delphinidin 3-*O*-rutinoside, afzelin, and other flavonoid-3-*O*-glycosides. The phytochemical profile of *O. rosea* showed both agreement with and expansion of previous reports. Compounds such as gallic acid, *p*-coumaric acid, and quercetin derivatives have been previously described in the species [18,19,20,21]. However, the present analysis revealed a broader diversity of hydroxybenzoic acids, hydroxycinnamic acids, and flavonoid glycosides. Similarly, Calva-Candelaria et al. reported numerous previously uncharacterized phenolic acids, flavonoids, and related metabolites using mass spectrometry [22]. In contrast, compounds previously reported for *O. rosea*, including neochlorogenic acid, delphinidin, and cyanidin [21], were not detected in the present study.

Conversely, Table 5 presents the compounds tentatively identified in *T. mucronatum* extract obtained at 60 °C for 24 h with 70% ethanol, with the corresponding UV chromatograms shown in Figure 2B. Untargeted UHPLC-MS/MS profiling revealed a diverse phytochemical composition, with features tentatively annotated as flavan-3-ols, flavonol glycosides, biflavonoids, and diterpenoids based on accurate mass measurements, MS/MS fragmentation patterns, and comparison with available spectral databases and reference libraries. Among the flavan-3-ols, catechin, epicatechin, and procyanidins were identified, while the flavonol fraction comprised quercetin-3-*O*-vicianoside, quercetin-3-*O*-hexoside, reynoutrin, quercitrin, kaempferol-3-*O*-rhamnoside, and the aglycone quercetin. The biflavonoid profile included both C–C and C–O–C linked structures such as cupressuflavone, hinokiflavone, 4′-*O*-methylated flavonoids, and isoginkgetin. Diterpenoids identified were primarily of the abietane-type, as well as labdane-related compounds such as pisiferal derivatives. The sample composition further includes distinctive metabolites such as naringenin. Several diterpenes and their dimers remain uncharacterized and are not currently represented in LC-MS libraries; however, their fragmentation patterns are now being investigated and suggest that most of these compounds are dehydroabietic acid derivatives [23]. The phytochemical profile of *T. mucronatum* was consistent with previous reports describing the presence of phenolic acids, flavonoids, biflavonoids, and diterpenoids [24]. Several detected features showed *m*/*z* values and fragmentation patterns consistent with biflavonoids previously reported in the species and related taxa, including podocarpusflavone A, sciadoptysin, bi-apigenins, cryptomerins, and amentoflavone derivatives [25,26,27]. Likewise, *m*/*z* values corresponding to diterpenoids such as ferruginol, sugiol, taxodione, salvinolone, and royleanone were observed in past studies [28]. While some previously reported metabolites were not detected, compounds such as quercetin and naringenin were confirmed in aglycone or glycosylated forms [29]. Therefore, the results support earlier phytochemical findings while expanding the evidence for the chemical diversity of *T. mucronatum*.

### 2.4. Cellular Antioxidant Activity

**Figure 3:**
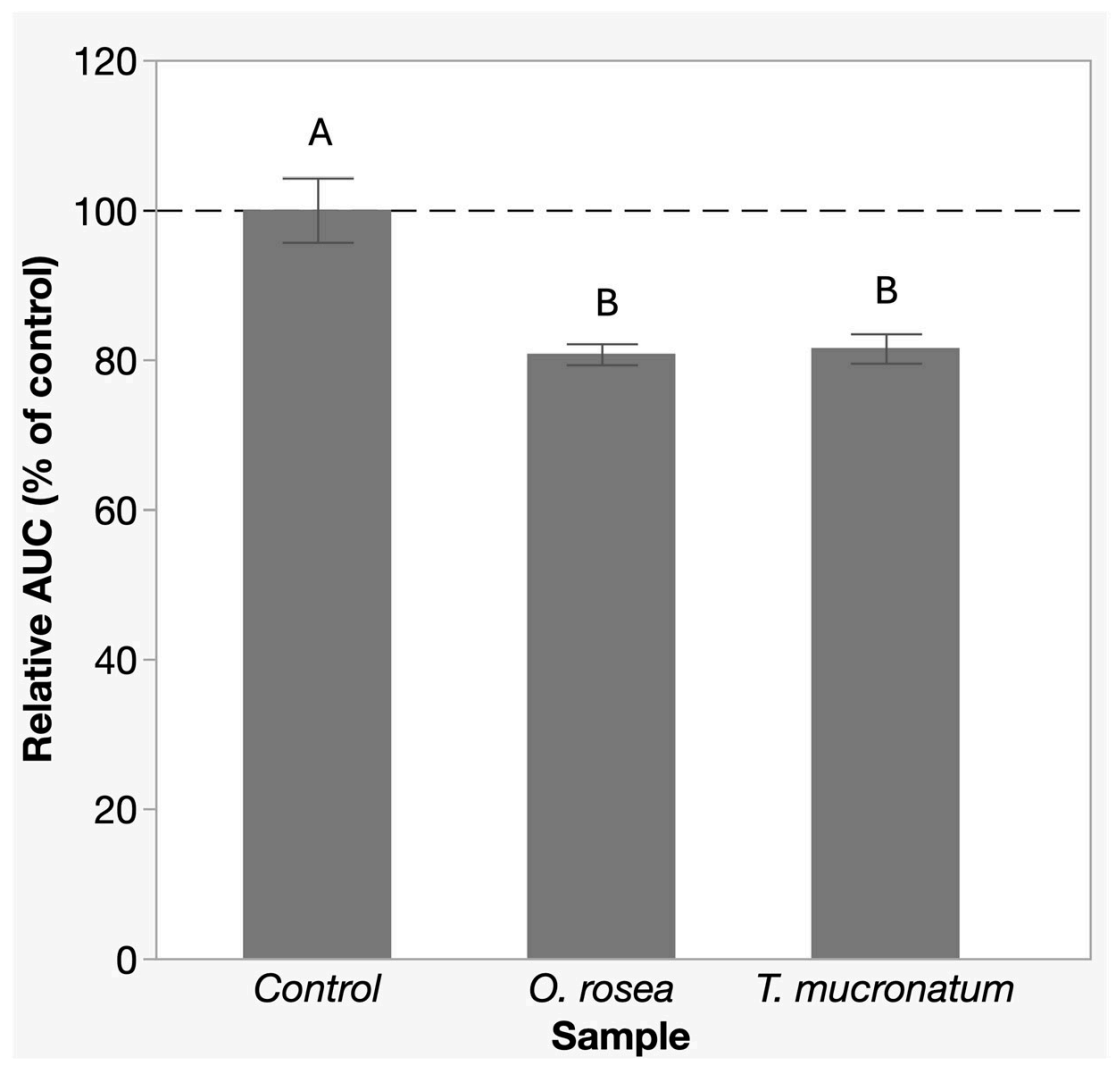
Cellular antioxidant activity expressed as relative area under the curve (AUC) over 90 min. Human dermal fibroblasts were loaded with DCFH-DA and stimulated with AAPH to induce oxidative stress. Treated samples were exposed to *O. rosea* and *T. mucronatum* extracts obtained at 60 °C for 24 h with 70% ethanol at a concentration of 50 µg/mL; the control sample was untreated. AUC values for each sample were normalized to the control (set as 100% oxidation). Bars represent mean ± standard deviation of technical triplicates. Letters above the bars indicate significant differences among treatments according to Tukey’s test at α = 0.05.

Both *O. rosea* and *T. mucronatum* extracts significantly reduced AAPH-induced oxidative stress in fibroblasts at a concentration of 50 µg/mL, showing inhibition values of 16.8% and 16.2%, respectively, relative to the AAPH-stimulated control cells. While both extracts exhibited significant antioxidant activity compared with the stimulated control, no statistically significant differences were observed between the two extract treatments (*p* > 0.05) (Figure 3). Despite their different phytochemical compositions, both extracts displayed comparable cellular antioxidant activity under the conditions evaluated. These findings suggest that distinct phytochemical profiles may result in similar overall antioxidant effects in this cellular model.

### 2.5. Nitric Oxide Quantification Assay

**Figure 4:**
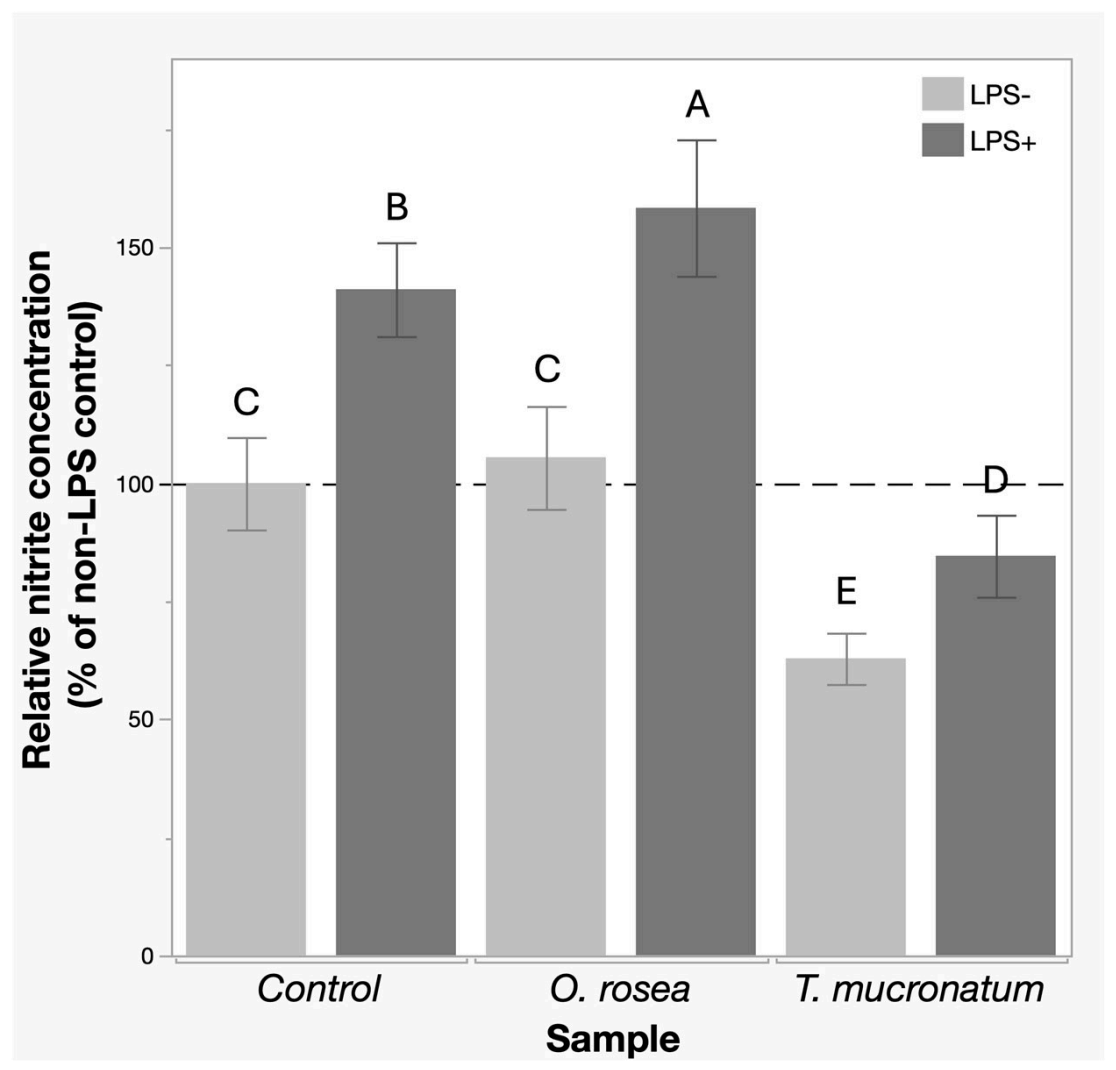
Nitric oxide production measured using the Griess colorimetric assay in RAW 264.7 cells treated with extracts. Values were normalized to non-LPS-stimulated control. Light grey bars represent unstimulated cells, and dark grey bars represent LPS-stimulated cells. Both *O. rosea* and *T. mucronatum* extracts were obtained with 70% ethanol at 60 °C for 24 h and added at a concentration of 20 µg/mL. Control = untreated cells. Bars represent mean ± standard deviation of technical triplicates. Letters above the bars indicate significant differences among treatments according to Tukey’s test at α = 0.05.

Nitrite production was measured as an indirect marker of NO synthesis, which plays a crucial role in the inflammatory phase of wound healing. As depicted in Figure 4, treatment with *T. mucronatum* extract resulted in a significant reduction in nitrite production (*p* < 0.05) in both LPS-stimulated and non-stimulated cells, indicating a pronounced inhibitory effect on macrophage-mediated NO synthesis, which suggests a strong anti-inflammatory activity. In contrast, when cells were treated with *O. rosea* extract, no reduction in NO synthesis was observed.

### 2.6. Fibroblast Migration In Vitro Assay

**Figure 5:**
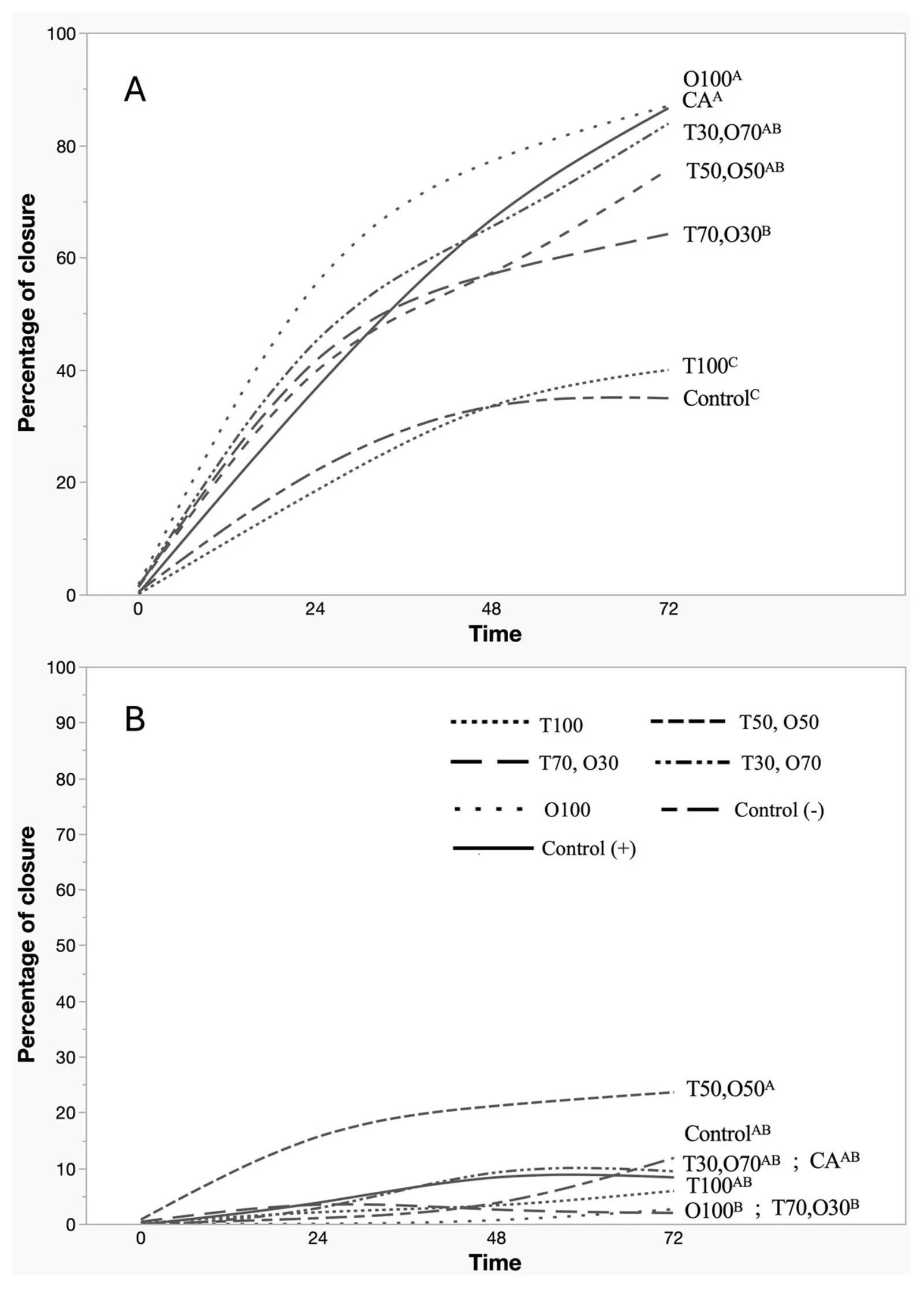
In vitro fibroblast migration assay in dermal fibroblasts treated with *O. rosea* and *T. mucronatum* extract mixtures, expressed as percentage of closure over 72 h. (**A**) Closure in cells cultured with standard medium (1 g/L glucose); (**B**) closure in cells cultured with high-glucose medium (5 g/L glucose). Sample codes indicate extract proportions: O = *O. rosea*, T = *T. mucronatum*; numbers denote percentage composition (e.g., O100 = 100% *O. rosea*, T50,O50 = 50% *T. mucronatum*, 50% *O. rosea*). CA = *Centella asiatica*; Control = untreated cells. Superscript letters indicate significant differences among treatments at 72 h (Tukey’s test, α = 0.05).

**Figure 6:**
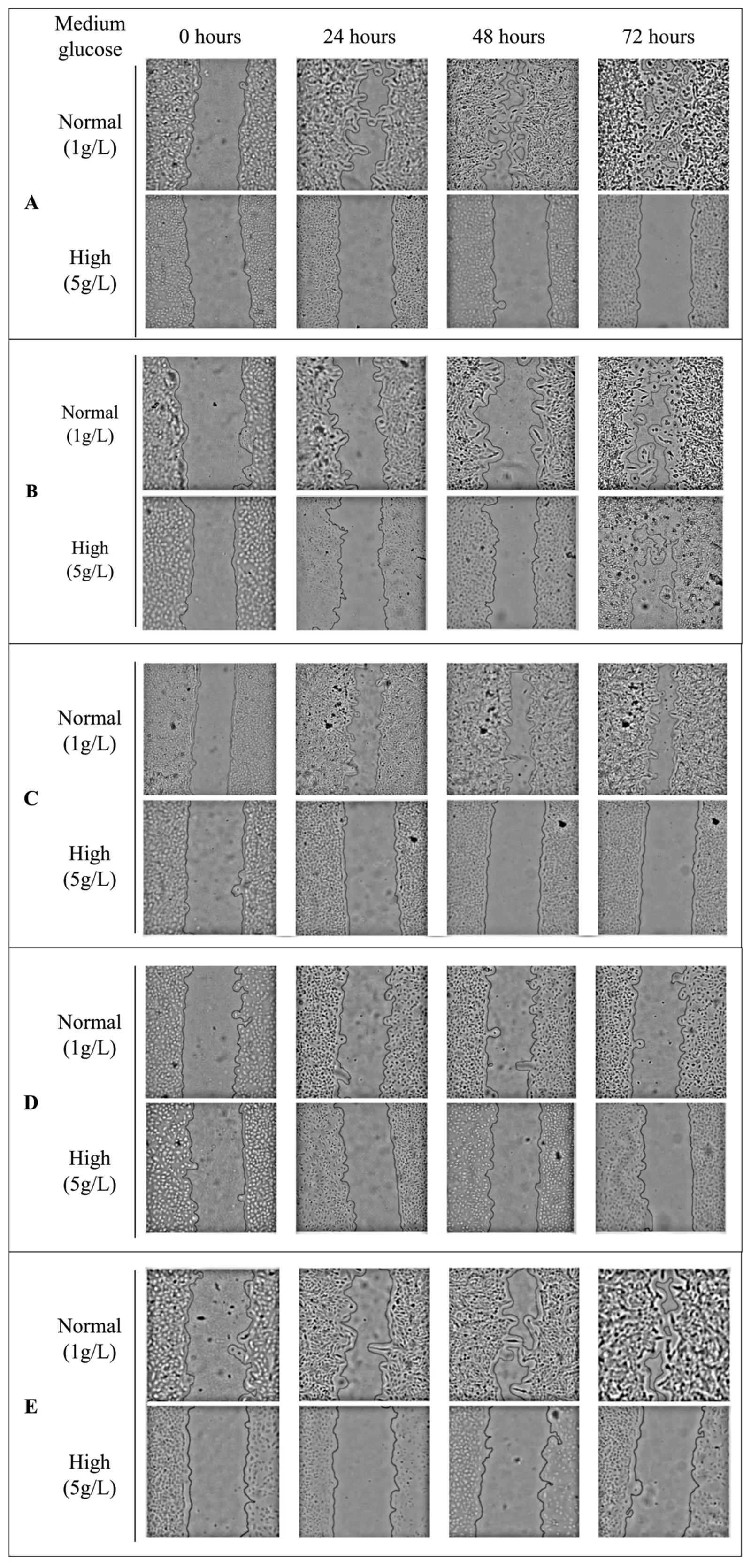
Representative microscopic images of human skin fibroblasts during the in vitro fibroblast migration assay. Each row illustrates closure progression at 0, 24, 48, and 72 h. Treatments: (**A**) *O. rosea* extract; (**B**) 50% *T. mucronatum* + 50% *O. rosea* extract; (**C**) *T. mucronatum* extract; (**D**) untreated control; (**E**) *Centella asiatica* extract. All treatments were tested at a final concentration of 20 µg/mL under both standard-glucose (1 g/L) and high-glucose (5 g/L) conditions.

The fibroblast migration assay revealed the beneficial effects of *O. rosea* under standard glucose conditions. As can be observed in Figure 5A, there was a noticeable increase in the percentage of closure, corresponding to higher concentrations of *O. rosea* extract. The sample O100, consisting of pure *O. rosea* extract, reached approximately 90% closure at 72 h, superior to the positive control (*Centella asiatica*), which achieved 80% closure. In contrast, the addition of *T. mucronatum* extract limited wound closure, with T100 achieving 43%, which is lower than O100, but higher than the 28% obtained with the untreated control sample. The cell migration in standard glucose conditions can be observed in the top panels of Figure 6A–E.

Under hyperglycemic conditions, which simulate diabetic wound environments, wound closure was markedly impaired across all groups. As seen in Figure 6B, most samples exhibited minimal closure (5–10%), with the notable exception of the T50,O50 sample, which maintained a moderate closure rate of 30%. Morphological analysis (shown in the bottom panels of Figure 6A–E) further supports these findings; cells under high glucose displayed a rounded morphology, deviating from the elongated phenotype typical of migrating fibroblasts, which is indicative of reduced motility.

## 3. Discussion

The contrasting responses observed among extraction treatments highlight the importance of considering both extraction yield and extract composition when evaluating the recovery of bioactive compounds. The higher yields obtained for *O. rosea* under aqueous extraction indicate the presence of polar compounds, which are readily solubilized in water [30]. However, the superior antioxidant activity observed under hydroethanolic conditions at higher temperature and longer extraction times suggests that compounds contributing most strongly to antioxidant capacity are more efficiently recovered or better preserved in the presence of an organic solvent. In contrast, the consistently greater antioxidant activity of *T. mucronatum* hydroethanolic extracts indicates that its major antioxidant constituents are likely less polar and more dependent on organic solvents for efficient extraction. Hydroethanolic systems are known to improve cell wall permeability and facilitate the recovery of phenolics across a broader polarity range. Furthermore, increasing temperature and extraction time promotes the release of cell wall-bound phenolics, but may also accelerate hydrolytic and oxidative degradation, particularly in aqueous systems [31,32]. Therefore, samples 12 and 24, which exhibited the highest overall antioxidant activity and corresponded to *O. rosea* and *T. mucronatum* extracts obtained with 70% ethanol at 60 °C for 24 h, were selected for subsequent characterization and biological evaluation. Hereafter, these extracts are designated O100 and T100, respectively.

Despite their contrasting phytochemical profiles, both O100 and T100 exhibited significant cellular antioxidant activity relative to the control. This finding indicates that both extracts contain constituents capable of protecting cells against oxidative stress, although their chemical composition differed markedly. *O. rosea* was characterized primarily by phenolic acids and flavonoid glycosides, whereas *T. mucronatum* contained additional biflavonoids and diterpenoids. Notably, both extracts contained quercetin-derived glycosides, which may partly explain their comparable antioxidant effects given the well-established cellular antioxidant activity of these flavonoids [33]. Multiple hydroxyl groups in these structures facilitate electron donation and free-radical scavenging, while glycosylation generally enhances aqueous solubility. Nevertheless, the lack of metabolite quantification precludes assigning the observed activity to specific compounds. Moreover, the additional diterpenoids present in T100 may have contributed less to the measured cellular antioxidant response, as their relatively hydrophobic nature could limit solubility and cellular availability under in vitro conditions, a phenomenon previously reported for terpenoid-rich extracts in cell permeability models [34]. These interpretations remain speculative and should be confirmed through future studies involving metabolite quantification, uptake analyses, and testing of isolated compounds.

In that regard, the evaluation of nitric oxide production revealed important functional differences between the extracts. T100 significantly reduced nitrite accumulation in LPS-stimulated macrophages, whereas O100 showed no significant inhibition. Previous studies have shown that flavones and biflavones possess stronger NO inhibitory activity than many other phenolic subclasses, particularly when hydroxyl and methoxyl substitutions are present at positions associated with enhanced anti-inflammatory activity [35,36,37]. Such inhibition of NO production has been linked to radical scavenging activity, suppression of iNOS expression, and interference with upstream signaling pathways such as NF-κB [38,39]. Consistent with these observations, T100 was enriched in biflavones including cupressuflavone, hinokiflavone, and 4′-*O*-methylated flavonoids. Compounds structurally related to these metabolites have been reported to suppress NF-κB activation and inhibit the expression of both iNOS and COX in activated macrophages [40]. Therefore, the reduced NO production observed in T100-treated cells is likely associated with the abundance of biflavonoid structures capable of modulating inflammatory signaling.

In contrast, O100 lacked flavones and was predominantly composed of flavonoid glycosides. Glycosylation has been reported to reduce NO inhibitory activity relative to the corresponding aglycones, largely because glycosyl groups increase hydrophilicity and sterically hinder interactions with cellular targets [36,41]. These structural characteristics may explain the limited effect of O100 on NO production despite its pronounced antioxidant activity. Together, these findings suggest that antioxidant activity and NO modulation are governed by different phytochemical features, with the latter being more dependent on the specific structural characteristics of individual phenolic compounds.

The wound-healing results further support this interpretation. Under normoglycemic conditions, both extracts promoted fibroblast migration compared with the negative control, indicating that antioxidant protection contributes positively to the repair process. However, O100 produced a greater wound closure response than T100, coinciding with the maintenance of NO production. Nitric oxide is known to play a central role in wound healing by regulating fibroblast activation, collagen synthesis, angiogenesis, and the expression of cytokines and growth factors involved in tissue repair [42,43]. Furthermore, NO contributes to the regulation of inflammatory cell recruitment and mediators such as IL-8 and TGF-β1 [44,45]. These observations suggest that the combination of antioxidant protection and sustained NO signaling may generate a more favorable environment for fibroblast migration than antioxidant activity alone.

The relationship between oxidative stress and NO availability may be particularly relevant to explain these results. Although NO promotes tissue repair, its biological activity is highly dependent on the surrounding redox environment [46]. Excessive ROS can react with NO to form peroxynitrite, thereby reducing NO bioavailability and impairing cellular responses associated with wound healing. In the negative control, the absence of antioxidant protection may have favored oxidative stress and limited fibroblast migration. Conversely, the antioxidant activity of both extracts may have helped preserve NO bioavailability by reducing ROS levels. Under these conditions, O100 may have provided a more favorable balance between ROS scavenging and NO signaling, resulting in enhanced fibroblast migration.

This interaction became more evident under hyperglycemic conditions. Previous studies have shown that elevated glucose concentrations promote ROS accumulation, mitochondrial dysfunction, and the formation of advanced glycation end products, all of which impair fibroblast function and delay wound closure [47,48,49,50]. The inability of either extract alone to fully restore migration suggests that their antioxidant activity was insufficient to overcome the severe oxidative stress associated with hyperglycemia. Interestingly, the partial recovery observed with the O50:T50 treatment points toward a complementary interaction between both species. While O100 was enriched in phenolic acids and flavonoid glycosides and showed stronger migration-promoting effects, T100 exhibited a more diverse phytochemical profile, further enriched by biflavonoids and diterpenoids and displayed greater anti-inflammatory activity through the inhibition of nitric oxide production. Under hyperglycemic conditions, where oxidative stress and inflammation act simultaneously to impair wound repair, the combination of these phytochemical classes may provide broader biological protection than either extract alone. Although these mechanisms remain hypothetical and require direct confirmation through ROS, iNOS, and cytokine analyses, the findings suggest that combining phytochemicals with complementary modes of action may provide greater benefits than individual extracts under conditions of elevated oxidative stress.

## 4. Materials and Methods

### 4.1. Materials and Reagents

Distilled water and commercially prepared ethanol (70%, *v*/*v*; Desarrollo de Especialidades Químicas S.A. de C.V. (DEQ), Monterrey, Nuevo León, Mexico) were used as extraction solvents. Methanol (99.8%) and Ethanol (96%, *v*/*v*) purchased from DEQ were used for antioxidant assays. Folin–Ciocalteu reagent (2 M), sodium carbonate (99.5%), gallic acid (99%), ABTS salt (>98%), DPPH (>90%), potassium persulfate (99.99%), 6-hydroxy-2,5,7,8-tetramethylchroman-2-carboxylic acid (Trolox, 99.5%), and AAPH (99.78%) were purchased from Sigma-Aldrich (St. Louis, MO, USA). LC-MS-grade water and acetonitrile were obtained from Fisher Scientific (Waltham, MA, USA), whereas formic acid was purchased from Sigma-Aldrich (St. Louis, MO, USA). Dulbecco’s Modified Eagle Medium (DMEM), fetal bovine serum (FBS), phosphate-buffered saline (PBS), and 100× penicillin–streptomycin antibiotic solution were obtained from Gibco (Thermo Fisher Scientific, Waltham, MA, USA). Human dermal fibroblasts (HDFa) and RAW 264.7 macrophages were obtained from the American Type Culture Collection (ATCC, Manassas, VA, USA). The Griess Reagent System and CellTiter 96^®^ Aqueous One Solution Cell Proliferation Assay were purchased from Promega (Madison, WI, USA).

### 4.2. Plants Samples

Leaves and branches of *Taxodium mucronatum* were collected in the wild near Monterrey, Nuevo León, México (25°35′45.427″ N, 100°15′44.528″ W) in March 2023. Additionally, dried aerial parts of *Oenothera rosea* L’Hér. ex Aiton, locally known as “hierba del golpe” were purchased from a certified herbal supplier under the brand name EHYA Herbolaria (Casa de Herbolaria, Naucalpan de Juárez, Estado de México). Supplier confirmed compliance with COFEPRIS regulations. Plant identification was performed using standard taxonomic keys and authoritative floristic references for both *Taxodium mucronatum* and *Oenothera rosea* [51,52,53]. Plant names were verified in The Plant List (http://www.theplantlist.org, accessed in 14 March 2023). Representative photographs of the plant material are provided in the Supplementary Materials (S2).

Samples were thoroughly washed with cold water to remove dirt and soil, cut into pieces of approximately 1 inch, freeze-dried and stored at −80 °C until further processing. Prior to extraction, plant material from both species was separated into leaf/flower and stem/branch fractions. As the average proportion of these fractions was similar between species, samples were standardized to the mean leaf/flower:stem/branch ratio of 30:70 (*w*/*w*) for all subsequent analyses.

### 4.3. Solvent Extraction and Experimental Design

A full factorial experimental design was employed to assess the influence of solvent type (70% ethanol and water), extraction temperature (25 °C, 40 °C, and 60 °C), and extraction time (1 h and 24 h) on the extraction efficiency of compounds with antioxidant properties. The resulting 2 × 3 × 2 design comprised 12 treatment combinations, each performed in triplicate as independent extractions. Phytochemical extractions were performed separately for each species, using 4 g of plant material, each processed at a plant-to-solvent ratio of 1:10 (*w*/*v*). Samples were placed in 50 mL sealed tubes to prevent solvent evaporation and contamination and incubated in a shaker set to 250 rpm to ensure uniform mixing and maintained at the specified temperatures. After the extraction process, the supernatants were subjected to vacuum filtration through a 0.11 μm paper filter. The 70% ethanol-containing extracts were further subjected to a direct nitrogen flow to evaporate the organic solvent. Subsequently, all extracts were freeze-dried. Extraction yield was calculated gravimetrically as the ratio of the mass of the dried extract recovered after solvent removal to the initial mass of plant material used for extraction. The yield was expressed as mg of dried extract per g of plant material (mg/g). The dried extracts were reconstituted in the original extraction solvent to a final concentration of 1000 ppm for subsequent antioxidant assays.

### 4.4. In Vitro Chemical Antioxidant Activity

Total phenolic content (TPC) and 2,2-diphenyl-1-picryl-hydrazyl (DPPH) scavenging activity were evaluated in a 96-well microplate according to a previously validated method with slight modifications [54]. For TPC, aliquots of 20 µL from each extract were dispensed into individual wells of a 96-well microplate, followed by the addition of 100 µL of Folin–Ciocalteu reagent diluted 1:10 (*v*/*v*) with distilled water. After 5 min of incubation at room temperature, 80 µL of sodium carbonate solution (7.5%) was added to each well. The microplate was incubated at room temperature for 90 min in the dark. The absorbance was measured at 765 nm using a microplate reader (Biotek, Synergy HT KC4, Winosski, VT, USA). A correction blank containing only the extraction solvent was included to account for background absorbance. Total phenolic content was expressed as mg gallic acid equivalents (GAE) per gram of dry-base extract, using a standard curve prepared with gallic acid. For antioxidant activity, 60 μM solution of DPPH in methanol was prepared. Aliquots (7 µL) of each extract were added to individual wells of a 96-well microplate along with 193 µL of the DPPH solution. The microplate was kept in the dark for 30 min, after which absorbance was measured at 517 nm using a microplate reader and corrected by subtracting the values of the correction blanks. DPPH radical scavenging activity was expressed as µmol Trolox equivalents (TE) per gram of extract (µmol TE/g extract), using a calibration curve prepared with Trolox.

The 2,2′-azino-bis (3-ethylbenzothiazoline)-6-sulfonic acid (ABTS) assay was done following [55] methodology with slight modifications. The ABTS•+ radical cation was produced by reacting 7 mM ABTS stock solution, prepared in PBS, with 2.45 mM potassium persulfate at 1:1 *v*/*v* ratio and allowing the mixture to stand in the dark at room temperature for 14 h before use. The ABTS•+ solution was diluted with ethanol 96% (*v*/*v*) to achieve an absorbance of 0.70 ± 0.02 at 734 nm. Aliquots of 1 µL of each extract were added to individual wells of a 96-well microplate, followed by 180 µL of the diluted ABTS•+ solution. After 15 min of reaction in the dark, the absorbance was measured at 734 nm using a microplate reader and corrected by subtracting the values of the correction blanks. ABTS radical scavenging activity was expressed as µmol Trolox equivalents (TE) per gram of extract (µmol TE/g extract), using a calibration curve prepared with Trolox.

### 4.5. Overall Antioxidant Activity

To determine the combination of extraction conditions that maximizes overall antioxidant activity, the desirability function methodology was employed [56], integrating the results obtained from TPC, DPPH, and ABTS assays. This multi-response optimization technique involves two main steps. First, an individual desirability value (*d_i_*) is calculated for each of the k response variables using the formula:

(1) $$d_{i}=\frac{x-0}{x_{\max}-0}$$

Second, the individual desirability values are aggregated into a composite desirability index (*D*) according to:

(2) D=(d1×d2×…×dk)1k

Individual desirability values (*d_i_*) and the composite desirability index (*D*) range from 0 to 1, where 0 represents a completely undesirable response and 1 represents the most desirable outcome.

### 4.6. Characterization of Phytochemicals by UHPLC-MS/MS

Aliquots of 2 μL (for both ESI positive and ESI negative analyses) were injected into a UHPLC-MS/MS system consisting of an Vanquish Horizon UHPLC (Thermo Fisher Scientific, Bremen, Germany) coupled to an Orbitrap Exploris 120 Mass Spectrometer (Thermo Fisher Scientific, Bremen, Germany). Compounds were separated using a ZORBAX Eclipse Plus C18 (2.1 × 100 mm, 1.8 μm). The mobile phase used in the separation was LC-MS-grade water (solvent A) and acetonitrile (solvent B), each containing 0.1% (*v*/*v*) formic acid (FA). The programmed gradient was 0–7 min (0–30%B), 7–9 min (30–80%B), 9–11 min (80–100%B), 11–14 min (100%B), and the solvent system operated at a flow rate of 0.5 mL/min. The column temperature was maintained at 40 °C, and the acquisition wavelength range of the photo diode array (PDA) was set at 190–680 nm. Initially, the samples were analyzed in full scan mode at a resolution of 60,000 with a scan range of 60–900 *m*/*z*. This was followed by MS/MS analysis, performed at a resolution of 15,000, and CID was applied with energies set at 20%, 30%, and 40%. Mass spectra were acquired and processed using Thermo Scientific Xcalibur v4.7 (Thermo Fisher Scientific, Bremen, Germany) and processed using Thermo FreeStyle v1.8 (Thermo Fisher Scientific, Waltham, MA, USA).

The raw MS data were processed using MZmine (version 4.5.20) for feature detection, deconvolution, isotope grouping, and alignment. Chromatographic peaks were detected using the ADAP chromatogram builder, followed by feature linking across samples. For metabolite annotation, in-house libraries containing accurate mass (*m*/*z*) and retention time (RT) of 416 authentic reference standards analyzed under the same chromatographic conditions were used in conjunction with public and commercial MS/MS spectral databases. An MSP library was generated by combining spectra from the NIST23 MS/MS database and MassBank of North America (MoNA; http://massbank.us, accessed in 30 October 2025). Experimental MS/MS spectra were matched against the combined MSP library and the in-house *mz*RT database to support compound annotation. Features that could not be confidently assigned through spectral matching were further evaluated using SIRIUS v. 6.1.1 (University of Jena, Jena, Germany), which was employed for molecular formula prediction and compound classification based on fragmentation trees and in silico databases. Metabolites were annotated according to Schymanski’s confidence framework [57]. MSI level 1 was assigned to metabolites with precursor *m*/*z*, in-house *mz*RT library matching, and MS/MS spectral library matching. MSI level 2a was assigned to metabolites with precursor *m*/*z* and MS/MS spectral library matching against a private spectral library (NIST23). MSI level 2b was assigned to metabolites with precursor *m*/*z* and MS/MS spectral library matching against public spectral databases, including MoNA. MSI level 3 was assigned to tentative structural annotations supported by spectral evidence but with unresolved structural ambiguity, including positional and stereoisomers. MSI level 4 was assigned to features for which only the molecular formula could be determined. An additional Supplementary File provides the complete MS/MS spectra for all relevant features discussed in this study (Supplementary File S1).

### 4.7. Cellular Antioxidant Activity Assay

Cellular antioxidant activity (CAA) was assessed using the methodology of [58] with slight modifications. HDFa were cultured in DMEM with 5% FBS under standard conditions (37 °C, 5% CO_2_) and used at passages 38–40. Cells were seeded in 96-well plates (50,000 cells/well) and incubated overnight. After washing with PBS, treated wells received 100 µL of extract (50 µg/mL) mixed 1:1 with DCFH-DA, 60 µM, and incubated for 20 min. Cells were then washed and exposed to 500 µM of 2,20-azobis (2-methylpropionamidine) dihydrochloride (AAPH) to induce oxidative stress. Blank wells contained cells, but neither DCFH-DA nor AAPH was added. Positive control wells contained DCFH-DA and AAPH without extracts. Negative control wells contained DCFH-DA without AAPH or extracts. Fluorescence (Excitation 485 nm/Emission 538 nm) was recorded every 2 min for 90 min at 37 °C.

CAA is expressed as relative area under the curve (AUC), normalized to the oxidation control:

(3) $$\mathrm{CAA}\;(\mathrm{relative}\;\mathrm{AUC}\;\%)=\frac{\int \mathrm{SA}-\int \mathrm{BA}}{\int \mathrm{CA}-\int \mathrm{BA}}$$

where ∫SA is the integrated area of the treated cells fluorescence during the 90 min of the reading, ∫CA is the integrated area of the positive control, and ∫BA blanks integrated area mean.

### 4.8. Nitric Oxide Production Assay for Anti-Inflammatory Activity

Nitric oxide (NO) production was assessed following the protocol described by [59]. RAW 264.7 mouse macrophages were seeded in a 96-well plate at a density of 50,000 cells per well in 100 µL of DMEM supplemented with 10% FBS and 1% antibiotic and incubated overnight. Subsequently, 50 µL of extract were added to the cells to achieve a final concentration of 20 µg/mL, and the plate was incubated for 24 h. To induce nitric oxide production, lipopolysaccharide (LPS) from E. coli was added to designated wells at a final concentration of 4 µg/mL. Experimental groups included: (i) LPS-stimulated cells without extract treatment (positive control), representing maximal nitric oxide production; (ii) non-stimulated cells without extract treatment (negative control); (iii) LPS-stimulated cells treated with extracts (treatment groups); and (iv) non-stimulated cells treated with extracts (extract controls) to evaluate any effects unrelated to the inflammatory stimulus. After 24 h of incubation, 100 μL of supernatant from each well was transferred to a separate 96-well microplate to measure nitrite concentration using the Griess Reagent System, following the manufacturer’s instructions. Absorbance was measured at 550 nm using a microplate reader (Biotek, Synergy HT KC4, Winoski, VT, USA). The results were expressed as nitrite production levels (µM). The remaining cells on the plate were treated using the CellTiter Assay.

### 4.9. In Vitro Fibroblast Migration Assay

To assess potential wound closure, spontaneous migration of fibroblasts was performed according to [60] with some modifications. Briefly, inserts were placed in the center of the well of a 24-well plate and 70 μL HDFa cell suspension (500,000 cells/mL) were placed in each insert well. Cells were incubated for 24 h at 37 °C, 5% CO_2_. Culture inserts were carefully removed, and cellular debris was rinsed with PBS. Cells were incubated with 500 μL of the extract mixture at a final concentration of 20 µg/mL, prepared separately in either standard-glucose medium or high-glucose medium, both without FBS, while untreated cells served as controls. At this concentration, all treatments maintained cell viabilities above 92%. Immediately after treatment application, images of the gaps were captured using an inverted microscope; additional images were taken at 24, 48, and 72 h to monitor cell migration into the gap area. ImageJ software (version 1.54r) was used to measure the area of cells covering the gap at each time point. Results were reported as a percentage of wound closure using the following formula, where *A_i_* is the initial gap area, and *A_t_* is the empty area at each time.

(4) $$\%\;\mathrm{wound}\;\mathrm{closure}=\frac{\left(A_{i}-A_{t}\right)}{A_{i}}$$

### 4.10. Statistical Analysis

Data were obtained from at least three independent extractions. Statistical analyses were performed using JMP student edition 18 (SAS Institute Inc., Cary, NC, USA) software. A three-way analysis of variance (ANOVA) was used to evaluate the effects of temperature, solvent type, and extraction time on extraction yield. Differences in desirability scores among experimental conditions were evaluated using one-way ANOVA, followed by Tukey’s honestly significant difference (HSD) post hoc test to identify significant differences between combinations of extraction conditions. Results were considered statistically significant at *p* < 0.05.

## 5. Conclusions

This study highlights the wound-healing potential of *Oenothera rosea* and *Taxodium mucronatum*, two plants traditionally used in Mexico for this purpose. The most effective extraction conditions were 24 h at 60 °C with 70% ethanol, which produced the highest antioxidant activity in both species. Chemical profiling showed that *O. rosea* is abundant in phenolic acids and flavonoid glycosides, while *T. mucronatum* contains biflavonoids, flavan-3-ols, and diterpenoids. These distinct phytochemical profiles were reflected in their biological activities. *O. rosea* displayed notable antioxidant activity and significantly promoted fibroblast migration under normal glucose levels, although it did not inhibit nitric oxide (NO). *T. mucronatum* also showed strong antioxidant effects and inhibited NO in macrophages, with fibroblast migration higher than the control but lower than *O. rosea*. Under hyperglycemic conditions, only the 1:1 mixture of both extracts partially supported fibroblast migration, suggesting that their combined action may balance NO regulation and reactive oxygen species scavenging.

It is noteworthy that these plants have traditionally been used for the treatment of acute wounds; however, there are no previous records of their application in diabetic foot ulcers. This study highlights the potential benefit of combining both extracts, whose synergistic mechanisms may be especially valuable in the highly stressed biochemical environment of diabetes, where oxidative stress, impaired cell migration, and chronic inflammation hinder healing. This evidence supports the possibility that the combined use of *T. mucronatum* and *O. rosea* extracts could represent a promising strategy for enhancing wound repair in diabetic foot ulcers, warranting further exploration in preclinical and clinical settings.

## Figures and Tables

**Table 1:** Yield of *O. rosea* and *T. mucronatum* extracts under different solvent, temperature, and time conditions.

			*Oenothera rosea*		*T. mucronatum*	
Solvent	t (h)	T (°C)	Run	Yield (mg/g Sample, DW)	Run	Yield (mg/g Sample, DW)
Water	1	25	1	97.3 ± 5.1 ^cde^	13	69.1 ± 1.8 ^cd^
		40	2	103.2 ± 7.2 ^bcd^	14	67.4 ± 4.4 ^cd^
		60	3	117.0 ± 1.4 ^bc^	15	75.9 ± 4.1 ^bcd^
	24	25	4	119.8 ± 6.2 ^abc^	16	62.3 ± 4.5 ^d^
		40	5	125.3 ± 12.0 ^ab^	17	75.8 ± 4.1 ^bcd^
		60	6	141.7 ± 19.0 ^a^	18	76.6 ± 10.1 ^bcd^
70% EtOH	1	25	7	42.6 ± 2.4 ^g^	19	87.5 ± 6.7 ^bc^
		40	8	40.0 ± 2.5 ^g^	20	82.8 ± 6.4 ^bcd^
		60	9	54.8 ± 4.1 ^fg^	21	71.9 ± 5.9 ^cd^
	24	25	10	76.3 ± 1.5 ^ef^	22	93.6 ± 7.2 ^b^
		40	11	80.8 ± 8.0 ^de^	23	93.0 ± 8.8 ^b^
		60	12	104.7 ± 6.6 ^bc^	24	117.3 ± 13.0 ^a^

Different letters indicate differences among treatments (columns) using Tukey test (*p* < 0.05). t, time; T, temperature; EtOH, ethanol; DW, dry weight.

**Table 2:** Antioxidant activity assays from *Oenothera rosea*.

Run	TPC (mg GAE/g Extract, DW)	DPPH (μmol Trolox/g Extract, DW)	ABTS (μmol Trolox/g Extract, DW)
1	175.5 ± 4.6 ^cd^	75.7 ± 0.8 ^b^	157.6 ± 16.6 ^ef^
2	163.1 ± 2.0 ^de^	45.6 ± 2.4 ^f^	193.3 ± 8.3 ^cde^
3	203.6 ± 4.0 ^b^	55.1 ± 4.2 ^de^	170.4 ± 8.7 ^def^
4	210.6 ± 11.2 ^ab^	80.7 ± 2.4 ^b^	140.5 ± 14.6 ^f^
5	184.9 ± 9.9 ^c^	80.7 ± 2.3 ^b^	194.9 ± 9.1 ^cde^
6	163.8 ± 1.8 ^de^	48.2 ± 1.7 ^ef^	202.8 ± 10.6 ^bcd^
7	97.3 ± 1.1 ^h^	66.1 ± 3.2 ^c^	185.2 ± 11.7 ^cde^
8	90.9 ± 4.2 ^h^	49.6 ± 1.8 ^ef^	182.7 ± 6.1 ^cde^
9	116.9 ± 6.0 ^g^	60.0 ± 3.4 ^cd^	192.0 ± 10.1 ^cde^
10	135.2 ± 3.9 ^f^	84.2 ± 4.9 ^b^	219.9 ± 19.2 ^bc^
11	148.8 ± 5.5 ^ef^	82.6 ± 2.9 ^b^	239.2 ± 28.0 ^ab^
12	220.2 ± 1.3 ^a^	99.0 ± 5.1 ^a^	268.4 ± 2.0 ^a^

TPC: total phenolic content; DPPH: 2,2-diphenyl-1-picrylhydrazyl; ABTS: 2,2′-azino-bis(3-ethylbenzothiazoline-6-sulfonic acid; DW: dry weight. Different letters indicate differences among treatments (columns) using Tukey test (*p* < 0.05).

**Table 3:** Antioxidant activity assays from *Taxodium mucronatum*.

Run	TPC (mg GAE/g Extract, DW)	DPPH (μmol Trolox/g Extract, DW)	ABTS (μmol Trolox/g Extract, DW)
13	83.3 ± 4.2 ^efg^	36.5 ± 0.3 ^e^	145.1 ± 6.7 ^e^
14	92.1 ± 3.0 ^e^	46.5 ± 1.1 ^d^	152.6 ± 3.4 ^e^
15	88.9 ± 1.4 ^ef^	53.8 ± 0.5 ^c^	133.7 ± 3.0 ^e^
16	52.1 ± 2.1 ^h^	30.2 ± 1.5 ^f^	143.0 ± 7.5 ^e^
17	70.3 ± 1.3 ^g^	40.7 ± 0.8 ^e^	133.1 ± 8.0 ^e^
18	75.5 ± 4.8 ^fg^	37.6 ± 0.7 ^e^	132.7 ± 7.0 ^e^
19	189.6 ± 14.7 ^a^	77.7 ± 1.0 ^b^	246.3 ± 5.3 ^c^
20	167.8 ± 2.4 ^b^	84.1 ± 3.4 ^a^	216.2 ± 12.4 ^d^
21	143.4 ± 4.1 ^cd^	76.0 ± 1.3 ^b^	229.2 ± 1.6 ^cd^
22	137.9 ± 2.4 ^d^	83.4 ± 0.6	274.7 ± 17.6
23	152.6 ± 1.5 ^cd^	73.0 ± 4.5	232.9 ± 5.6
24	157.9 ± 0.7 ^bc^	88.8 ± 2.1	316.8 ± 12.4

TPC: total phenolic content; DPPH: 2,2-diphenyl-1-picrylhydrazyl; ABTS: 2,2′-azino-bis(3-ethylbenzothiazoline-6-sulfonic acid; DW: dry weight. Different letters indicate differences among treatment conditions using Tukey test (*p* < 0.05).

**Table 4:** Tentative compounds identified in *Oenothera rosea* through MS/MS comparison.

No	Tentative Compound	RT (UV)	Formula	*m*/*z* [M − H]^−^	Mass Error (ppm)	Chemical Structure or Compound Class	ID Level (Ref)
1	Gallic acid	1.76 (270)	C_7_H_6_O_5_	169.01398	−1.657	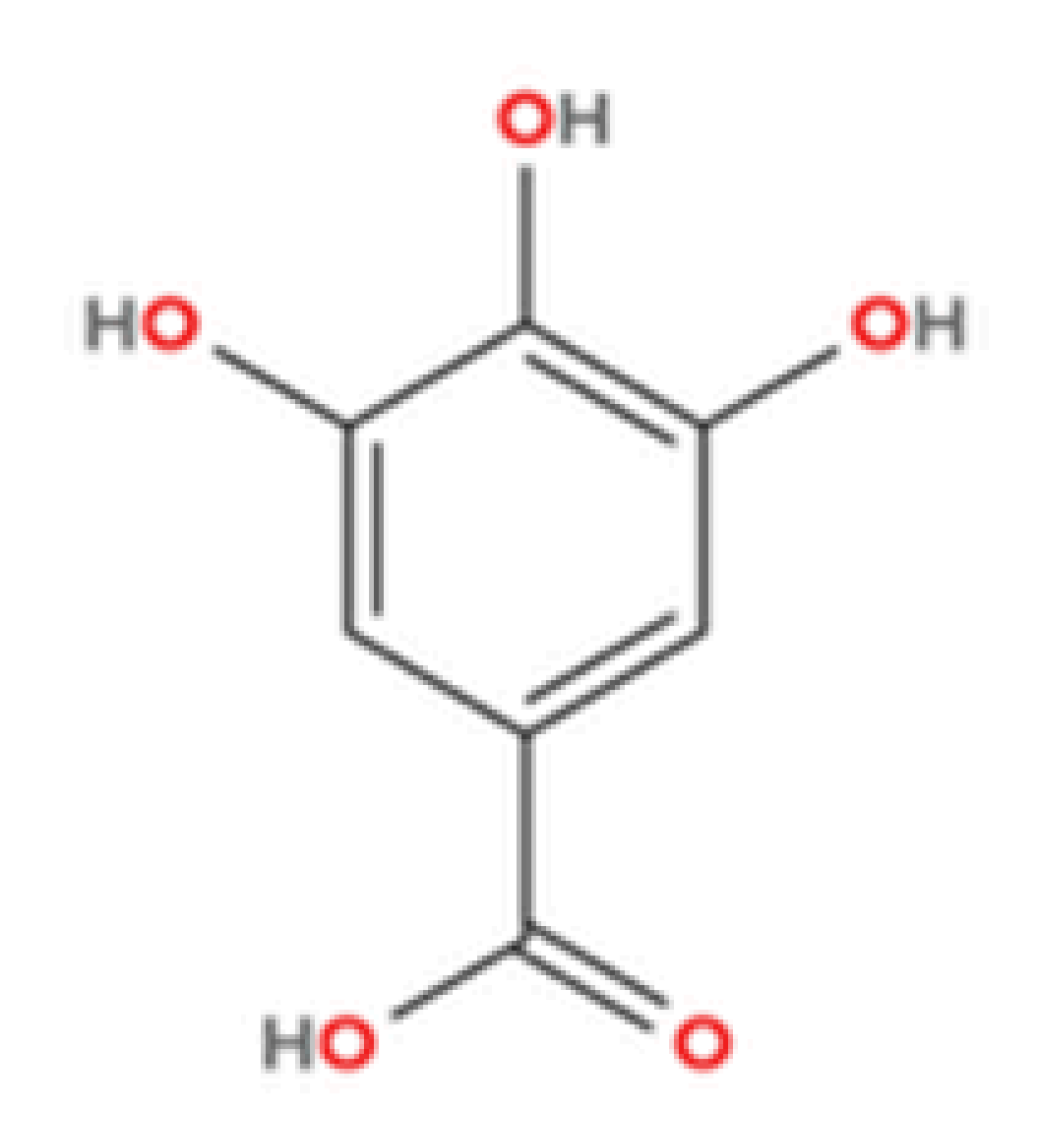	1 (IHL; NIST)
2	Benzoic acid + 2O, *O*-Hex	2.31 (270)	C_13_H_16_O_9_	315.07191	−0.952	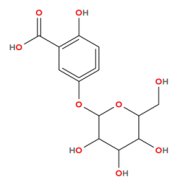	3 (NIST, u.s.)
3	Protocatechuic acid	2.51 (260, 290)	C_7_H_6_O_4_	153.01923	−2.941	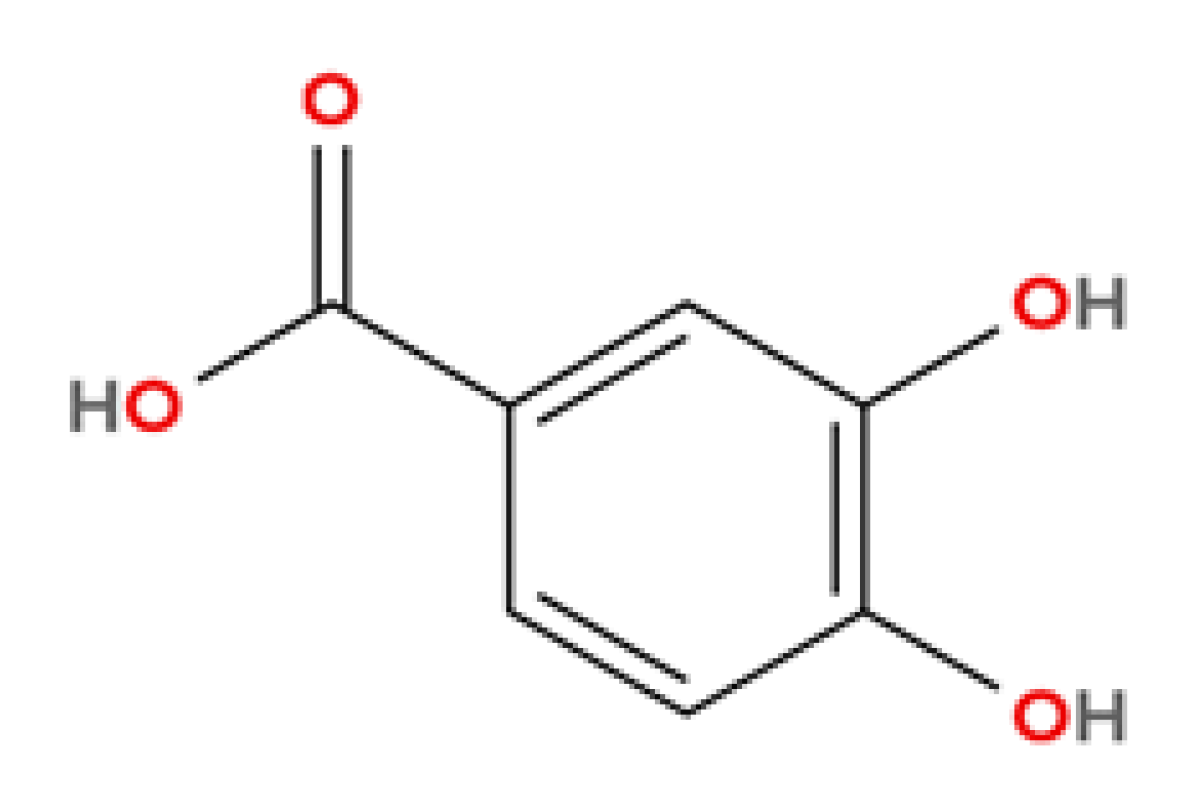	1 (IHL; NIST)
4	Caftaric acid	2.85 (300, 326)	C_13_H_12_O_9_	311.04055	−0.772	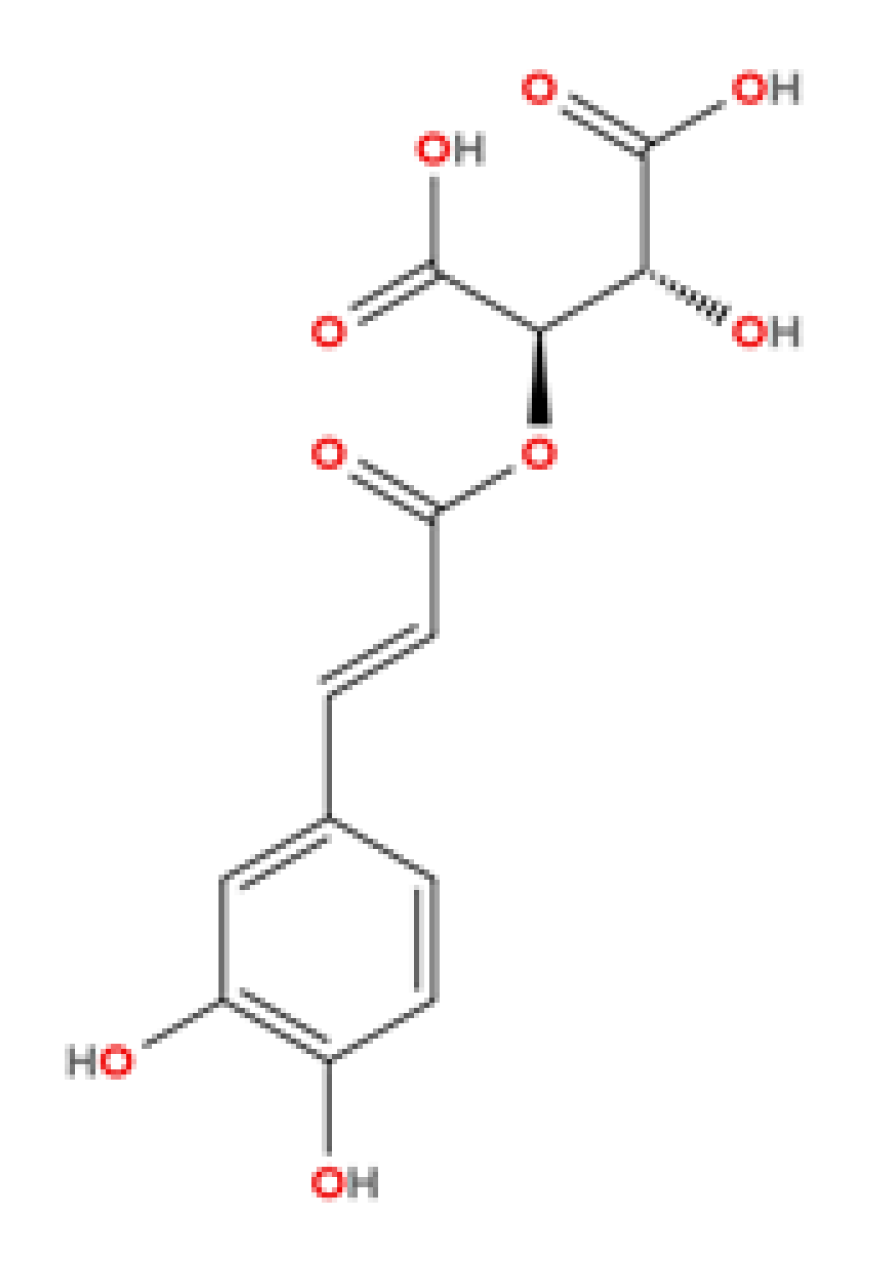	2a (NIST)
5	Neochlorogenic acid	2.97 (286, 322)	C_16_H_18_O_9_	353.08700	0.736	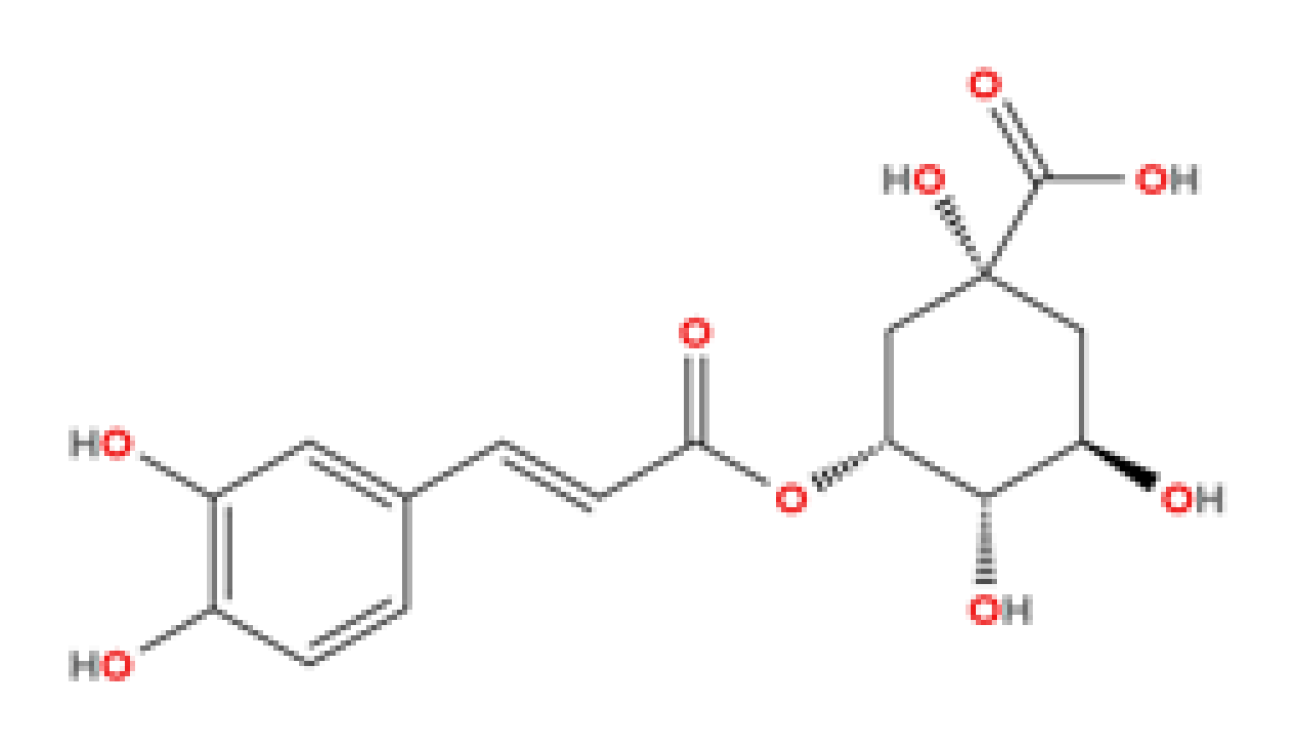	2a (IHL; NIST)
6	Unknown	3.44 (266, 338)	C_9_H_8_O_3_	163.03996	0.123	Coumaric acid derivative	4 (SIRIUS)
7	Unknown	3.88 (266)	C_13_H_8_O_8_	291.01446	−1.271	Benzopyran	4 (SIRIUS)
8	Sulfo jasmonate	4.04 (265)	C_12_H_18_O_7_S	305.06982	−1.049	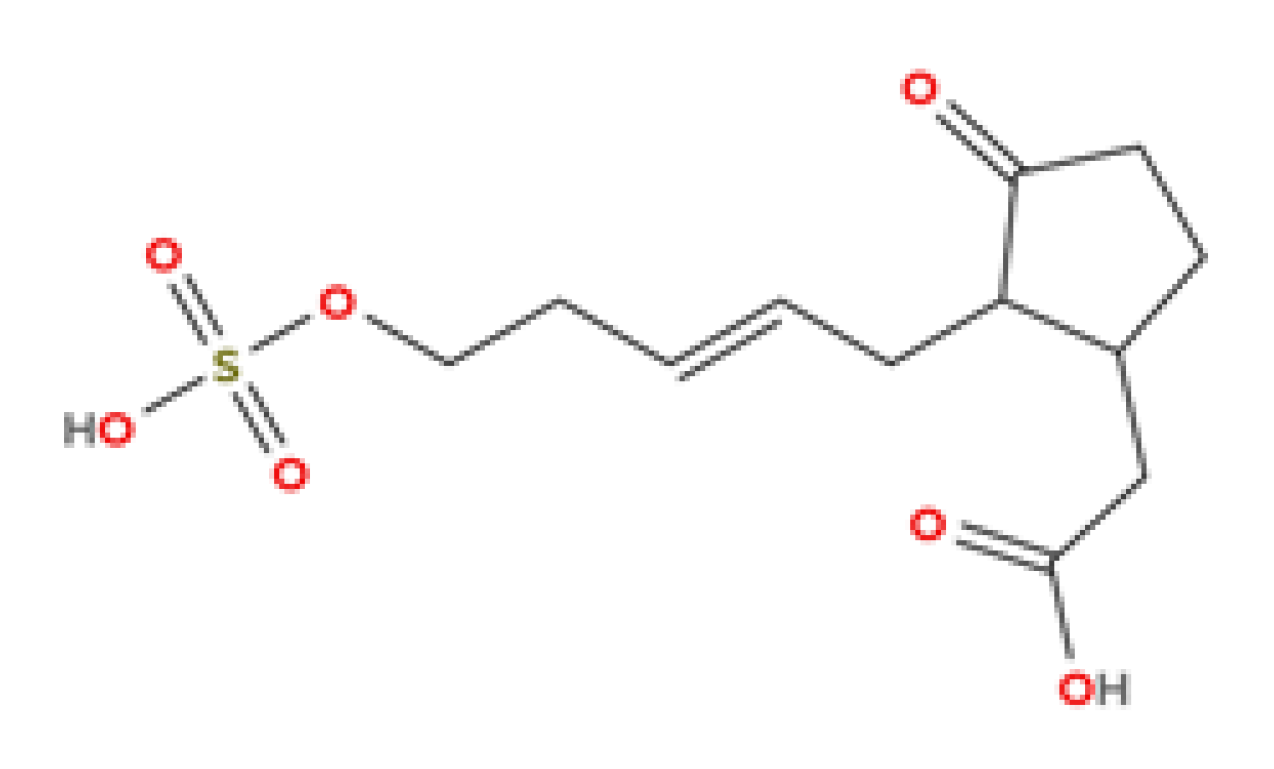	2b (MoNA)
9	4-*O*-*p*-Coumaroylquinic acid	4.42 (262)	C_16_H_18_O_8_	337.09251	−0.504	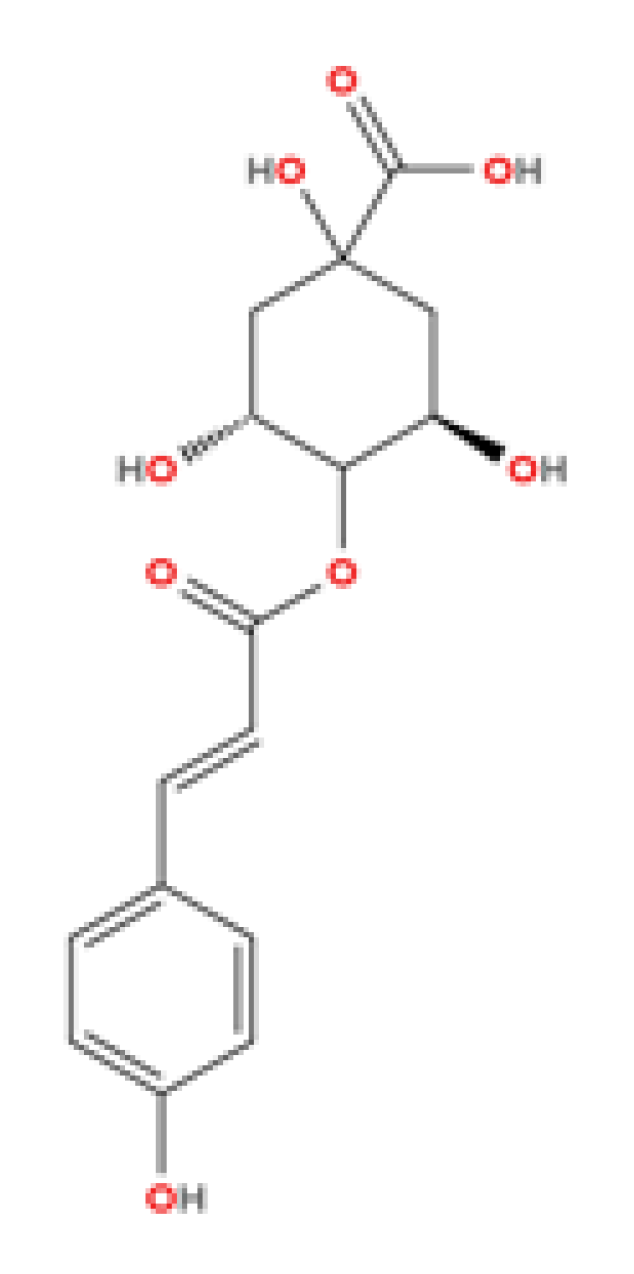	2a (NIST)
10	Ethyl gallate	4.81 (265)	C_9_H_10_O_5_	197.04537	−1.878	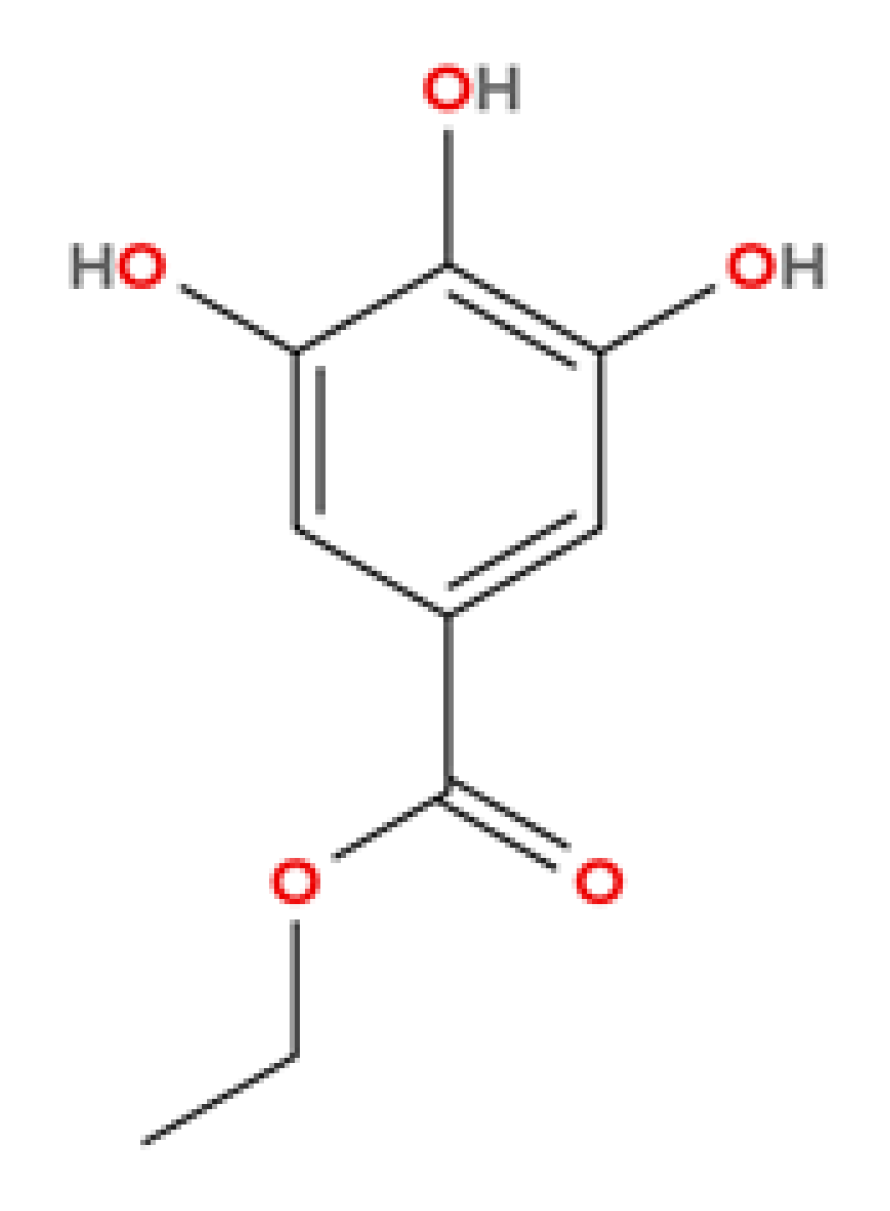	1 (IHL; NIST)
11	Isomyricitrin	5.03 (260, 354)	C_21_H_20_O_13_	479.08235	0.459	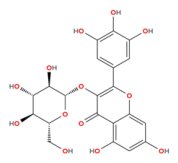	2b (MoNA)
12	2″-*O*-Galloylhyperin	5.33 (262, 354)	C_28_H_24_O_16_	615.09838	0.374	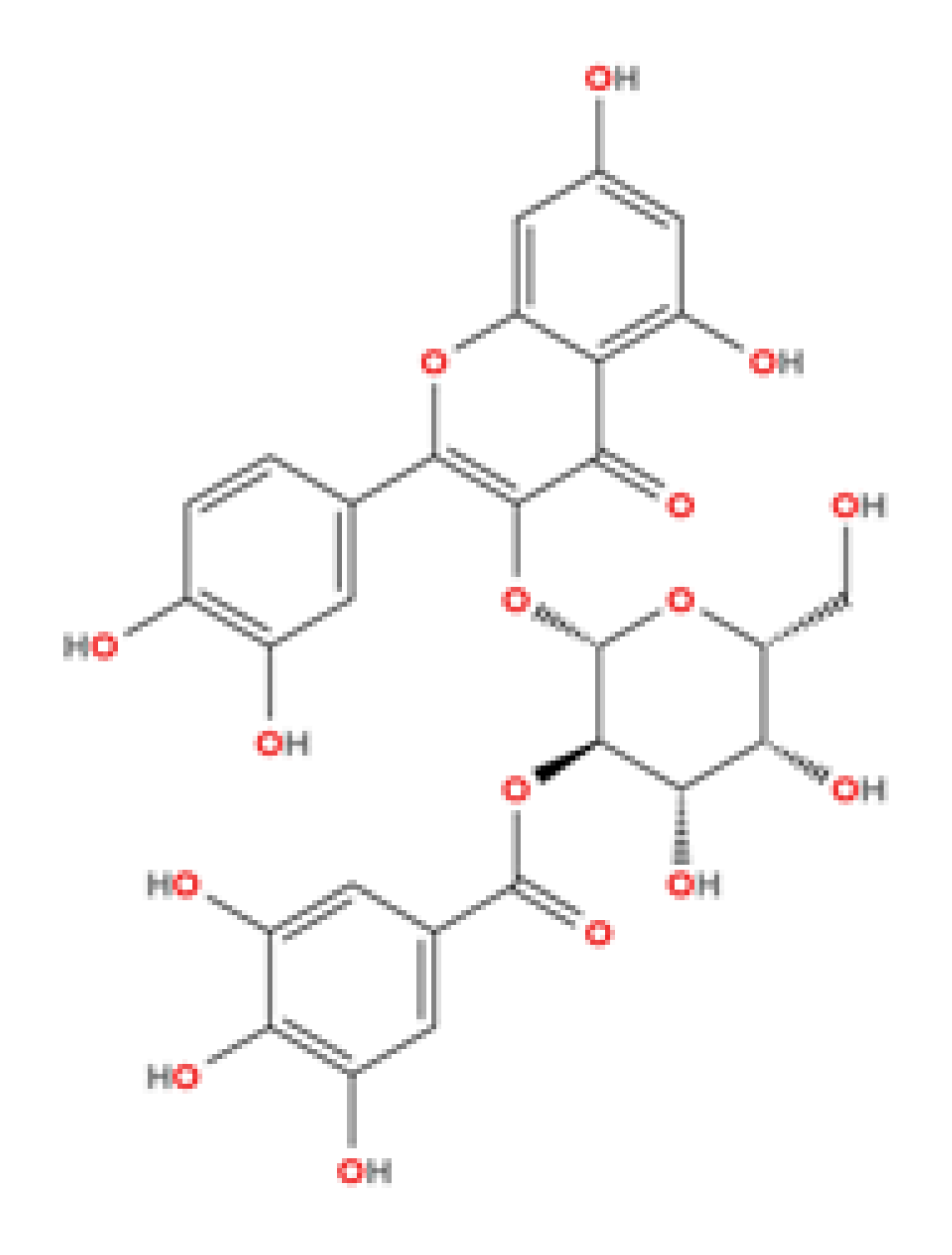	2a (NIST)
13	Quercetin-3′-*O*-sulfate	5.53 (254, 350)	C_15_H_10_O_10_S	380.99119	1.181	3-Sulfated flavonoid	4 (SIRIUS)
14	Delphinidin 3-*O*-rutinoside	5.53 (254, 350)	C_27_H_30_O_16_	609.14520	0.591	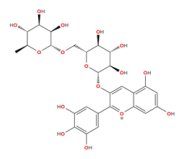	2b (MoNA)
15	Isoquercetin/ Hyperoside	5.62 (254, 354)	C_21_H_20_O_12_	463.0868	1.836	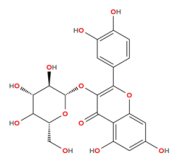	3 (NIST, u.s.)
16	Guaiaverin	6 (254, 350)	C_20_H_18_O_11_	433.0769	0.438	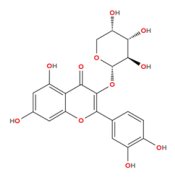	2a (IHL; NIST)
17	Quercitrin	6.28 (255, 350)	C_21_H_20_O_11_	447.09223	1.655	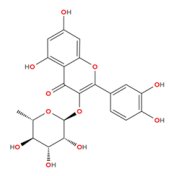	2a (NIST)
18	Quercetin 3-*O*-glucosyl-6″-acetate	6.6 (268, 354)	C_23_H_22_O_13_	505.09798	0.475	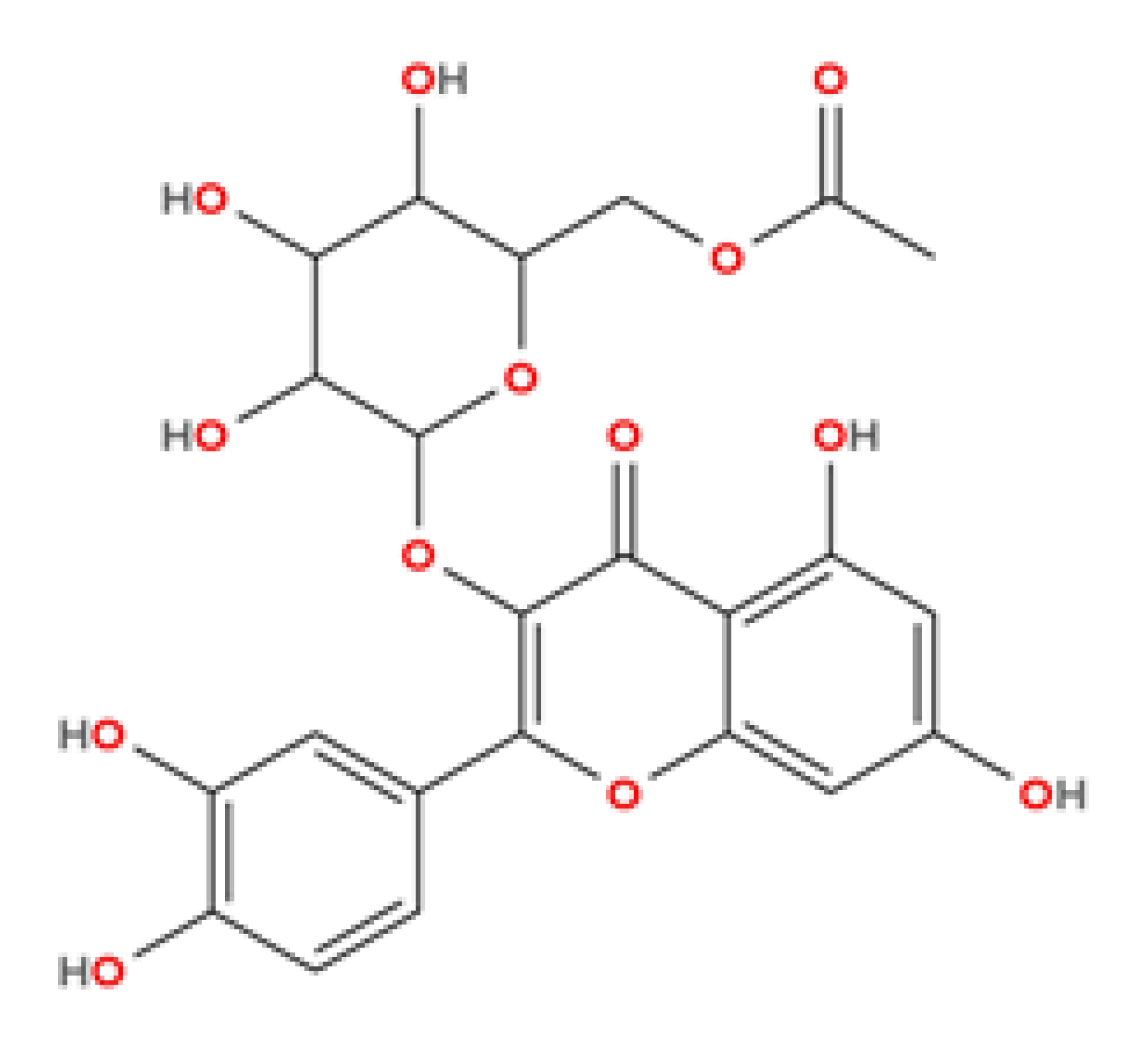	2a (NIST)
19	Afzelin	6.9 (265, 356)	C_21_H_20_O_10_	431.09749	0.765	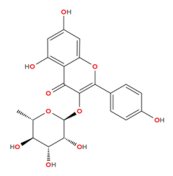	2a (NIST)
20	Kaempferol 7-(6″-*p*-succinylglucoside)	7.22 (265, 356)	C_25_H_24_O_14_	547.10863	0.274	Flavonoid-3-O-glycoside	4 (SIRIUS)
21	Quercetin 3-(3,6″-diacetylgalactoside)	7.65 (268, 356)	C_25_H_24_O_14_	547.10876	0.037	Flavonoid-3-O-glycoside	4 (SIRIUS)
22	Quercetin 3-(2″,3″,4″-triacetylgalactoside)	8.37 (265, 354)	C_27_H_26_O_15_	589.11867	1.154	Flavonoid-3-O-glycoside	4 (SIRIUS)

**Table 5:** Tentative compounds identified in *Taxodium mucronatum* through MS/MS.

No	Tentative Compound	RT (UV)	Formula	*m*/*z* [M − H]^−^	Mass Error (ppm)	Chemical Structure or Compound Class	ID Level (Ref)
1	Procyanidin B	3.38 (280, 320)	C_30_H_26_O_12_	577.1342	0.693	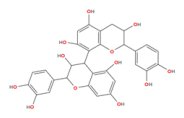	3 (NIST, u.s.)
2	Epicatechin	3.64 (280, 320)	C_15_H_14_O_6_	289.07132	−0.381	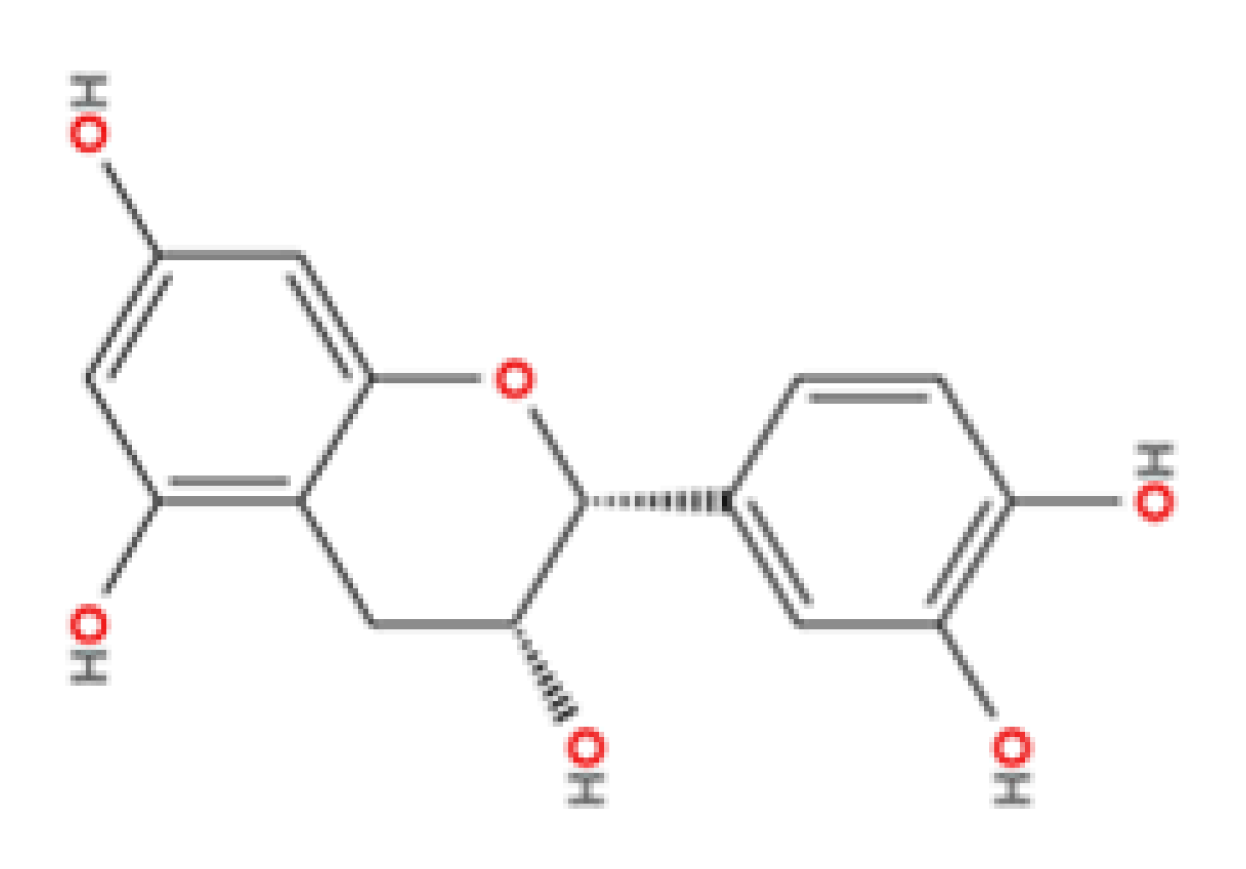	1 (IHL; NIST)
3	Procyanidin B	4.07 (280, 306)	C_30_H_26_O_12_	577.13445	0.260	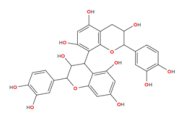	3 (NIST, u.s.)
4	Catechin	4.33 (278)	C_15_H_14_O_6_	289.07142	−0.726	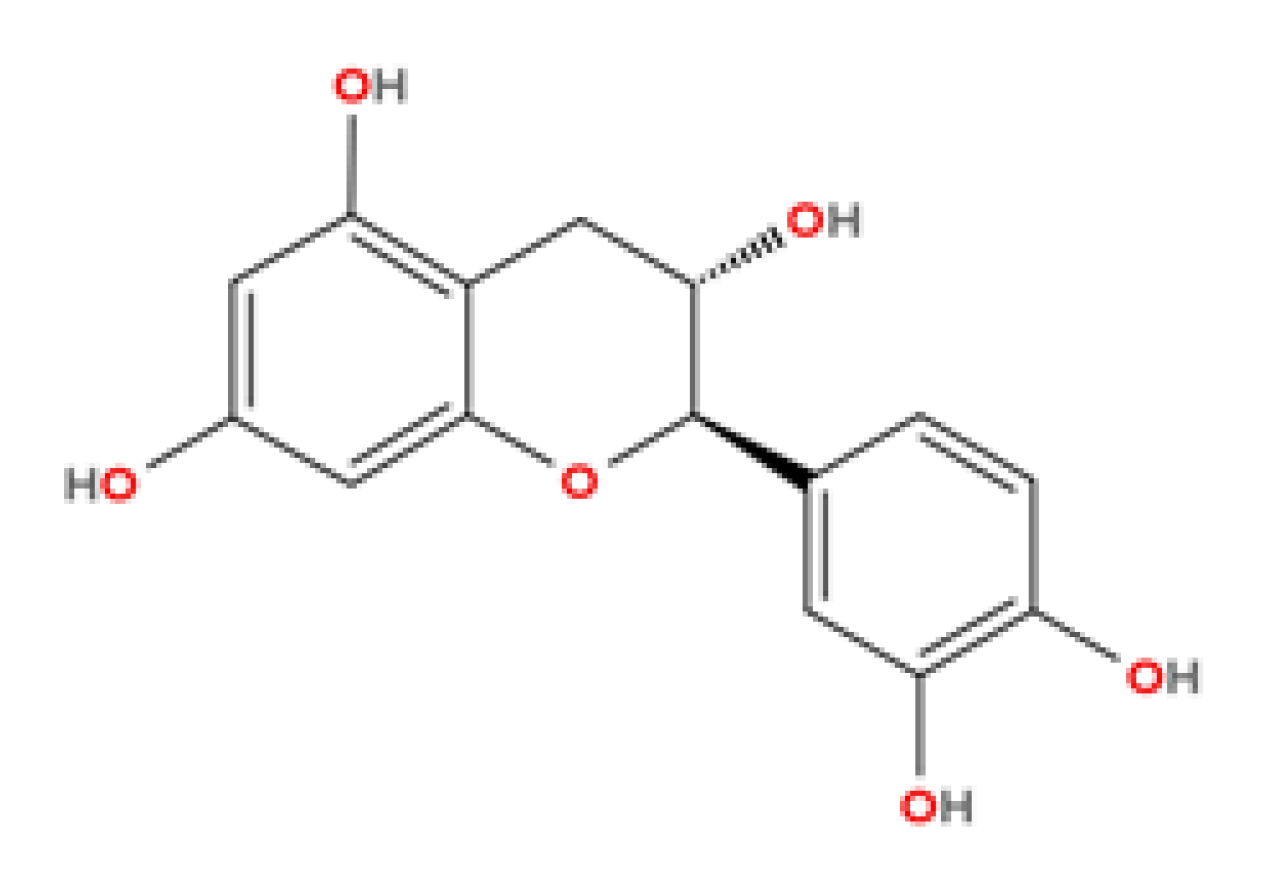	1 (IHL; NIST)
5	Unknown	4.45 (266, 346)	C_23_H_28_O_9_	447.1655	0.022	Flavonoid-*O*-glycoside	4 (SIRIUS)
6	Unknown	4.81 (280, 306)	C_25_H_34_O_12_	525.19713	0.133	Iridoid-O-glycoside	4 (SIRIUS)
7	Quercetin-3-*O*-vicianoside	5.3 (278, 344)	C_26_H_28_O_16_	595.1298	0.185	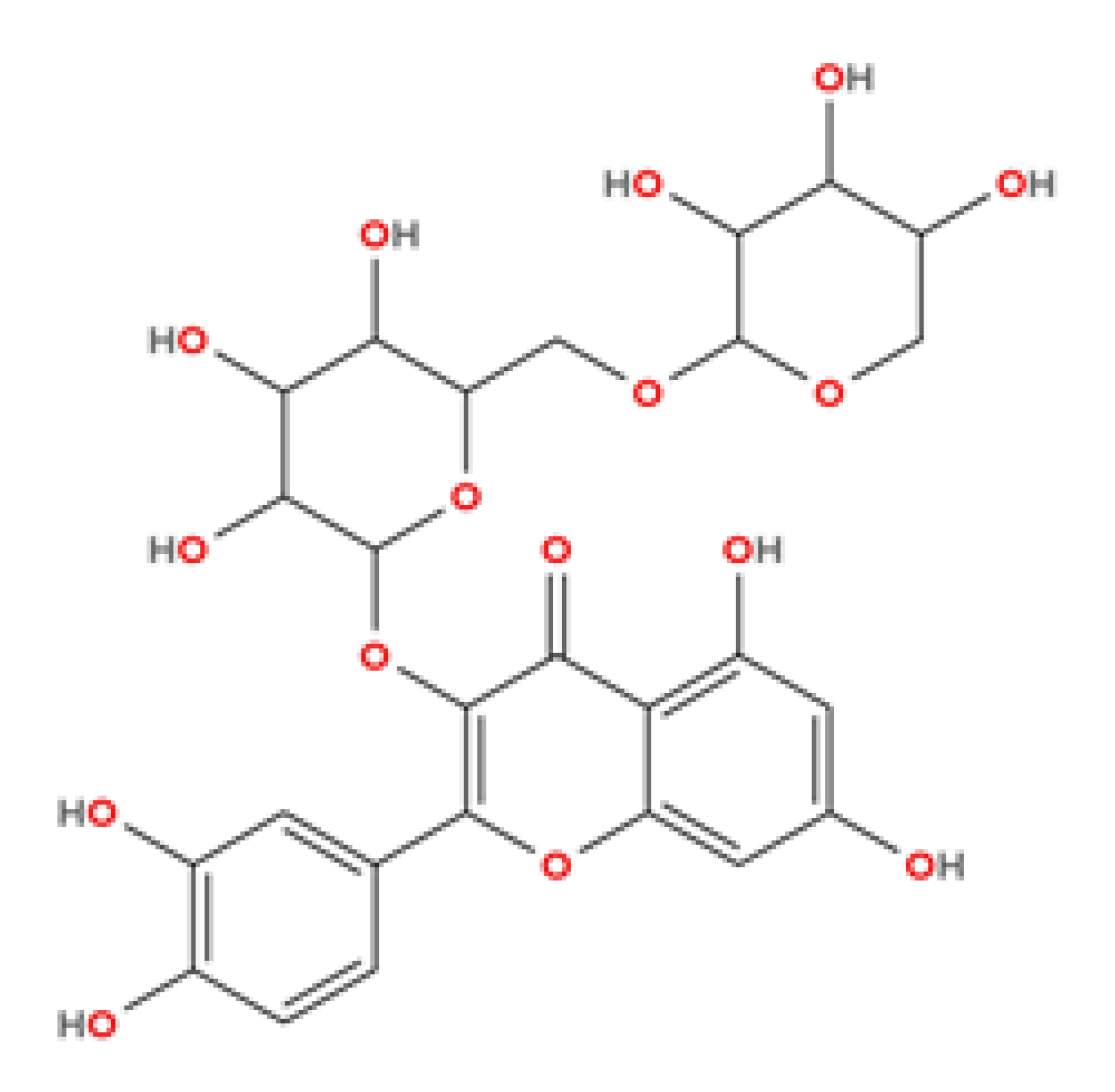	2a (NIST)
8	Isoquercetin/ Hyperoside	5.63 (262, 354)	C_21_H_20_O_12_	463.08716	1.058	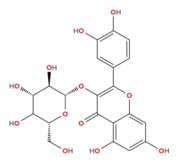	3 (NIST, u.s.)
9	Reynoutrin	6.04 (260, 360)	C_20_H_18_O_11_	433.07676	0.762	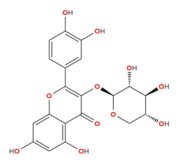	3 (NIST, u.s.)
10	Quercitrin	6.27 (260, 348)	C_21_H_20_O_11_	447.09206	1.521	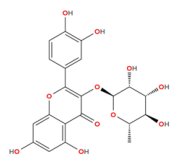	2a (NIST)
11	Kaempferol-3-*O*-rhamnoside	6.91 (278, 344)	C_21_H_20_O_10_	431.09765	0.394	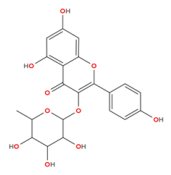	3 (NIST, u.s.)
12	Unknown	7.04 (270, 340)	C_22_H_22_O_11_	461.10827	0.260	Flavonoid-3-O-glycoside	4 (SIRIUS)
13	Quercetin	7.68 (265, 340)	C_15_H_10_O_7_	301.03523	−1.329	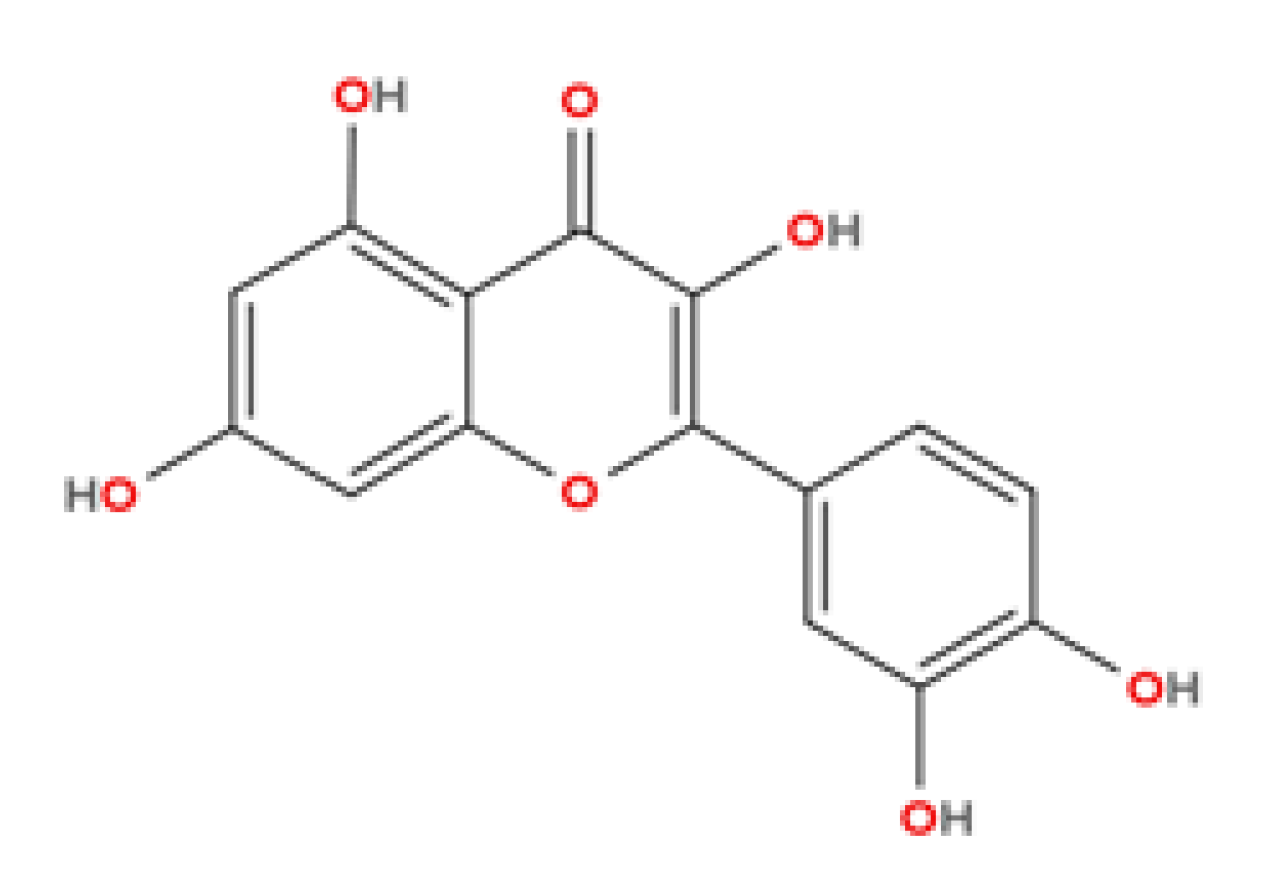	2a (IHL; NIST)
14	Naringenin	8.21 (278)	C_15_H_12_O_5_	271.06105	−1.476	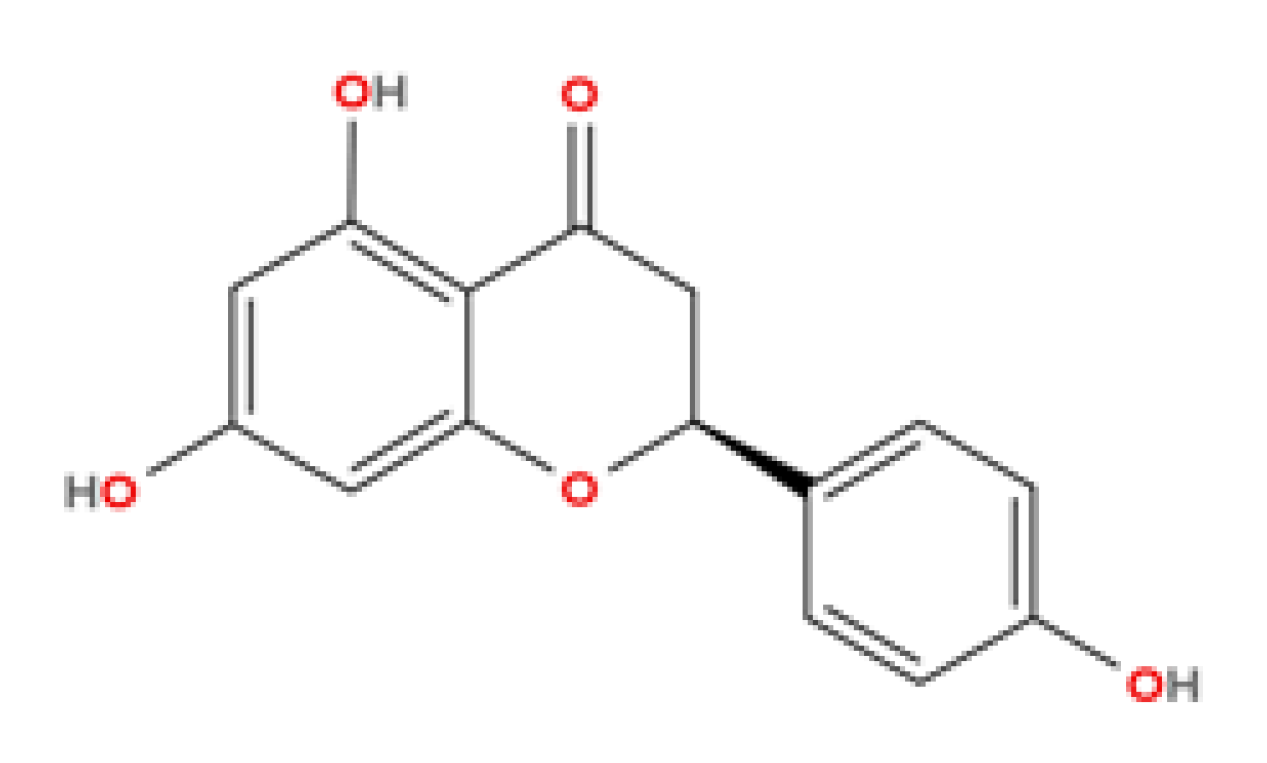	2a (IHL; NIST)
15	Cupressuflavone	8.66 (274, 330)	C_30_H_18_O_10_	537.08206	0.205	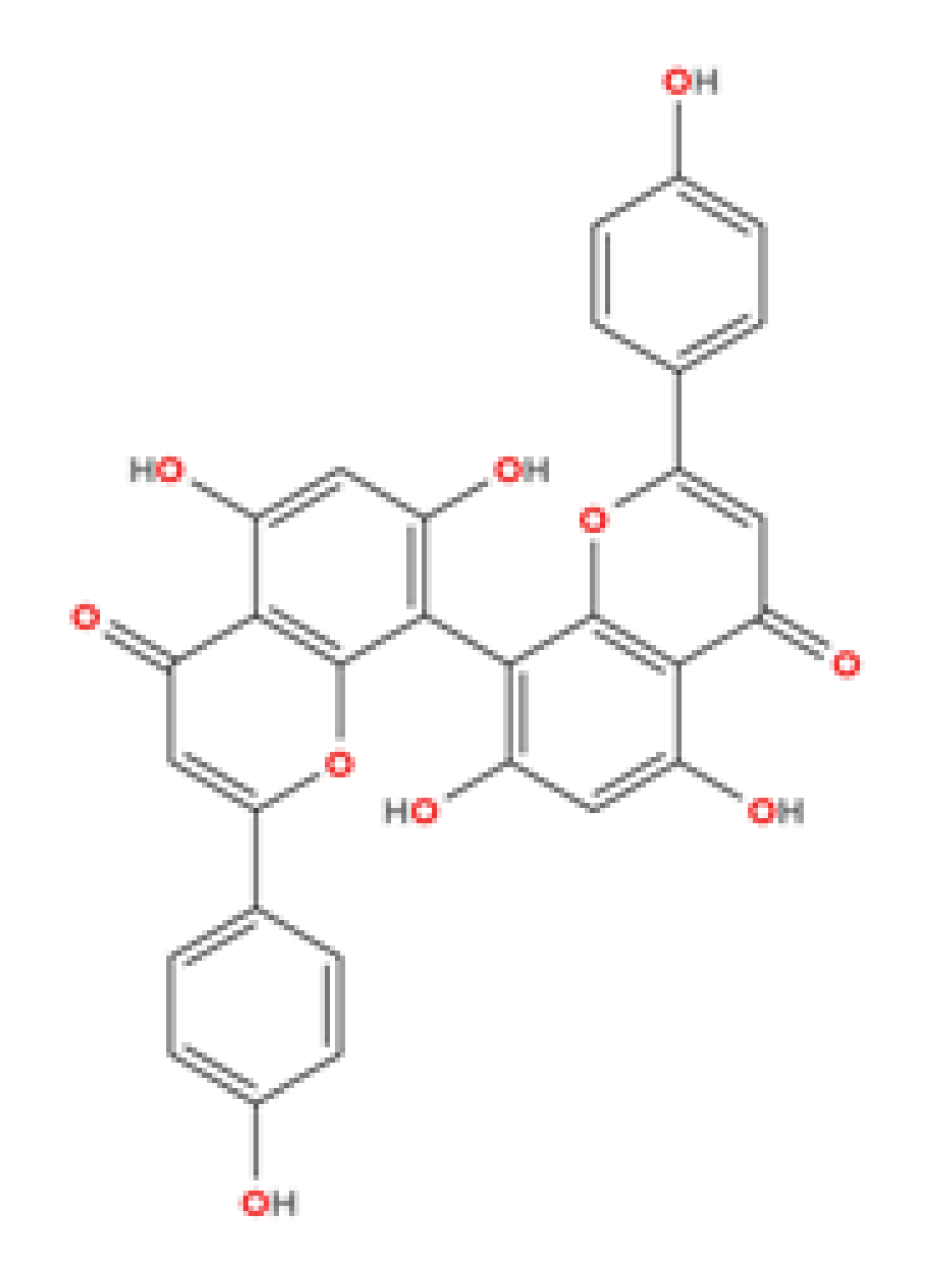	3 (NIST, u.s.)
16	Unknown	8.87 (275, 340)	C_32_H_24_O_12_	347.18564	0.605	Diterpene lactone	4 (SIRIUS)
17	Hinokiflavone	9.04 (270, 336)	C_30_H_18_O_10_	537.08179	0.708	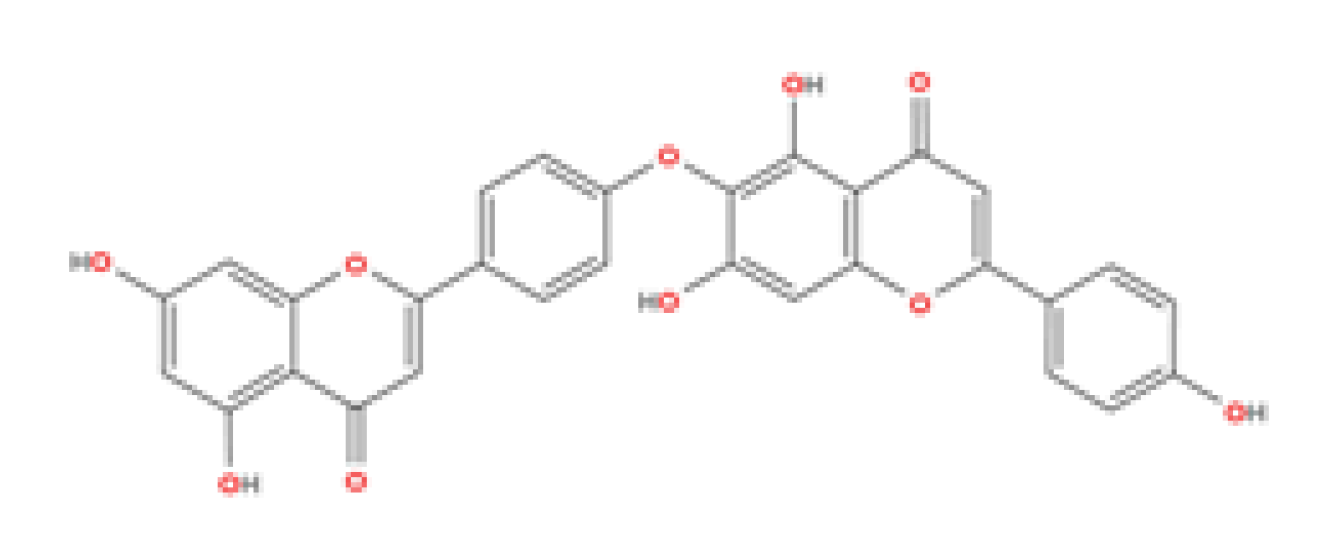	3 (NIST, u.s.)
18	Unknown	9.13 (270, 332)	C_20_H_30_O_4_	333.20662	−0.120	Diterpenoid	4 (SIRIUS)
19	Isoginkgetin	9.29 (270, 332)	C_32_H_22_O_10_	565.11334	0.230	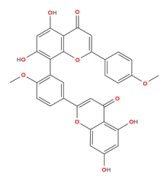	2a (NIST)
20	Biflavonoid-flavone base + 3O and flavone base + 2O + 1MeO	9.43 (270, 332)	C_31_H_20_O_10_	551.09777	0.091	4′-*O*- methylated flavonoids	4 (NIST)
21	Unknown	9.46 (252, 332)	C_32_H_22_O_10_	565.11316	0.589	4′-*O*- methylated flavonoids	4 (SIRIUS)
22	Unknown diterpene dimer	9.52 (276)	C_40_H_52_O_8_	659.35833	0.091	Diterpenoid	4 (SIRIUS)
23	Unknown	9.52 (276)	C_20_H_30_O_3_	317.21185	−0.567	Diterpenoid	4 (SIRIUS)
24	Unknown	9.6	C_20_H_26_O	313.18056	−0.607	Podocarpane diterpenoid	4 (SIRIUS)
25	Unknown	9.6	C_20_H_28_O_3_	315.196	0.063	Abietane diterpenoid	4 (SIRIUS)
26	Pisiferal dimer	9.73 (276, 300, 324)	C_40_H_56_O_4_	599.41004	−0.417	Diterpenoid	4 (SIRIUS)
27	Taxoquinone dimer	9.76	C_40_H_56_O_8_	663.38969	−0.452	Diterpenoid	4 (SIRIUS)
28	Unknown diterpene dimer	9.86 (279, 334)	C_40_H_52_O_8_	659.35809	0.455	Kaurante diterpenoid	4 (SIRIUS)
29	Unknown	9.96 (280, 332)	C_20_H_26_O_3_	315.19542 [M + H]^+^	1.903	Abietane diterpenoid	4 (SIRIUS)
30	Unknown	10.05 (318)	C_22_H_32_O_4_	359.22188	0.974	Diterpenoid	4 (SIRIUS)
31	Unknown	10.15 (278, 315)	C_20_H_28_O_4_	333.20625 [M + H]^+^	0.990	Labdane diterpenoid	4 (SIRIUS)
32	Unknown	10.49 (272)	C_20_H_28_O_2_	301.21628 [M + H]^+^	1.594	Diterpenoid	4 (SIRIUS)
33	Unknown	10.65 (276)	C_20_H_30_O_2_	301.21703	−0.464	Abietane diterpenoid	4 (SIRIUS)
34	Unknown	10.79 (284)	C_20_H_28_O_2_	301.21633 [M + H]^+^	1.428	Abietane diterpenoid	4 (SIRIUS)
35	Unknown	11.18 (318)	C_20_H_28_O_2_	301.21607 [M + H]^+^	2.291	Abietane diterpenoid	4 (SIRIUS)

Notes for Table 4 and Table 5: IHL = in-house library of authentic standards analyzed under identical analytical conditions; u.s. = unresolved structure; NIST = NIST 23 spectral library; MoNA = MassBank of North America; SIRIUS = molecular formula and compound class prediction only (no specific compound annotation); UV = λmax. Level 1, reference standard; Level 2a, private library match; Level 2b, public library match; Level 3, tentative structure; Level 4, confirmed molecular formula only.

## Data Availability

The raw data supporting the conclusions of this article will be made available by the authors on request.

## Supplementary Materials

The following supporting information can be downloaded at: https://www.mdpi.com/article/10.3390/plants15213391/s1, Table S1. MS^2^ fragments of all features reported in Table 4 for *Oenothera rosea*. Figure S1. Annotated mass spectra for all *m*/*z* values of compounds reported in Table 4. Table S2. MS^2^ fragments of all features reported in Table 5 for *Taxodium mucronatum*. Figure S2. Annotated mass spectra for all *m*/*z* values of compounds reported in Table 5. Figure S3. Representative photograph of commercially obtained *Oenothera rosea* (“hierba del golpe”) prior to processing. Figure S4. Representative photograph of *Taxodium mucronatum* collected near Monterrey, Nuevo León, México, showing the aerial tissues used for sample preparation. Plant identification was performed using standard taxonomic keys and floristic references. Figure S5. *Taxodium mucronatum* tree at the collection site in Monterrey, Nuevo León, Mexico.

## Abbreviations

The following abbreviations are used in this manuscript:

ROS	reactive oxygen species
NF-κB,	nuclear factor kappa-light-chain-enhancer of activated B cells
iNOS	inducible nitric oxide synthase
NO	nitric oxide
TPC	total phenolic content
DPPH	2,2-diphenyl-1-picryl-hydrazyl
GAE	gallic acid equivalents
TE	trolox equivalents
ABTS	2,2′-azino-bis (3-ethylbenzothiazoline)-6-sulfonic acid
ESI	electrospray ionization
MS/MS	tandem mass spectrometry
LC-MS	liquid chromatography–mass spectrometry
FA	formic acid
PDA	photo diode array
CID	collision-induced dissociation
CAA	cellular antioxidant activity
HDFa	human dermal fibroblasts
DMEM	Dulbecco’s modified eagle medium
FBS	fetal bovine serum
PBS	phosphate-buffered saline
DCFH-DA	dichlorodihydrofluorescein diacetate
AAPH	20-azobis (2-methylpropionamidine) dihydrochloride
AUC	area under the curve
LPS	lipopolysaccharide
COX	dry weight
dw	cyclooxygenase
AGE’s	advanced glycation end-products
IL-8	interleukin 8
TGF	transforming growth factor
