# Supplementary material for: Phenolic Profile, Antioxidant Activity, and Wound-Healing Potential of Oenothera rosea and Taxodium mucronatum Extracts
Source: Plants (Basel). 2026 Nov 2;15(21):3391. doi: 10.3390/plants15213391 (PMC13765647; doi:10.3390/plants15213391)

Supplementary Material S1:

Table S1. MS² Fragments of all features reported in Table 4 (Found in *Oenothera rosea*)

No	Tentative compound	RT (UV)	Formula	*m/z* [M-H]-	MS² Fragments
1	Gallic acid	1.76 (270)	$C_7H_6O_5$	169.01398	125(100), 169(71), 81(3)
2	Benzoic acid + 2O, *O*-Hex	2.31 (270)	$C_{13}H_{16}O_9$	315.07191	315(100), 152(69), 153(35), 108(35), 109(20)
3	Protocatechuic acid	2.51 (260, 290)	$C_7H_6O_4$	153.01923	109(100), 153(46)
4	Caftaric acid	2.85 (300, 326)	$C_{13}H_{12}O_9$	311.04055	179(100), 149(74), 191(19), 353(16), 135(15), 311(10)
5	NeoChlorogenic acid	2.97 (286, 322)	$C_{16}H_{18}O_9$	353.08700	191(100), 179(70), 135(27), 353(13)
6	Unknown	3.44 (266, 338)	$C_9H_8O_3$	163.03996	119(100), 163(23), 118(7)
7	Unknown	3.88 (266)	$C_{13}H_8O_8$	291.01446	247(100), 291(8)
8	Sulfo jasmonate	4.04 (265)	$C_{12}H_{18}O_7S$	305.06982	305(100), 96(64), 225(47), 59(23)
9	4-*O*-*p*-Coumaroylquinic acid	4.42 (262)	$C_{16}H_{18}O_8$	337.09251	173(100), 163(21)
10	Ethyl gallate	4.81 (265)	$C_9H_{10}O_5$	197.04537	197(100), 169(27), 124(22)
11	Isomyricitrin	5.03 (260, 354)	$C_{21}H_{20}O_{13}$	479.08235	316(100), 479(53), 317(14)
12	2''-*O*-Galloylhyperin	5.33 (262, 354)	$C_{28}H_{24}O_{16}$	615.09838	615(100), 463(77), 300(76), 301(37)
13	Quercetin-3′-*O*-sulfate	5.53 (254, 350)	$C_{15}H_{10}O_{10}S$	380.99119	301(100), 151(13), 178(11)

14	Delphinidin 3-*O*-rutinoside	5.53 (254, 350)	$C_{27}H_{30}O_{16}$	609.14520	609(100), 300(88), 301(26)
15	Isoquercetin/ Hyperoside	5.62 (254, 354)	$C_{21}H_{20}O_{12}$	463.0868	300(100), 463(55), 301(39), 316(8), 271(6)
16	Guaiaverin	6 (254, 350)	$C_{20}H_{18}O_{11}$	433.0769	300(100) 433(34) 301(19) 226(10)
17	Quercitrin	6.28 (255, 350)	$C_{21}H_{20}O_{11}$	447.09223	300(100), 301(76), 447(60)
18	Quercetin 3-*O*-glucosyl-6”-acetate	6.6 (268, 354)	$C_{23}H_{22}O_{13}$	505.09798	300(100), 505(47), 301(23), 271(9), 463(4)
19	Afzelin	6.9 (265, 356)	$C_{21}H_{20}O_{10}$	431.09749	285(100), 284(78), 431(52), 255(13), 227(6)
20	Kaempferol 7-(6"-*p*-succinylglucoside)	7.22 (265, 356)	$C_{25}H_{24}O_{14}$	547.10863	300(100), 547(48), 301(16), 505(11), 271(11)
21	Quercetin 3-(3,6”-diacetylgalactoside)	7.65 (268, 356)	$C_{25}H_{24}O_{14}$	547.10876	300(100), 547(78), 301(25), 505(9)
22	Quercetin 3-(2",3",4"-triacetylgalactoside)	8.37 (265, 354)	$C_{27}H_{26}O_{15}$	589.11867	300(100), 589(44), 301(12), 271(11), 547(8)

Figure S1. Annotated Mass Spectra for all *m/z* values reported in Table 4

(1) *Oenothera rosea* - 5mg/mL – NEG @1.76min MS2 - CE: 30.0 c - precursor *m/z*: 169.0140, base peak *m/z*: 125.0243 (2.1E7) Scan definition: FTMS – c ESI d Full ms2

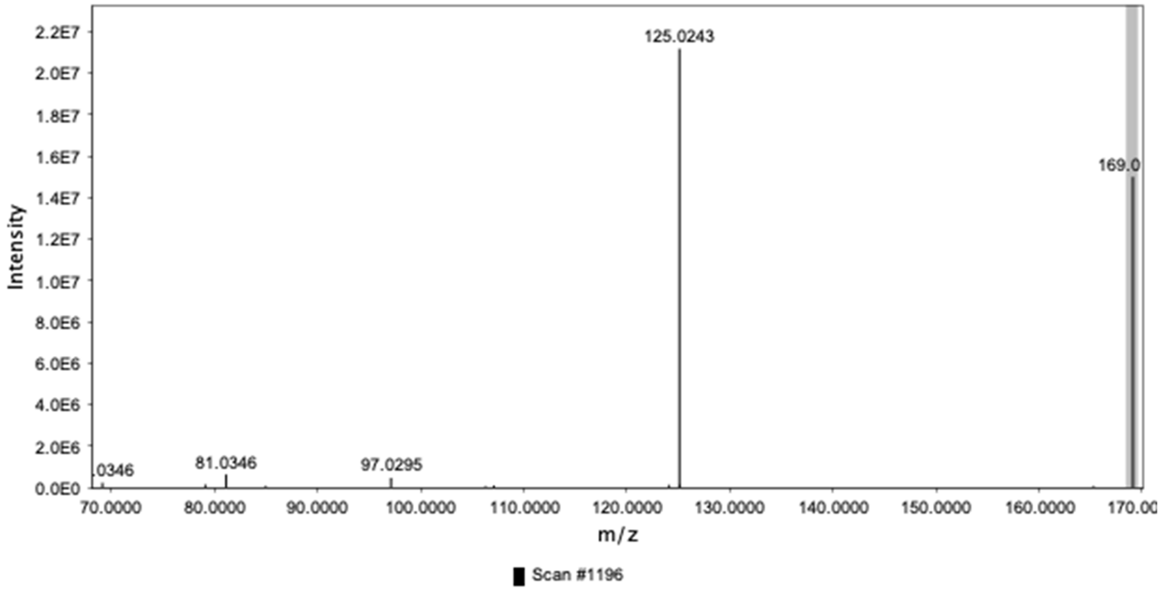

(2) *Oenothera rosea* - 5mg/mL – NEG @2.31min MS2 - CE: 30.0 c - precursor *m/z*: 315.0719, base peak *m/z*: 315.0718 (1.4E6) Scan definition: FTMS – c ESI d Full ms2

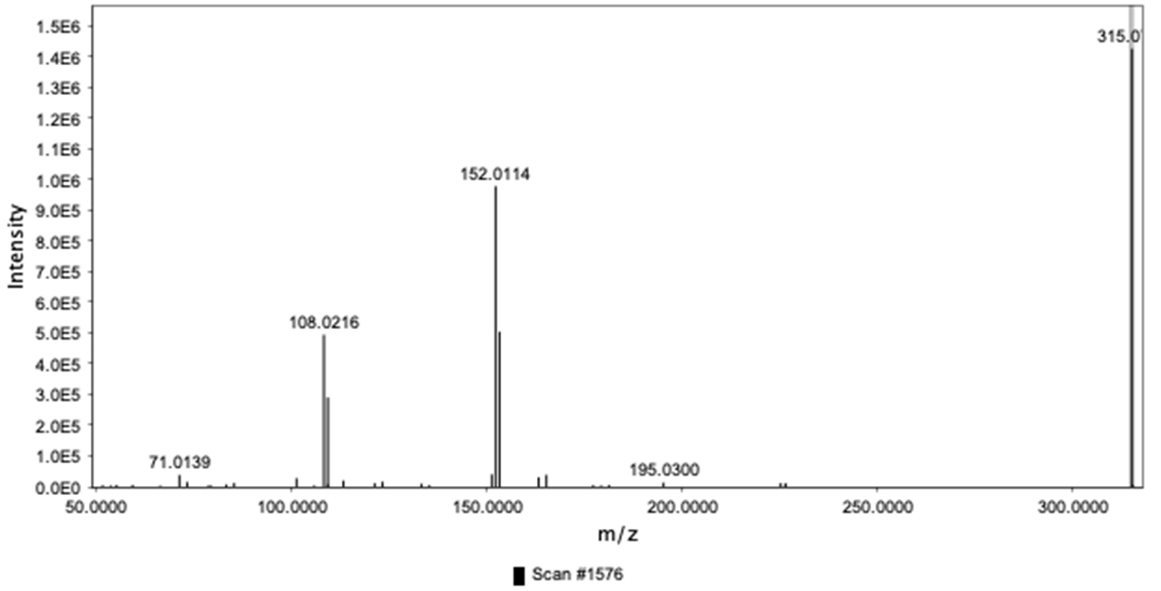

(3) *Oenothera rosea* - 5mg/mL – NEG @2.51min MS2 - CE: 30.0 c - precursor *m/z*: 153.0193, base peak *m/z*: 109.0294 (1.6E6) Scan definition: FTMS – c ESI d Full ms2

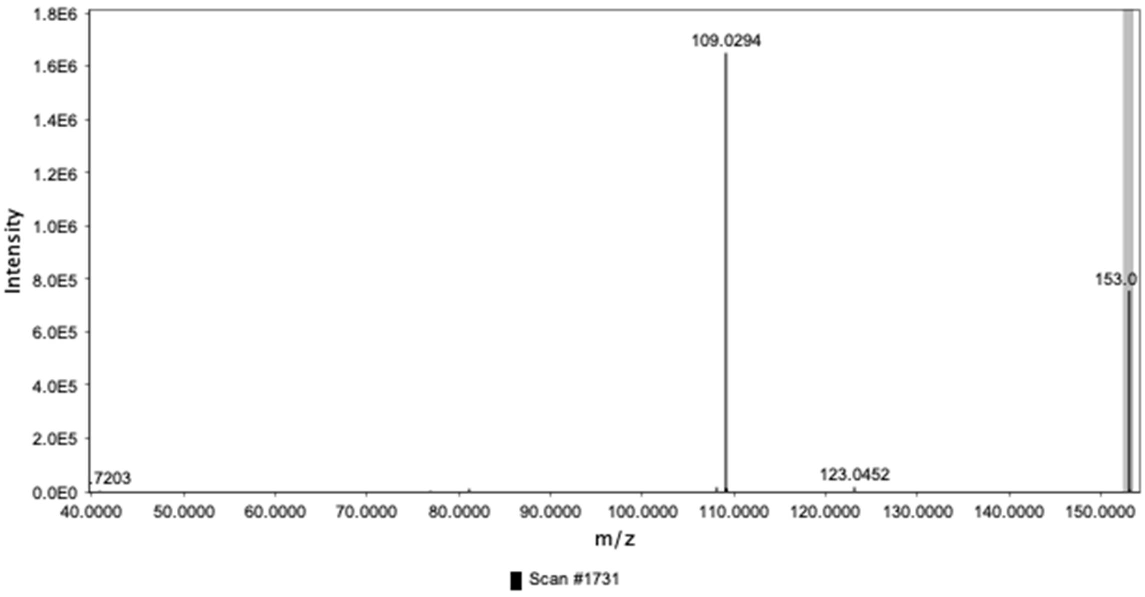

(4) *Oenothera rosea* - 5mg/mL – NEG @2.85 min MS2 - CE: 30.0 c - precursor *m/z*: 311.04055, base peak *m/z*: 179.0349 (2.0E5) Scan definition: FTMS – c ESI d Full ms2

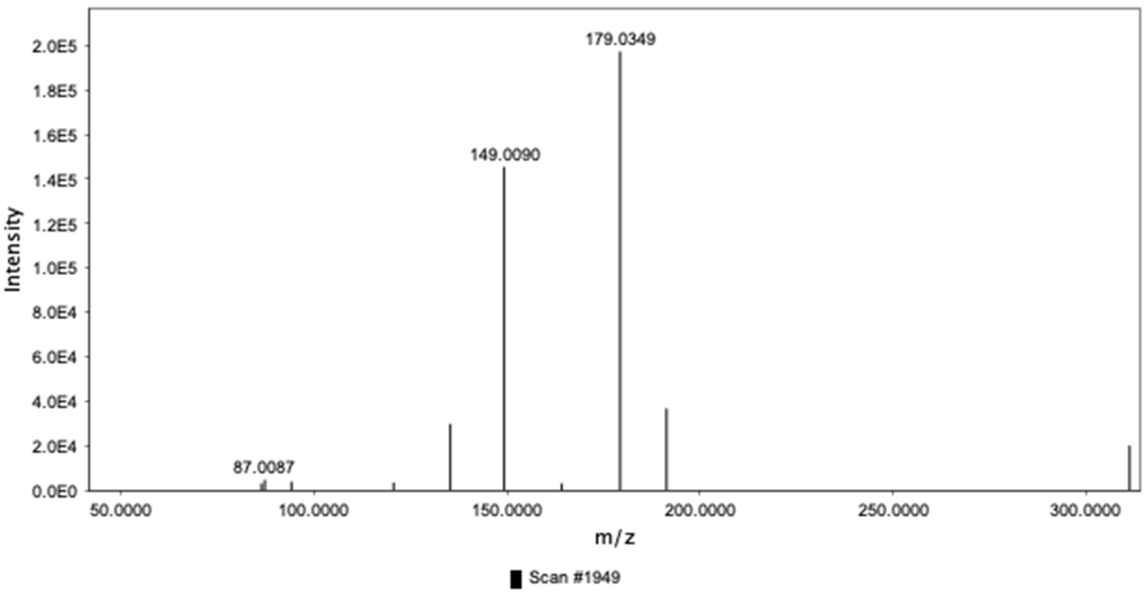

(5) *Oenothera rosea* - 5mg/mL – NEG @2.97 min MS2 - CE: 30.0 c - precursor *m/z*: 353.08700, base peak *m/z*: 191.0559 (2.6E6) Scan definition: FTMS – c ESI d Full ms2

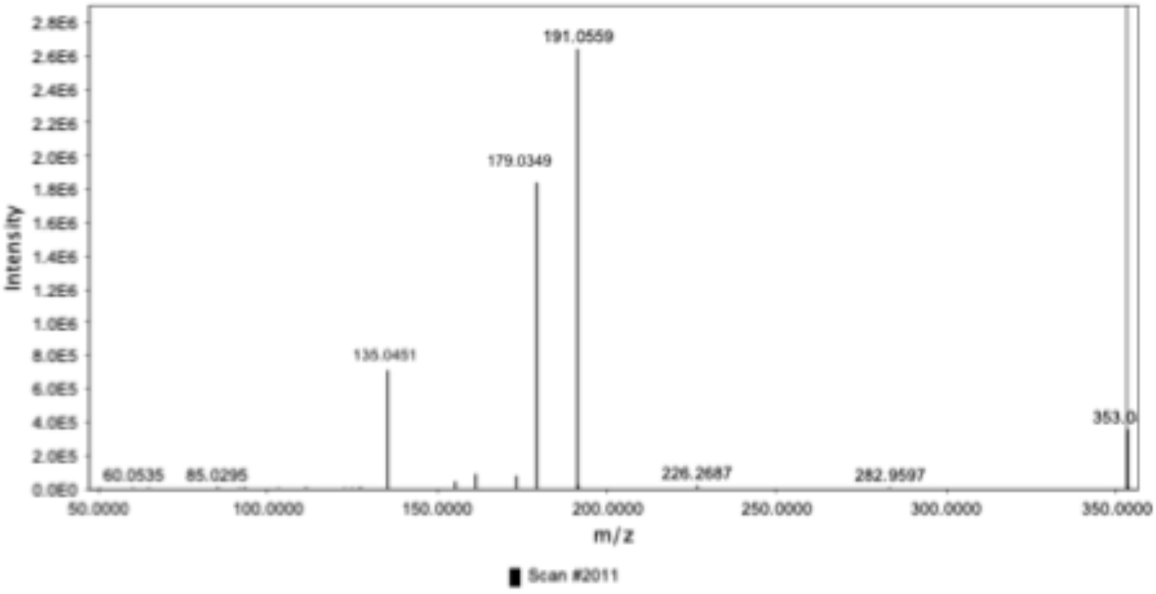

(6) *Oenothera rosea* - 5mg/mL – NEG @3.44 min MS2 - CE: 30.0 c - precursor *m/z*: 163.03996, base peak *m/z*: 119.0502 (2.7E5) Scan definition: FTMS – c ESI d Full ms2

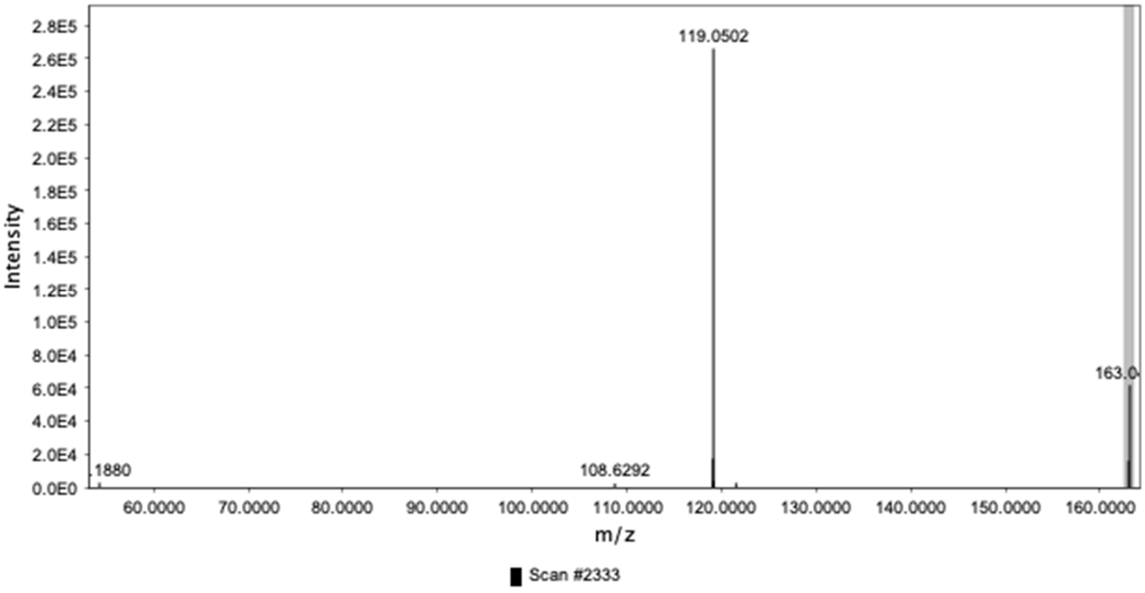

(7) *Oenothera rosea* - 5mg/mL – NEG @3.88 min MS2 - CE: 30.0 c - precursor *m/z*: 291.01446, base peak *m/z*: 247.0246 (2.9E6) Scan definition: FTMS – c ESI d Full ms2

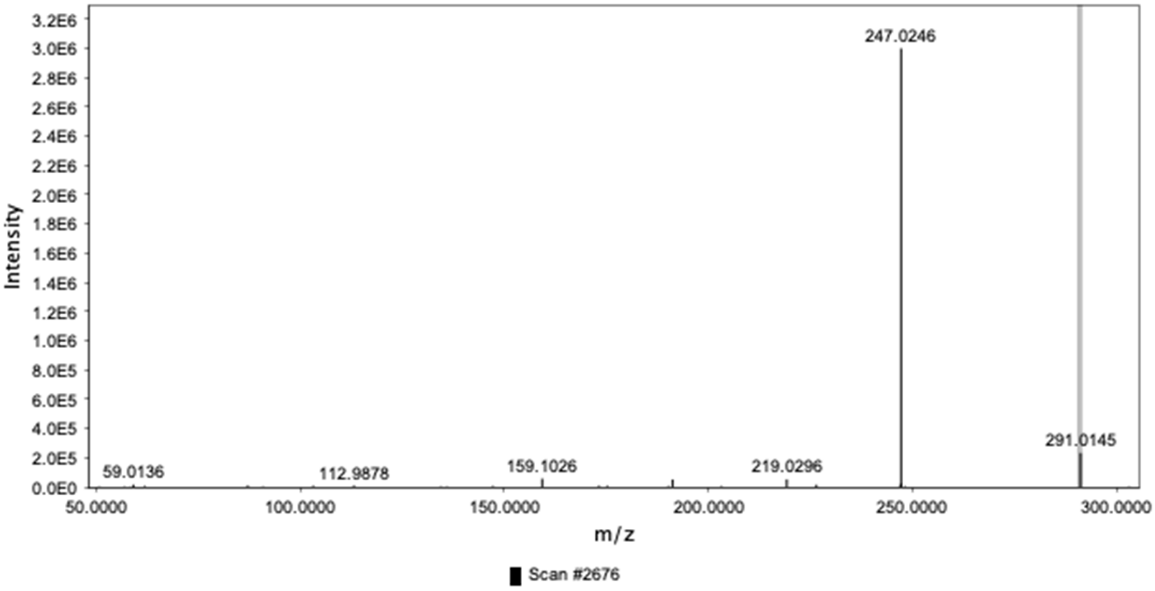

(8) *Oenothera rosea* - 5mg/mL – NEG @4.04 min MS2 - CE: 30.0 c - precursor *m/z*: 305.06982, base peak *m/z*: 305.0698 (1.0E6) Scan definition: FTMS – c ESI d Full ms2

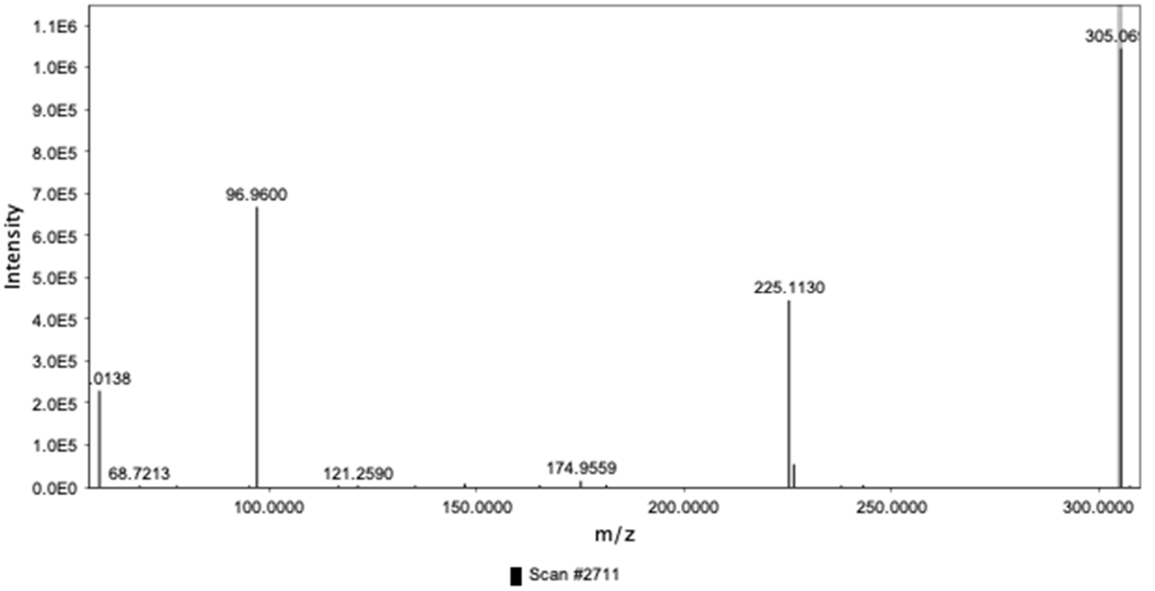

(9) *Oenothera rosea* - 5mg/mL – NEG @4.42 min MS2 - CE: 30.0 c - precursor *m/z*: 337.09251, base peak *m/z*: 173.0453 (2.5E6) Scan definition: FTMS – c ESI d Full ms2

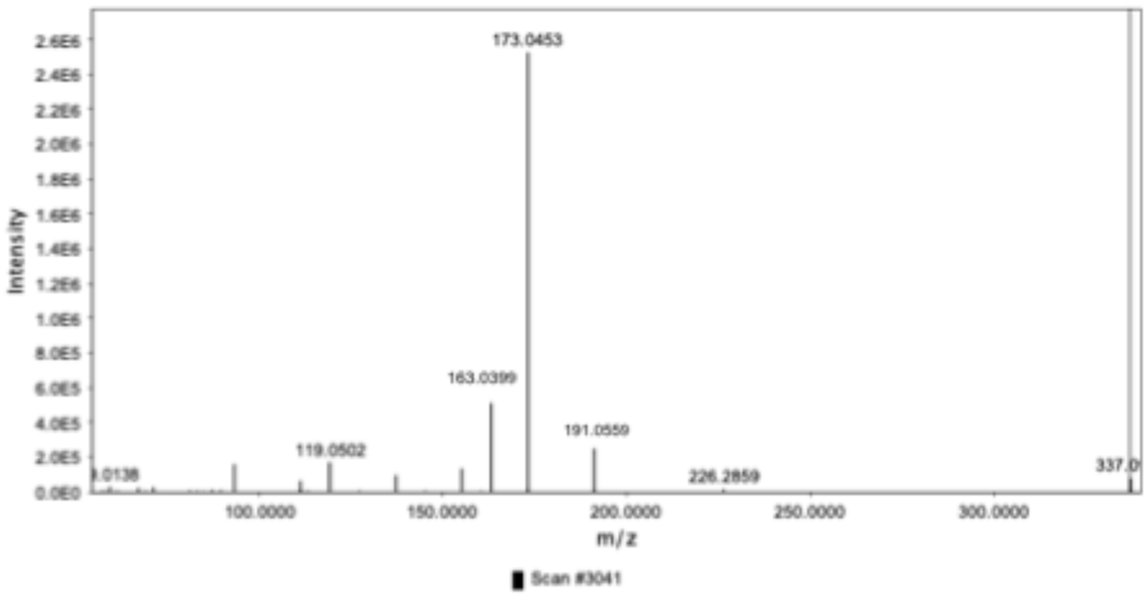

(10) *Oenothera rosea* - 5mg/mL – NEG @4.81 min MS2 - CE: 30.0 c - precursor *m/z*: 197.04537, base peak *m/z*: 197.04537 (6.1E5) Scan definition: FTMS – c ESI d Full ms2

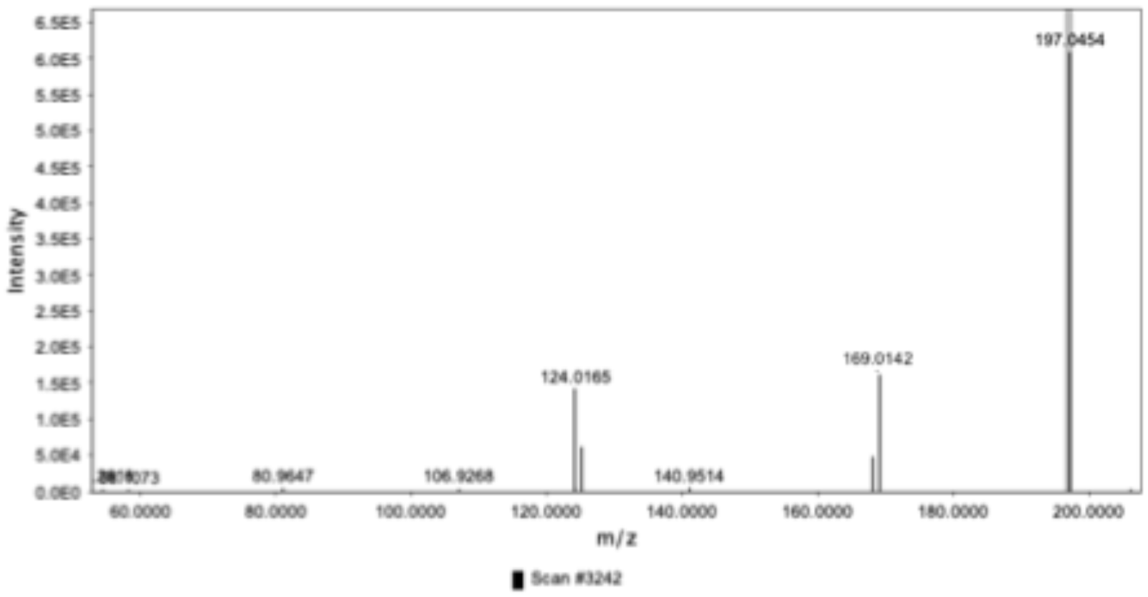

(11) Oenothera rosea - 5mg/mL – NEG @5.03 min MS2 - CE: 30.0 c - precursor *m/z*: 479.08235, base peak *m/z*: 316.0220 (5.9E6) Scan definition: FTMS – c ESI d Full ms2

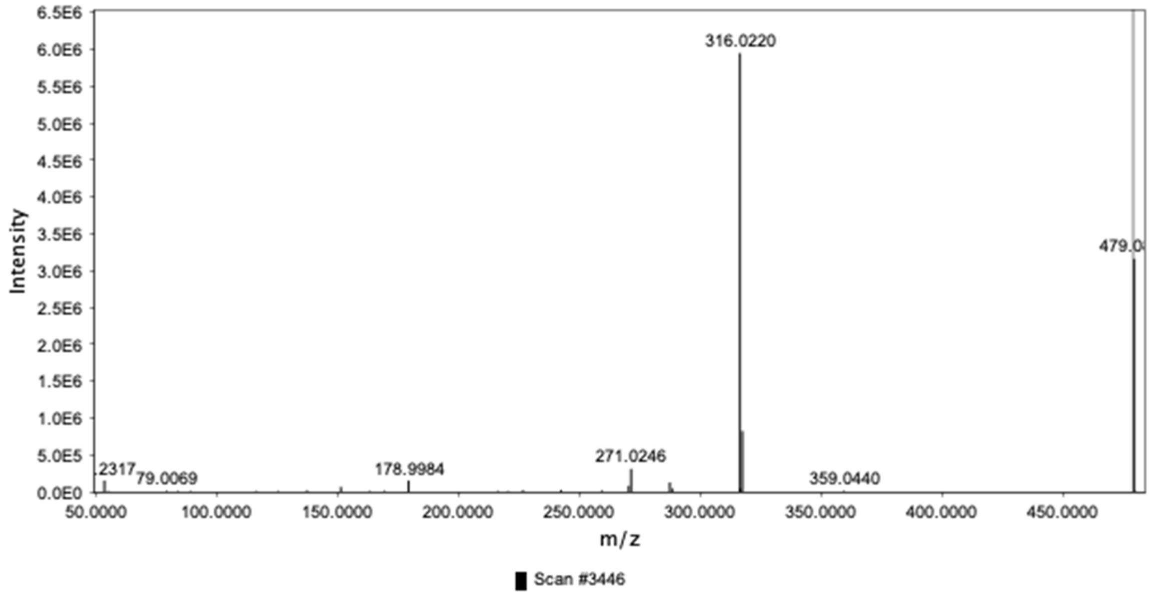

(12) Oenothera rosea - 5mg/mL – NEG @5.33 min MS2 - CE: 30.0 c - precursor *m/z*: 615.09838, base peak *m/z*: 615.09838 (1.5E6) Scan definition: FTMS – c ESI d Full ms2

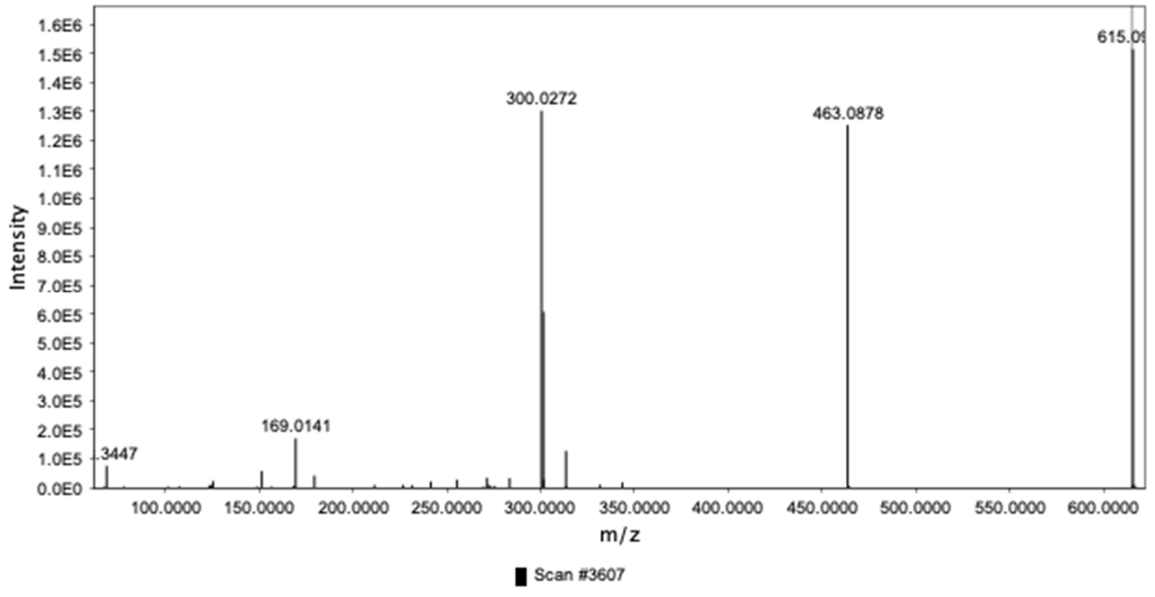

(13) Oenothera rosea - 5mg/mL – NEG @5.53 min MS2 - CE: 30.0 c - precursor *m/z*: 380.99119, base peak *m/z*: 301.0350 (2.3E6) Scan definition: FTMS – c ESI d Full ms2

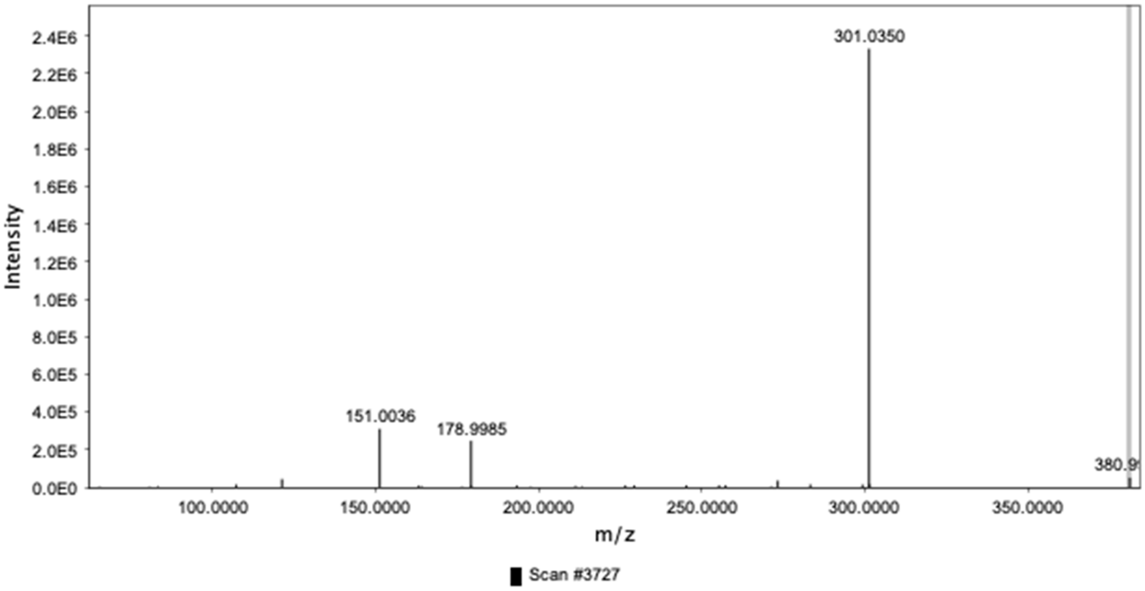

(14) Oenothera rosea - 5mg/mL – NEG @5.53 min MS2 - CE: 30.0 c - precursor *m/z*: 609.14520, base peak *m/z*: 609.14520 (8.0E6) Scan definition: FTMS – c ESI d Full ms2

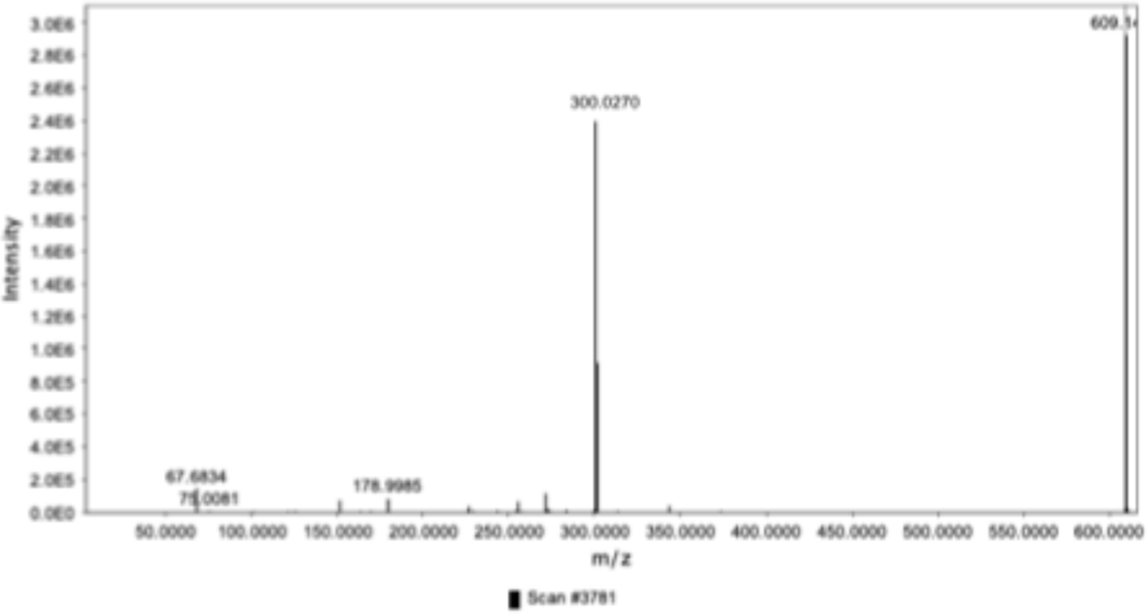

(15) Oenothera rosea - 5mg/mL – NEG @5.62 min MS2 - CE: 30.0 c - precursor *m/z*: 463.0868, base peak *m/z*: 300.0270 (1.9E7) Scan definition: FTMS – c ESI d Full ms2

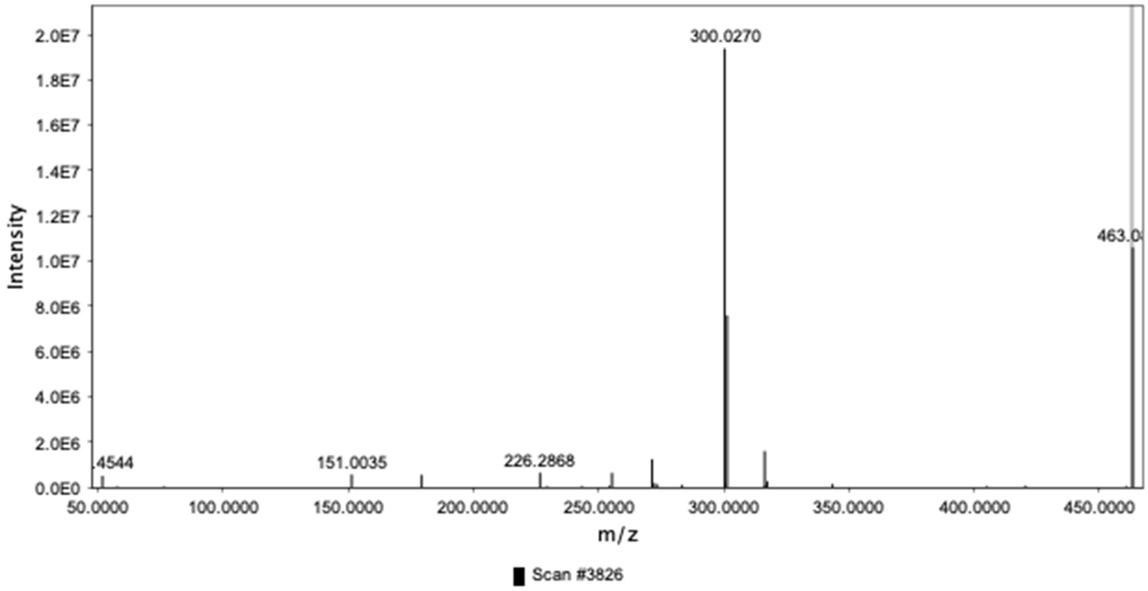

(16) Oenothera rosea - 5mg/mL – NEG @6.00 min MS2 - CE: 30.0 c - precursor *m/z*: 433.07690, base peak *m/z*: 300.02716 (3.9E5) Scan definition: FTMS – c ESI d Full ms2

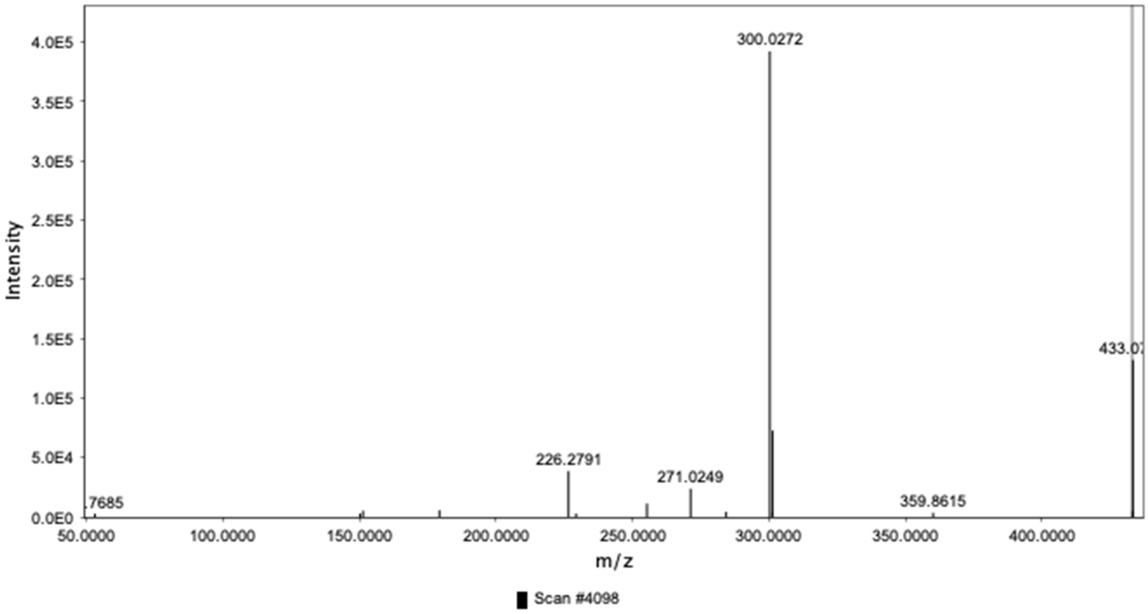

(17) Oenothera rosea - 5mg/mL – NEG @6.28 min MS2 - CE: 30.0 c - precursor *m/z*: 447.09223, base peak *m/z*: 300.0271 (1.0E7) Scan definition: FTMS – c ESI d Full ms2

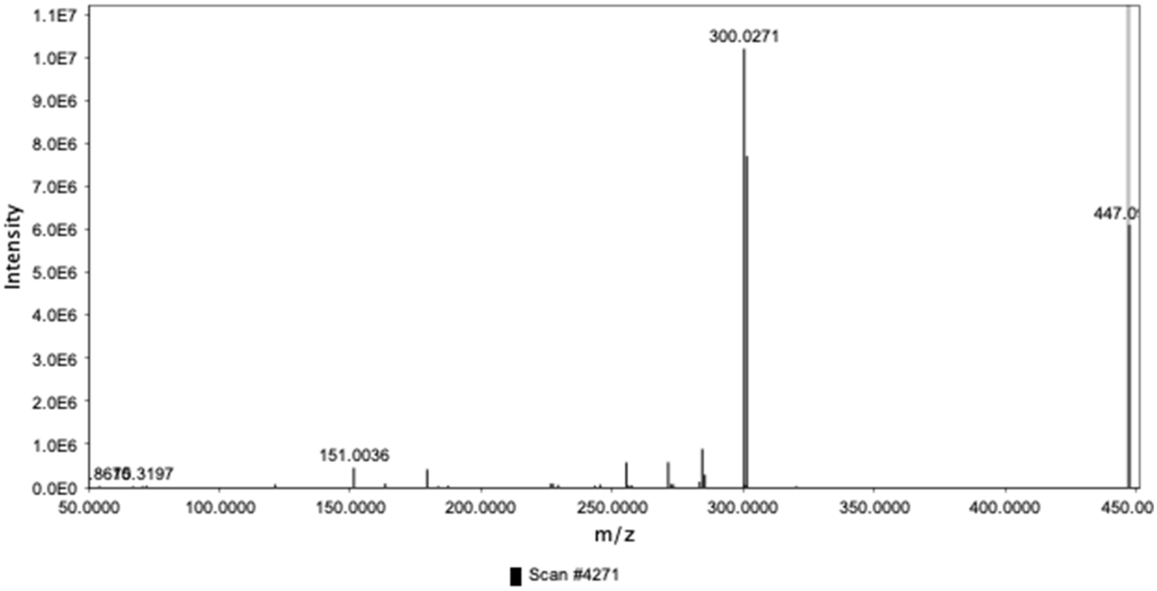

(18) Oenothera rosea - 5mg/mL – NEG @6.6 min MS2 - CE: 30.0 c - precursor *m/z*: 505.09798, base peak *m/z*: 300.0270 (4.0E6) Scan definition: FTMS – c ESI d Full ms2

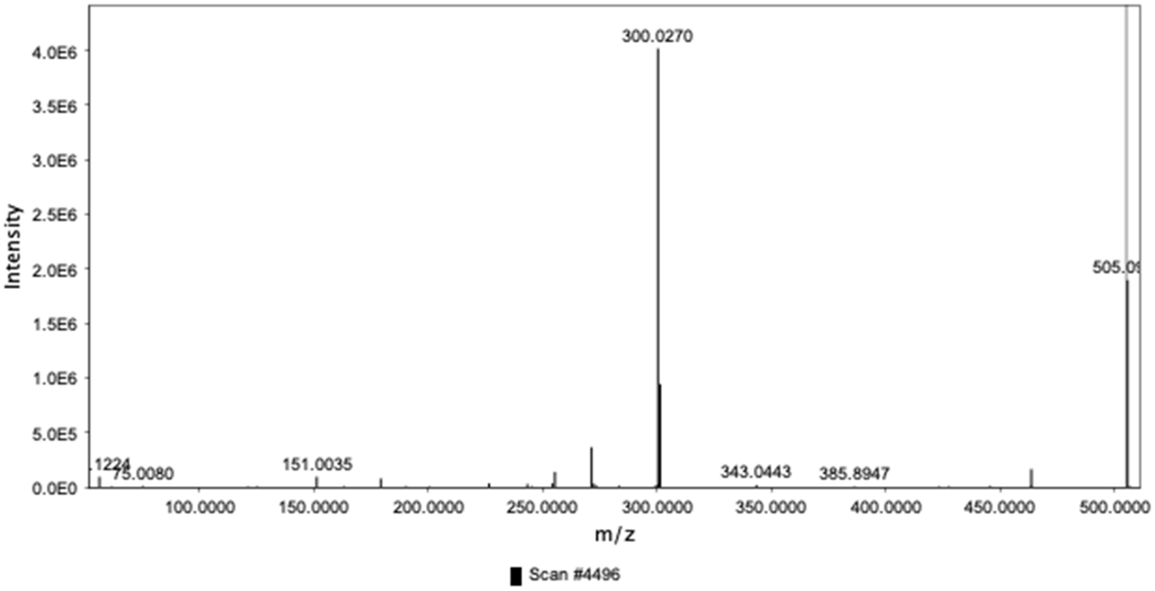

(19) Oenothera rosea - 5mg/mL – NEG @6.9 min MS2 - CE: 30.0 c - precursor *m/z*: 431.09749, base peak *m/z*: 285.0402 (6.5E5) Scan definition: FTMS – c ESI d Full ms2

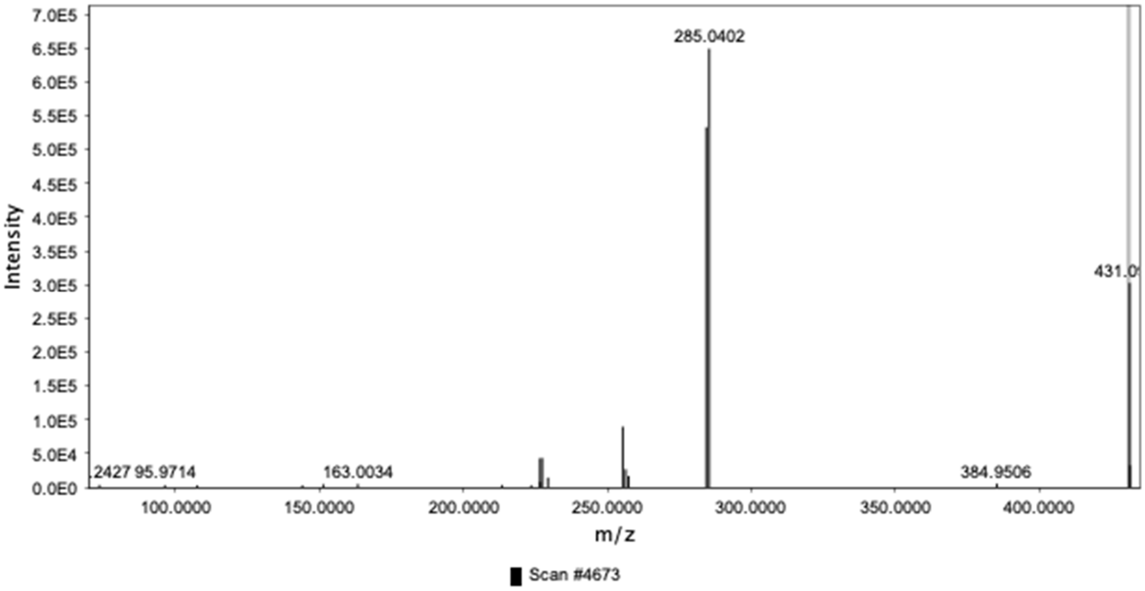

(20) Oenothera rosea - 5mg/mL – NEG @7.22 min MS2 - CE: 30.0 c - precursor *m/z*: 547.10863, base peak *m/z*: 300.0272 (2.4E6) Scan definition: FTMS – c ESI d Full ms2

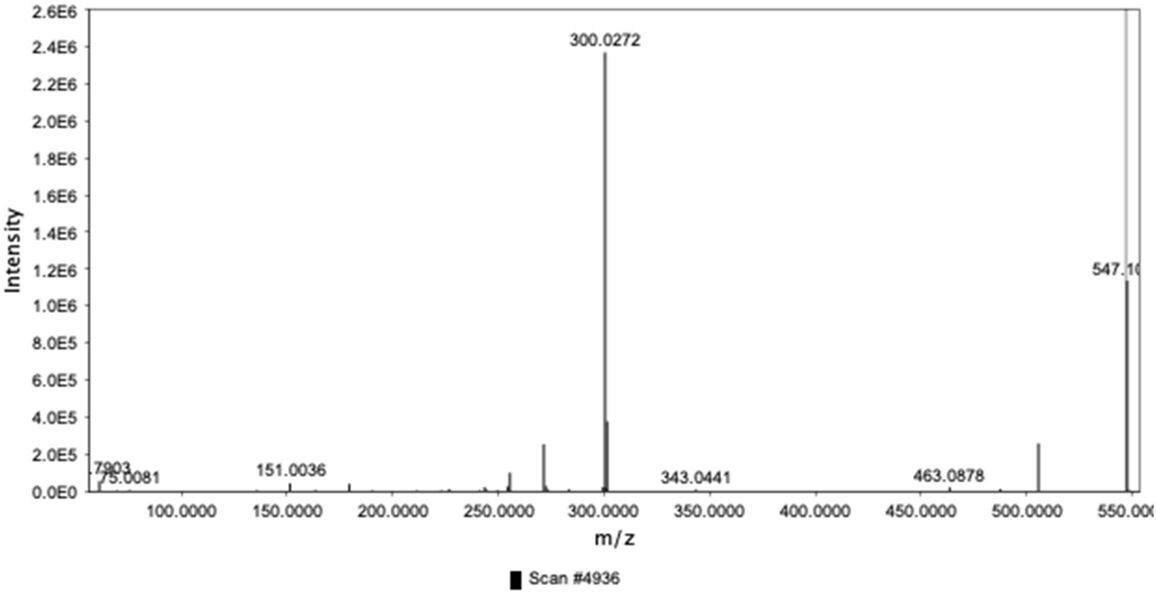

(21) Oenothera rosea - 5mg/mL – NEG @7.65 min MS2 - CE: 30.0 c - precursor *m/z*: 547.10876, base peak *m/z*: 300.0273 (6.6E6) Scan definition: FTMS – c ESI d Full ms2

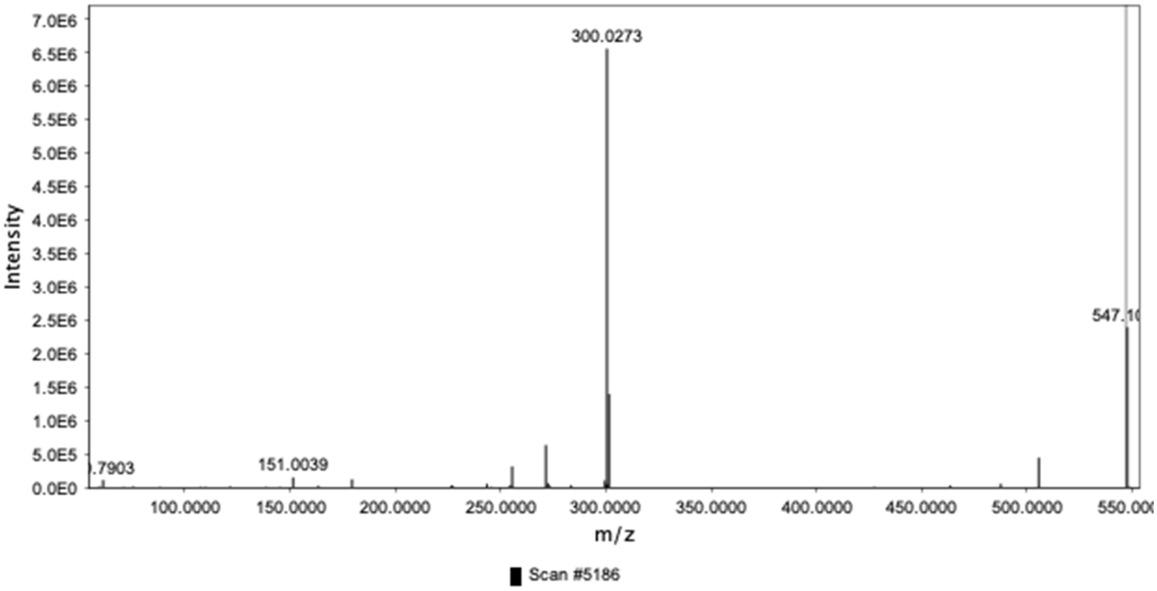

(22) Oenothera rosea - 5mg/mL – NEG @8.37 min MS2 - CE: 30.0 c - precursor *m/z*: 589.11867, base peak *m/z*: 300.0271 (2.3E7) Scan definition: FTMS – c ESI d Full ms2

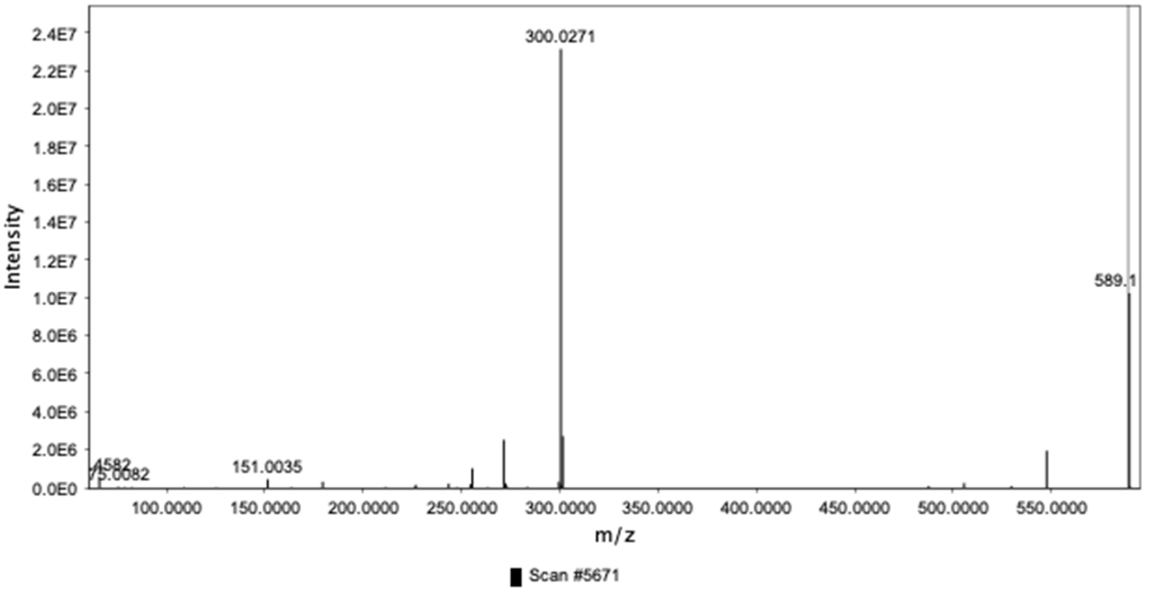

Table S2. MS^2 Fragments of all features reported in Table 5 (Found in *Taxodium mucronatum*)

No	Tentative compound	RT (UV)	Formula	*m/z* [M-H]-	MS^2 Fragments
1	Procyanidin B	3.38 (280, 320)	$C_{30}H_{26}O_{12}$	577.1342	289(100), 407(98), 125(76), 161(23), 425(23), 245(21)
2	Epicatechin	3.64 (280, 320)	$C_{15}H_{14}O_6$	289.07132	289(100), 245(47), 125(22), 109(22), 203(22), 205 (16)
3	Procyanidin B	4.07 (280,306)	$C_{30}H_{26}O_{12}$	577.13445	407(100), 289(97), 125(82), 425(26), 161(23), 245(20), 137(14), 451(13), 577(10)
4	Catechin	4.33 (278)	$C_{15}H_{14}O_6$	289.07142	289(100), 245(52), 203(28), 125(26), 205(22), 179(16)
5	Unknown	4.45 (266, 346)	$C_{23}H_{28}O_9$	447.1655	225(100), 285(94), 224(38), 119(11), 164(4)
6	Unknown	4.81 (280,306)	$C_{25}H_{34}O_{12}$	525.19713	327(100), 161(68), 167(58), 89(33), 59(26), 179(22), 221(17), 71(15)
7	Quercetin-3-*O*-vicianoside	5.3 (278, 344)	$C_{26}H_{28}O_{16}$	595.1298	595(100), 300(91), 301(44)
8	Isoquercetin/ Hyperoside	5.63 (262, 354)	$C_{21}H_{20}O_{12}$	463.08716	300(100), 463(52), 301(39)
9	Reynoutrin	6.04 (260, 360)	$C_{20}H_{18}O_{11}$	433.07676	300(100), 433(41), 301(19)
10	Quercitrin	6.27 (260, 348)	$C_{21}H_{20}O_{11}$	447.09206	300(100), 301(77), 447(52)
11	Kaempferol-3-*O*-rhamnoside	6.91 (278, 344)	$C_{21}H_{20}O_{10}$	431.09765	285(100), 284(83), 431(55), 255(16)
12	Unknown	7.04 (270, 340)	$C_{22}H_{22}O_{11}$	461.10827	461(100), 314(91), 315(34), 299(31), 271(14), 300(10), 285(10)
13	Quercetin	7.68 (265, 340)	$C_{15}H_{10}O_7$	301.03523	301(100), 151(54), 178(28)
14	Naringenin	8.21 (278)	$C_{15}H_{12}O_5$	271.06105	271(100), 151(80), 119(34), 107(12), 177(11), 93(10)

15	Cupressuflavone	8.66 (274, 330)	$C_{30}H_{18}O_{10}$	537.08206	537(100), 375(83), 417(27), 443(13), 399(12), 331(8)
16	Unknown	8.87 (275, 340)	$C_{32}H_{24}O_{12}$	347.18564	347(100), 303(62), 259(32), 299(14)
17	Hinokiflavone	9.04 (270, 336)	$C_{30}H_{18}O_{10}$	537.08179	537(100), 284(4)
18	Unknown	9.13 (270, 332)	$C_{20}H_{30}O_4$	333.20662	315(100), 333(14.29)
19	Isoginkgetin	9.29 (270, 332)	$C_{31}H_{22}O_{10}$	565.11334	533(100), 565(84), 389(35), 374(14)
20	Biflavonoid-flavone base + 3O and flavone base +2O + 1MeO	9.43 (270, 332)	$C_{31}H_{20}O_{10}$	551.09777	551(100), 536(17), 283(6)
21	Unknown	9.46 (252, 332)	$C_{32}H_{22}O_{10}$	565.11316	565(100), 389(78), 533(12)
22	Unknown diterpene dimer	9.52 (276)	$C_{40}H_{52}O_8$	659.35833 [M-H]-	329(100), 226(1)
23	Unknown	9.52 (276)	$C_{20}H_{30}O_3$	317.21185 [M-H]-	317(100), 299(98), 226(5)
24	Unknown	9.6	$C_{20}H_{26}O$	313.18056 [M-H]-	313(100), 226(1)
25	Unknown	9.6	$C_{20}H_{28}O_3$	315.196 [M+H]+	315(100), 297(1), 271(1)
26	Pisiferal dimer	9.73 (276, 300, 324)	$C_{40}H_{56}O_4$	599.41004 [M-H]-	299(100), 226(1)
27	Taxoquinone dimer	9.76	$C_{40}H_{56}O_8$	663.38969 [M-H]-	331(100), 298(6), 313(2)
28	Unknown diterpene dimer	9.86 (279, 334)	$C_{40}H_{52}O_8$	659.35809 [M-H]-	329(100), 314(58), 226(2)
29	Unknown	9.96 (280, 332)	$C_{20}H_{26}O_3$	315.19542 [M+H]+	245(100), 315(59), 83(46), 297(33), 55(24), 255(15)
30	Unknown	10.05 (318)	$C_{22}H_{32}O_4$	359.22188 [M-H]-	298(100), 313(51), 359(22), 315(13), 285(12)

31	Unknown	10.15 (278, 315)	$C_{20}H_{28}O_4$	333.20625 [M+H]+	269(100), 297(57), 255(49), 315(37), 316(37), 333(26), 241(18), 219(15)
32	Unknown	10.49 (272)	$C_{20}H_{28}O_2$	301.21628 [M+H]+	205(100), 217(77), 301(56), 163(49), 245(32), 219(28), 165(23), 231(23), 191(18)
33	Unknown	10.65 (276)	$C_{20}H_{30}O_2$	301.21703 [M+H]+	301(100), 226(2), 300(1)
34	Unknown	10.79 (284)	$C_{20}H_{28}O_2$	301.21633 [M+H]+	205(100), 217(87), 259(77), 301(71), 163(39), 245(31), 177(24), 189(22), 191(20), 241(19), 203(13), 283(13)
35	Unknown	11.18 (318)	$C_{20}H_{28}O_2$	301.21607 [M+H]+	205(100), 217(87), 301(32), 245(31), 191(16), 231(10)

Figure S2. Annotated Mass Spectra for all *m/z* values reported in Table 5

(1) *Taxodium mucronatum* - 5mg/mL – NEG @3.38 min MS2 - CE: 30.0 c - precursor *m/z*: 577.1342, base peak *m/z*: 289.0715 (2.7E6) Scan definition: FTMS – c ESI d Full ms2

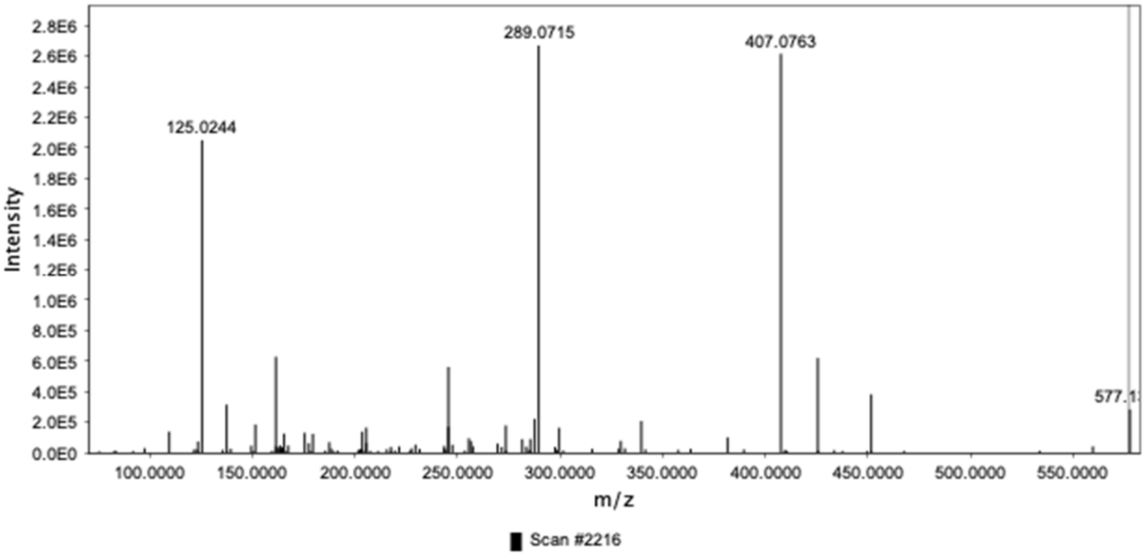

(2) *Taxodium mucronatum* - 5mg/mL – NEG @3.64 min MS2 - CE: 30.0 c - precursor *m/z*: 289.07132, base peak *m/z*: 289.07132 (1.1E7) Scan definition: FTMS – c ESI d Full ms2

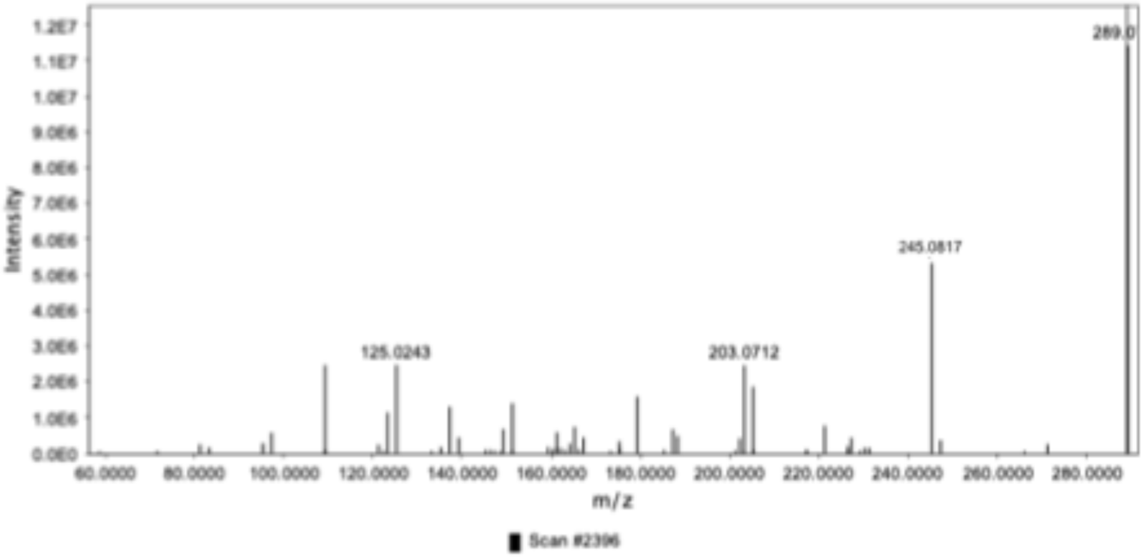

(3) *Taxodium mucronatum* - 5mg/mL – NEG @4.07 min MS2 - CE: 30.0 c - precursor *m/z*: 577.13445, base peak *m/z*: 407.0763 (5.5E5) Scan definition: FTMS – c ESI d Full ms2

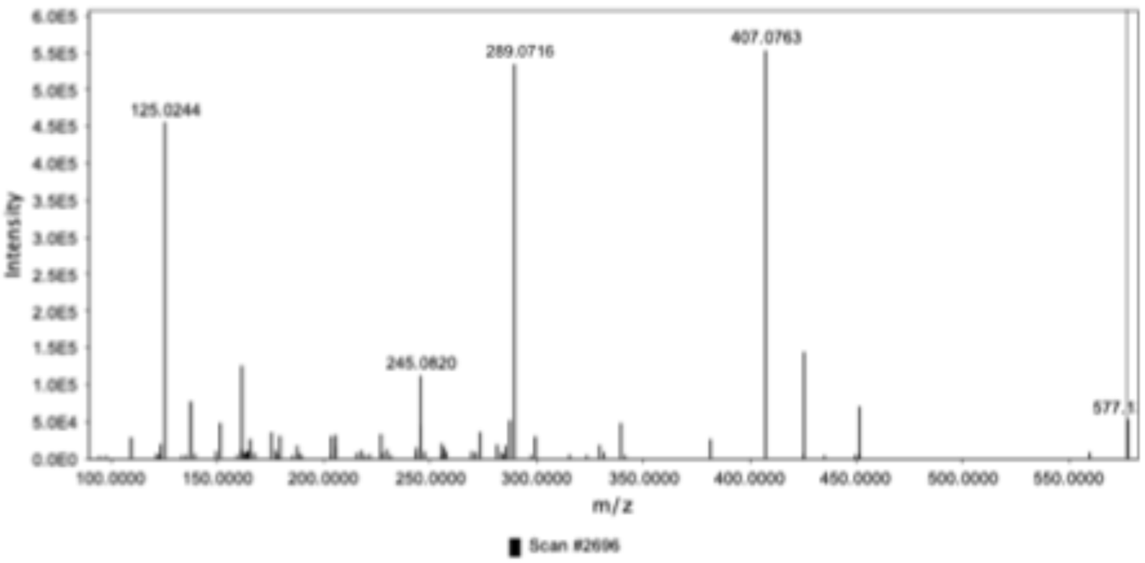

(4) *Taxodium mucronatum* - 5mg/mL – NEG @4.33 min MS2 - CE: 30.0 c - precursor *m/z*: 289.07142, base peak *m/z*: 289.07142 (4.5E6) Scan definition: FTMS – c ESI d Full ms2

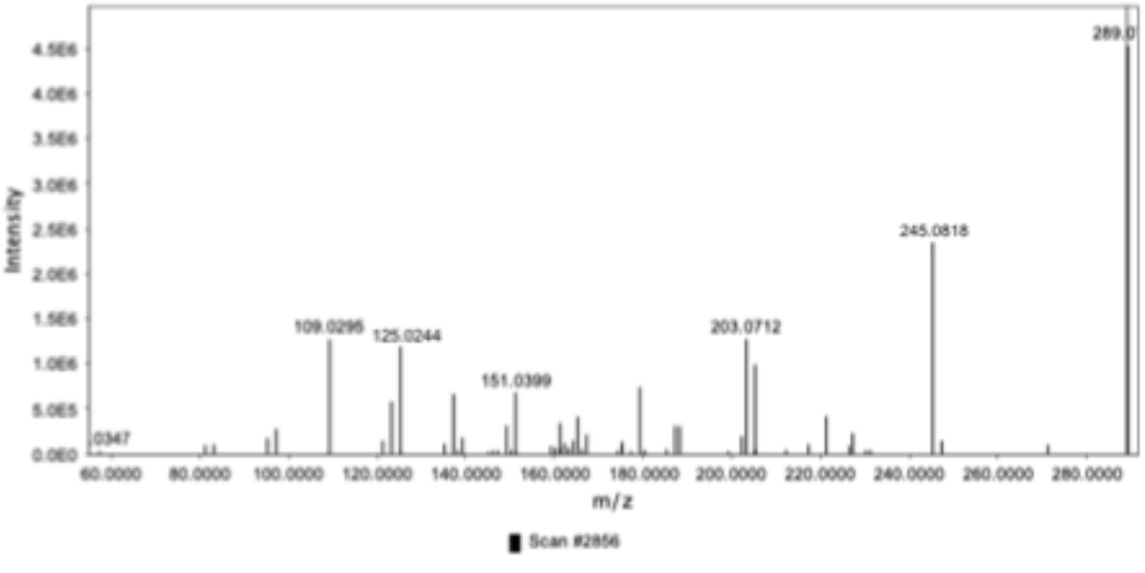

(5) Taxodium mucronatum - 5mg/mL – NEG @4.45 min MS2 - CE: 30.0 c - precursor *m/z*: 447.1655, base peak *m/z*: 225.0920 (5.7E5) Scan definition: FTMS – c ESI d Full ms2

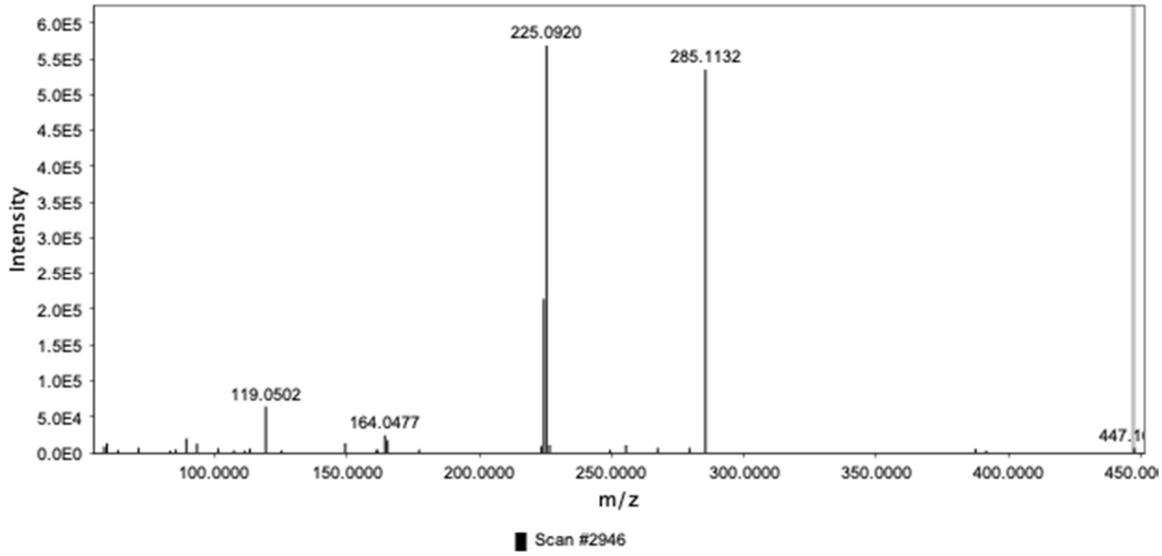

(6) Taxodium mucronatum - 5mg/mL – NEG @4.81 min MS2 - CE: 30.0 c - precursor *m/z*: 525.19713, base peak *m/z*: 327.1234 (6.8E5) Scan definition: FTMS – c ESI d Full ms2

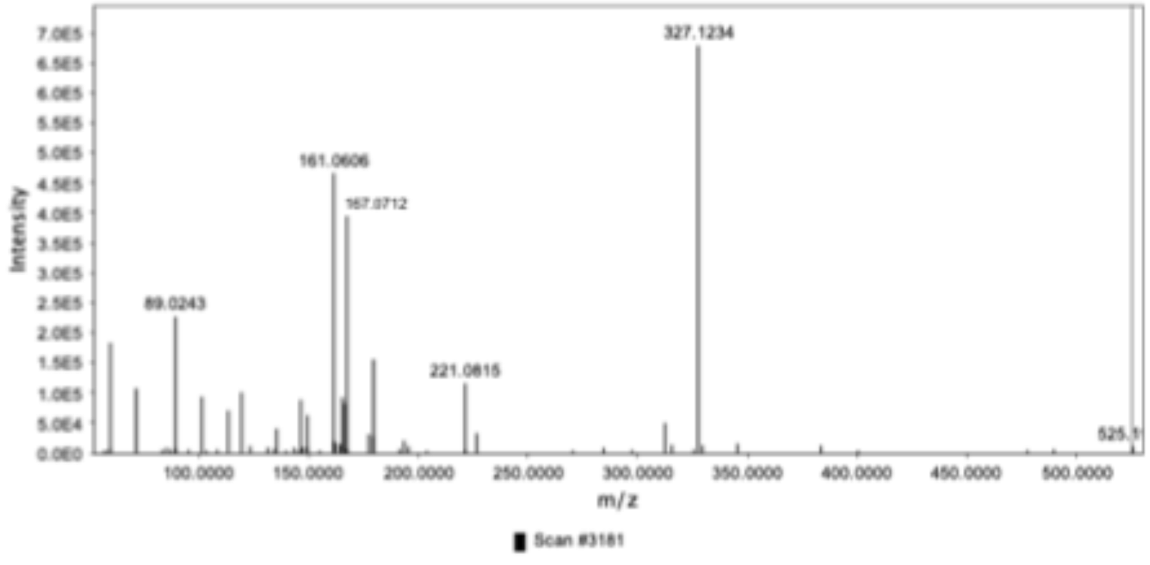

(7) *Taxodium mucronatum* - 5mg/mL – NEG @5.3 min MS2 - CE: 30.0 c - precursor *m/z*: 595.1298, base peak *m/z*: 595.1298 (7.0E5) Scan definition: FTMS – c ESI d Full ms2

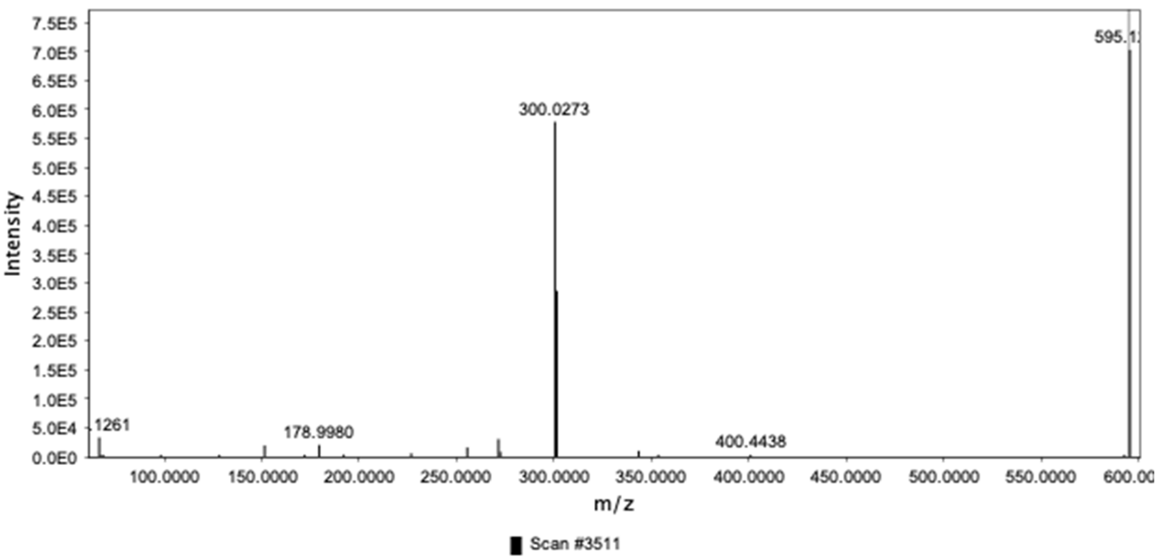

(8) *Taxodium mucronatum* - 5mg/mL – NEG @5.63 min MS2 - CE: 30.0 c - precursor *m/z*: 463.08716, base peak *m/z*: 300.0270 (7.4E6) Scan definition: FTMS – c ESI d Full ms2

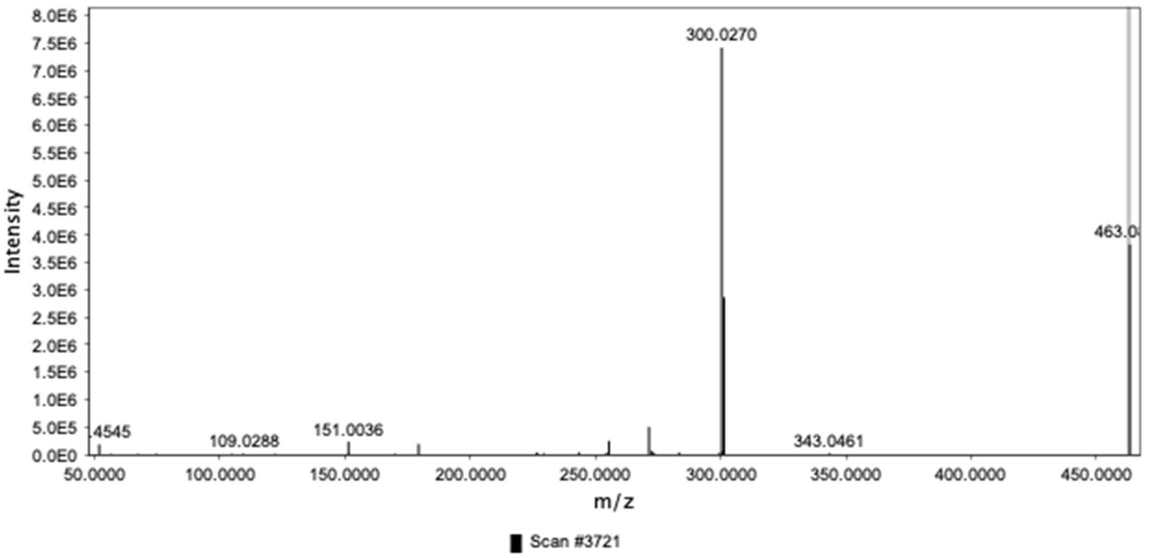

(9) *Taxodium mucronatum* - 5mg/mL – NEG @6.04 min MS2 - CE: 30.0 c - precursor *m/z*: 433.0769, base peak *m/z*: 300.0271 (3.1E6) Scan definition: FTMS – c ESI d Full ms2

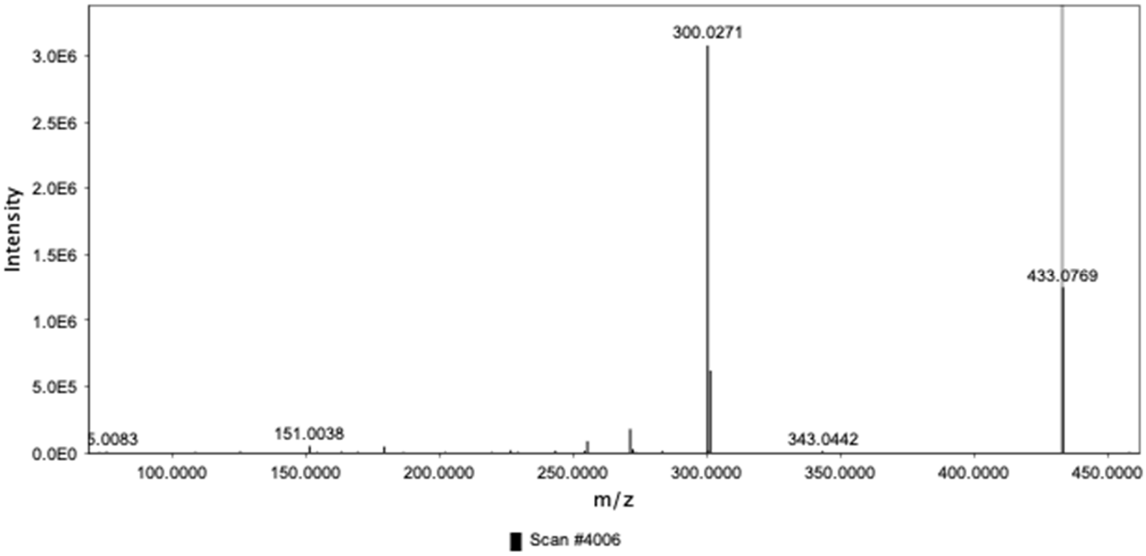

(10) *Taxodium mucronatum* - 5mg/mL – NEG @6.27 min MS2 - CE: 30.0 c - precursor *m/z*: 447.09206, base peak *m/z*: 300.0273 (8.5E6) Scan definition: FTMS – c ESI d Full ms2

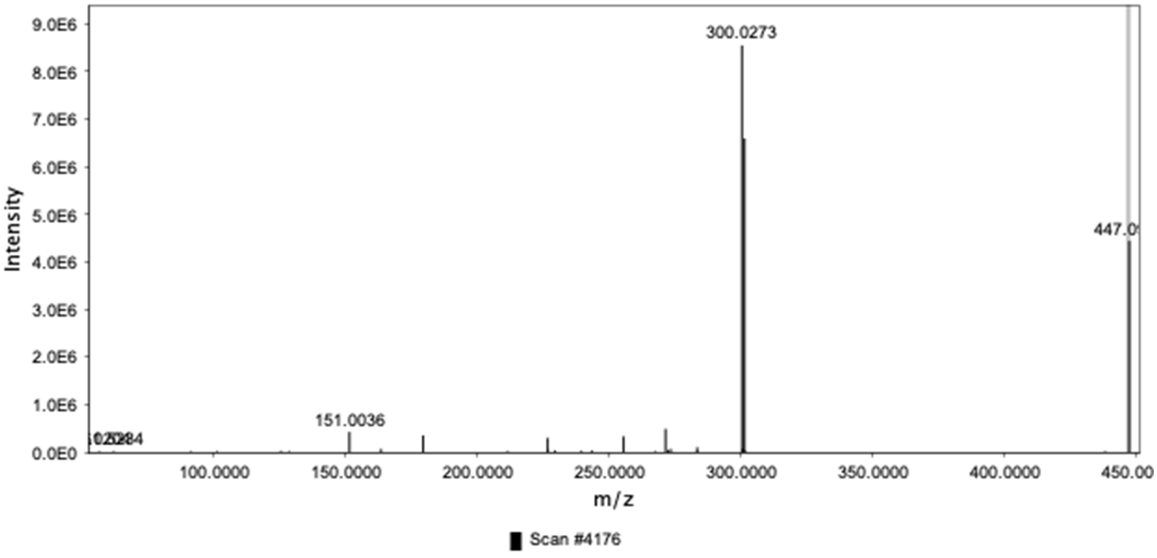

(11) Taxodium mucronatum - 5mg/mL – NEG @6.91 min MS2 - CE: 30.0 c - precursor *m/z*: 431.09765, base peak *m/z*: 285.0402 (4.4E5) Scan definition: FTMS – c ESI d Full ms2

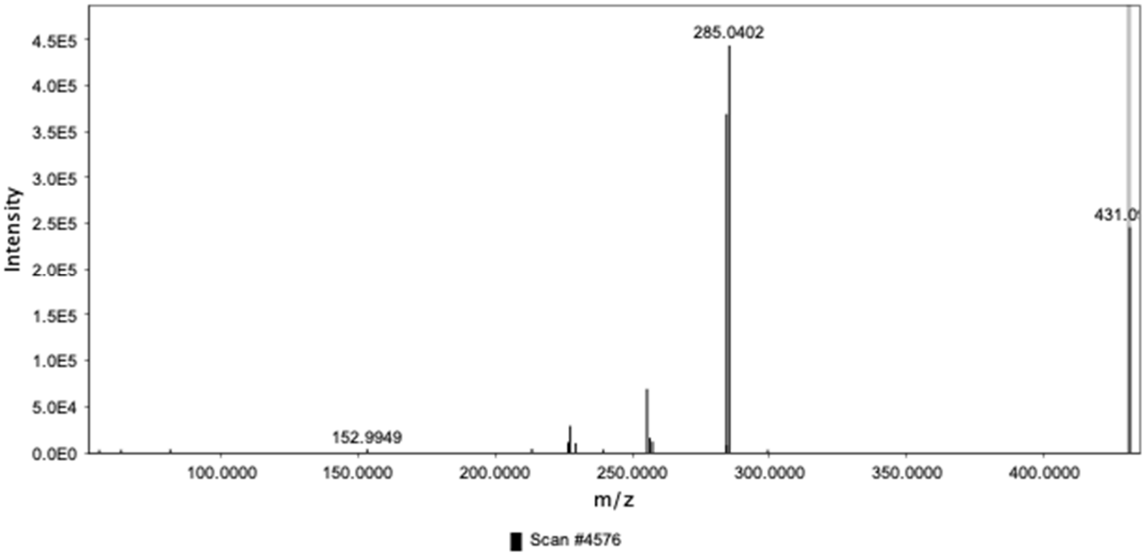

(12) Taxodium mucronatum - 5mg/mL – NEG @7.04 min MS2 - CE: 30.0 c - precursor *m/z*: 461.10827, base peak *m/z*: 461.10827 (7.6E5) Scan definition: FTMS – c ESI d Full ms2

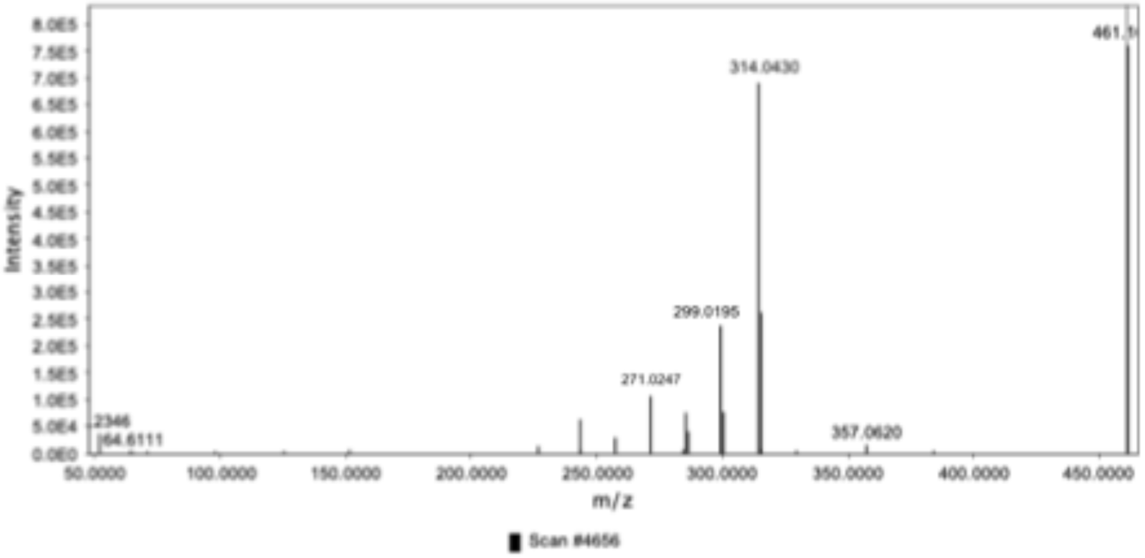

(13) Taxodium mucronatum - 5mg/mL – NEG @7.68 min MS2 - CE: 30.0 c - precursor *m/z*: 301.03523, base peak *m/z*: 301.03523 (2.2E5) Scan definition: FTMS – c ESI d Full ms2

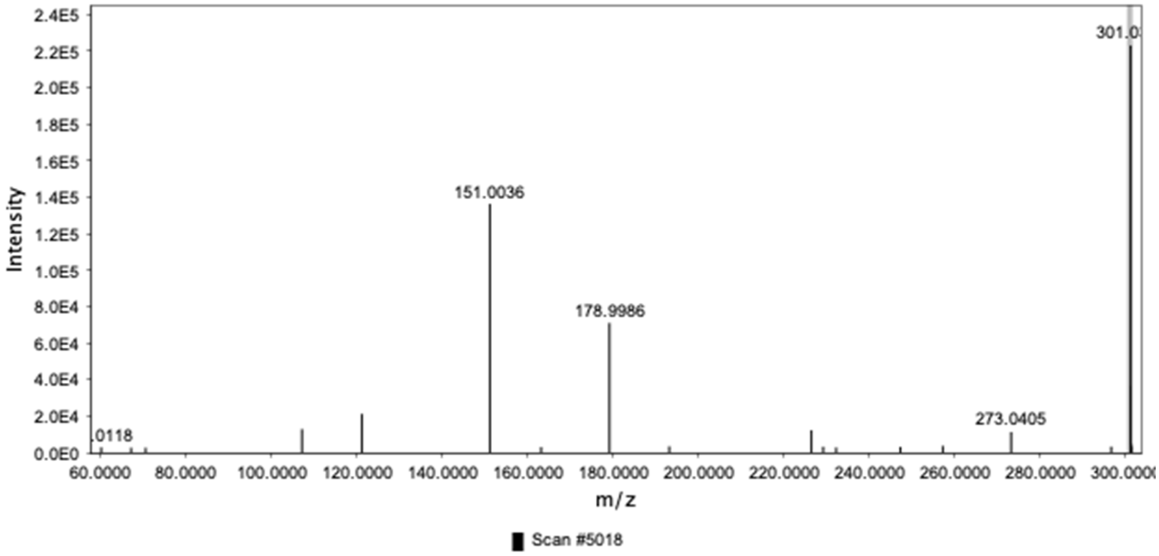

(14) Taxodium mucronatum - 5mg/mL – NEG @8.21 min MS2 - CE: 30.0 c - precursor *m/z*: 271.06105, base peak *m/z*: 271.06105 (2.7E5) Scan definition: FTMS – c ESI d Full ms2

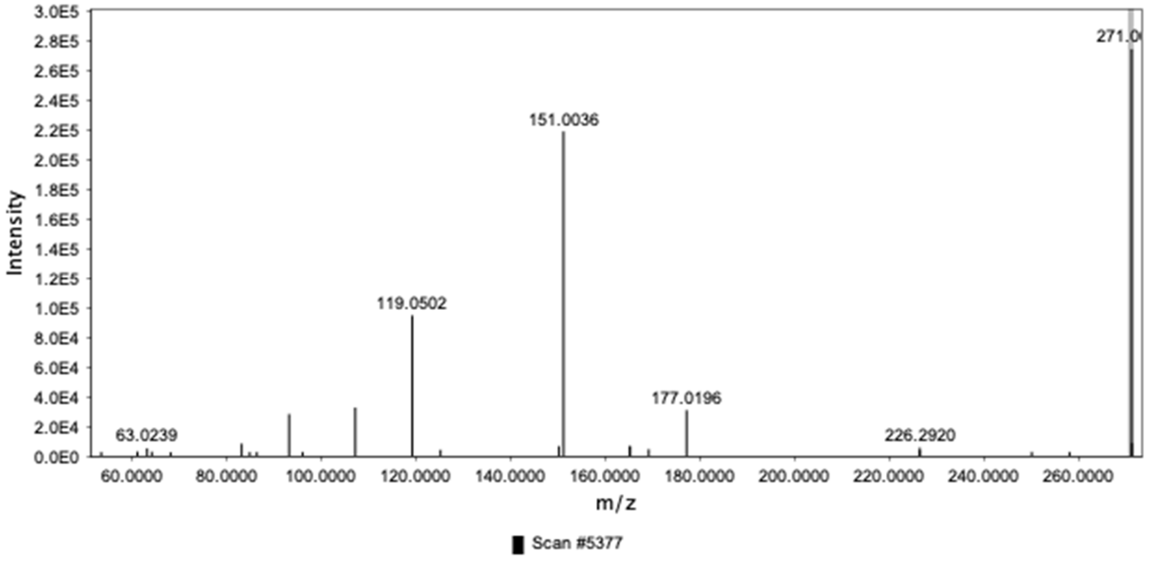

(15) Taxodium mucronatum - 5mg/mL – NEG @8.66 min MS2 - CE: 30.0 c - precursor *m/z*: 537.08206, base peak *m/z*: 537.08206 (2.7E6) Scan definition: FTMS – c ESI d Full ms2

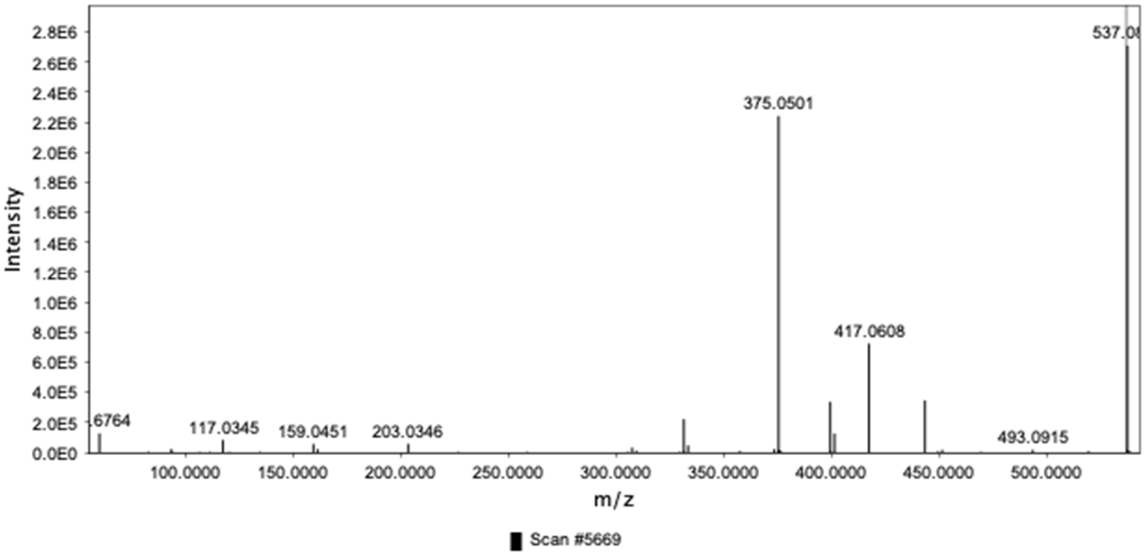

(16) Taxodium mucronatum - 5mg/mL – NEG @8.87 min MS2 - CE: 30.0 c - precursor *m/z*: 347.18564, base peak *m/z*: 347.18564 (1.3E6) Scan definition: FTMS – c ESI d Full ms2

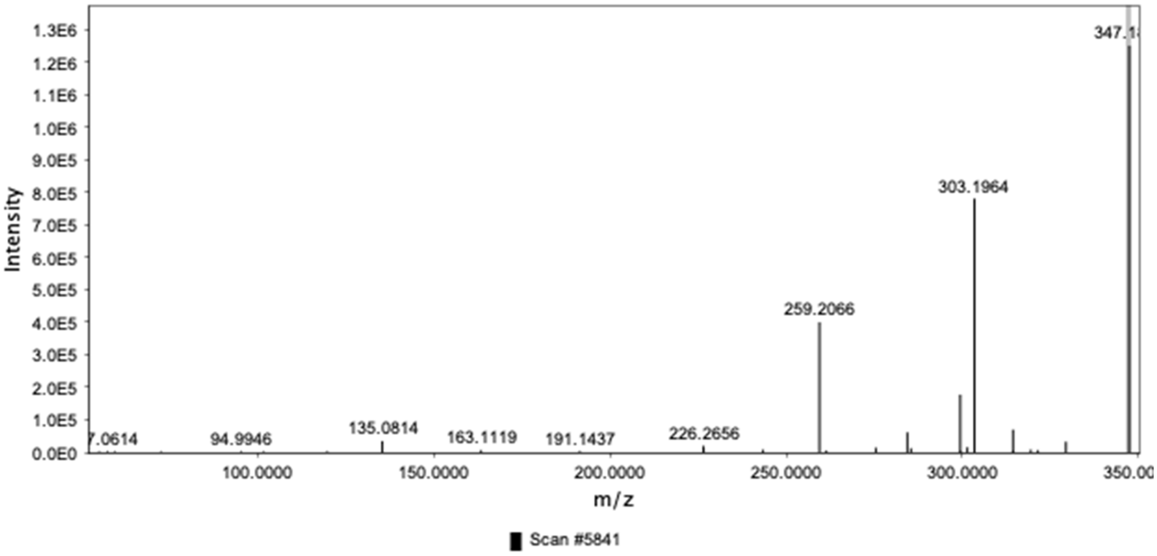

(17) Taxodium mucronatum - 5mg/mL – NEG @9.04 min MS2 - CE: 30.0 c - precursor *m/z*: 537.08179, base peak *m/z*: 537.08179 (7.4E6) Scan definition: FTMS – c ESI d Full ms2

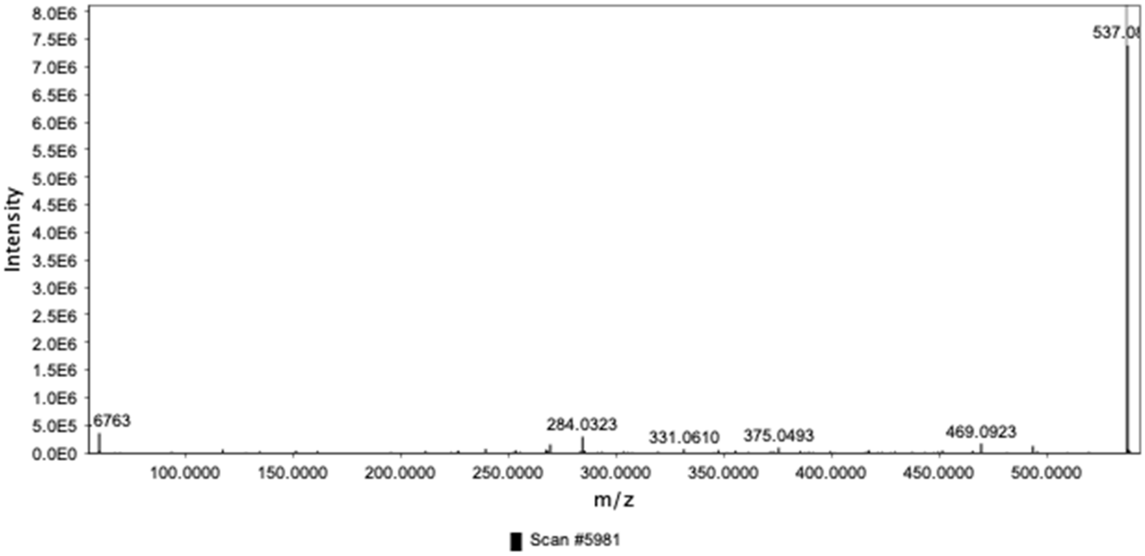

(18) Taxodium mucronatum - 5mg/mL – NEG @9.13 min MS2 - CE: 30.0 c - precursor *m/z*: 333.20662, base peak *m/z*: 315.1962 (3.8E7) Scan definition: FTMS – c ESI d Full ms2

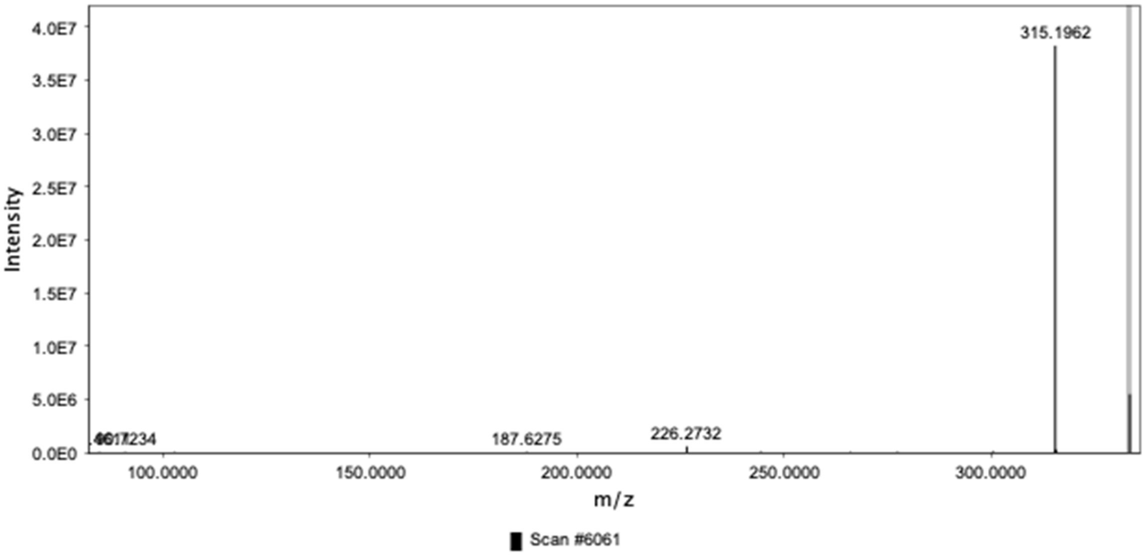

(19) Taxodium mucronatum - 5mg/mL – NEG @9.29 min MS2 - CE: 30.0 c - precursor *m/z*: 565.11334, base peak *m/z*: 533.08703 (2.9 E6) Scan definition: FTMS – c ESI d Full ms2

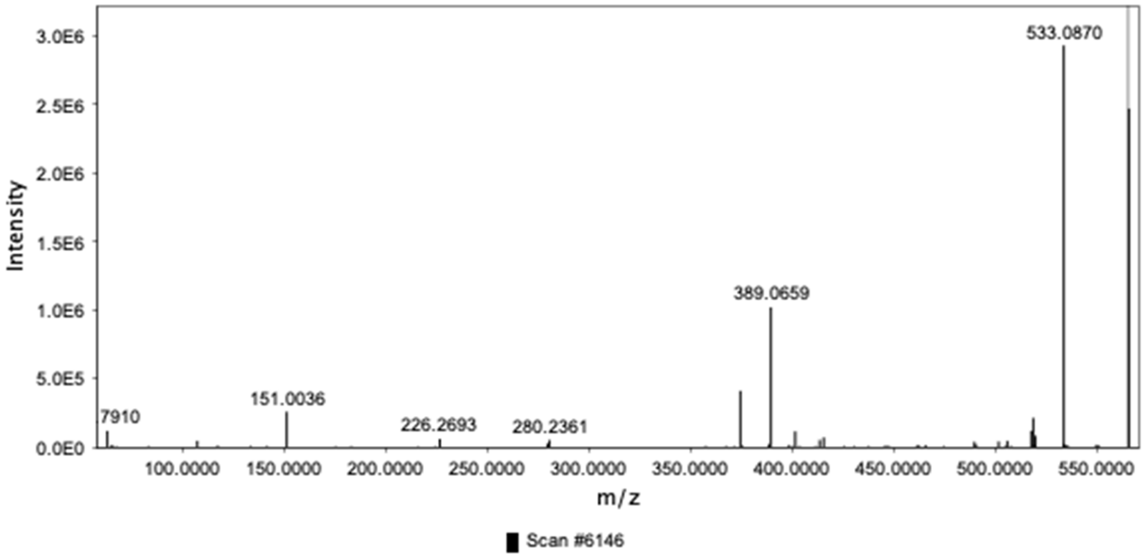

(20) Taxodium mucronatum - 5mg/mL – NEG @9.43 min MS2 - CE: 30.0 c - precursor *m/z*: 551.09777, base peak *m/z*: 551.09777 (3.5E6) Scan definition: FTMS – c ESI d Full ms2

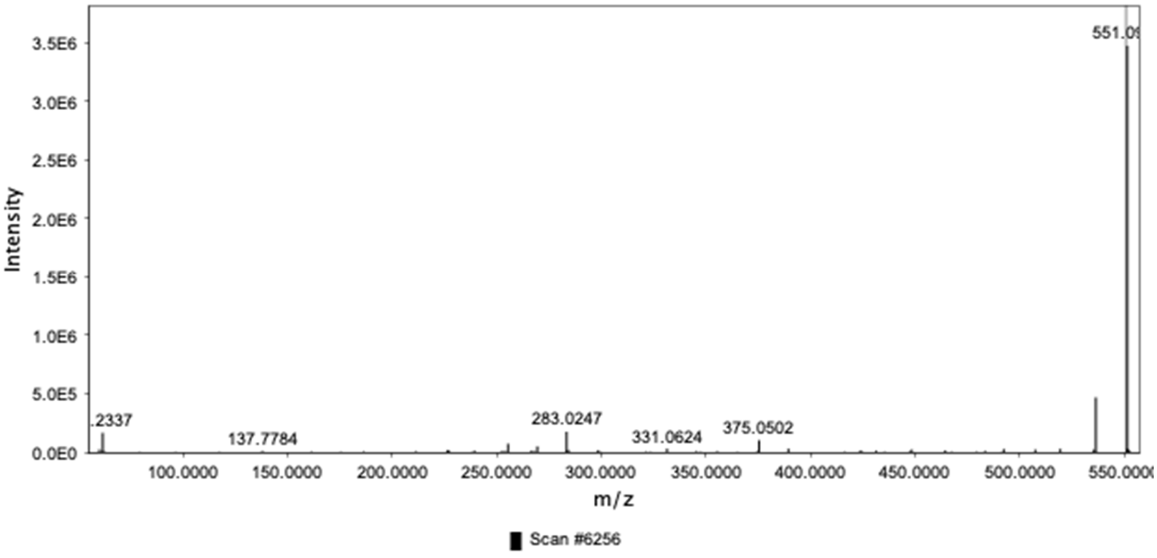

(21) Taxodium mucronatum - 5mg/mL – NEG @9.46 min MS2 - CE: 30.0 c - precursor *m/z*: 565.11336, base peak *m/z*: 565.11336 (1.0E6) Scan definition: FTMS – c ESI d Full ms2

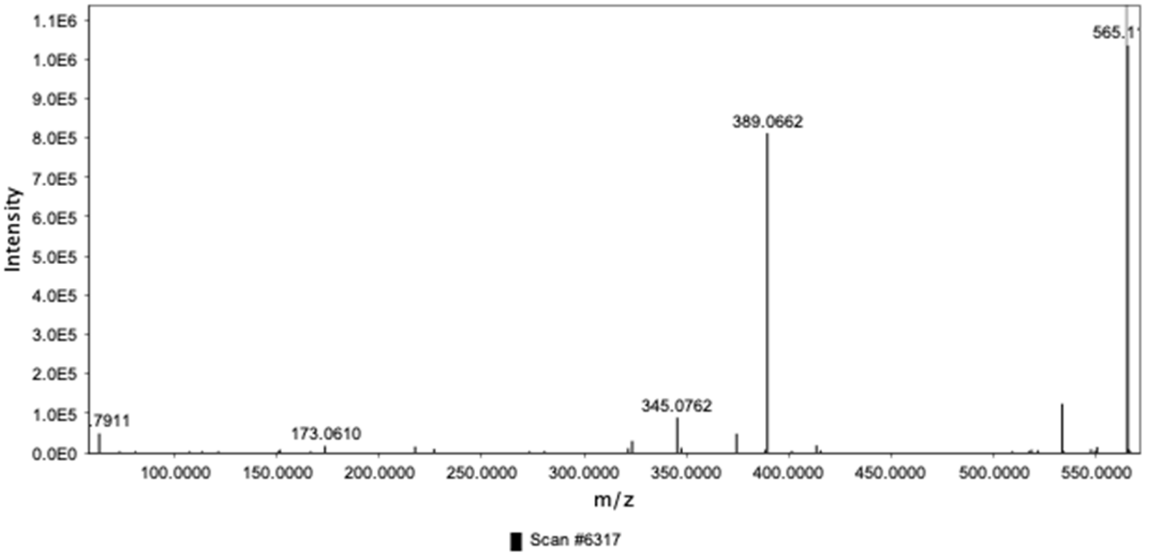

(22) Taxodium mucronatum - 5mg/mL – NEG @9.52 min MS2 - CE: 30.0 c - precursor *m/z*: 659.35833, base peak *m/z*: 329.1761 (8.0E4) Scan definition: FTMS – c ESI d Full ms2

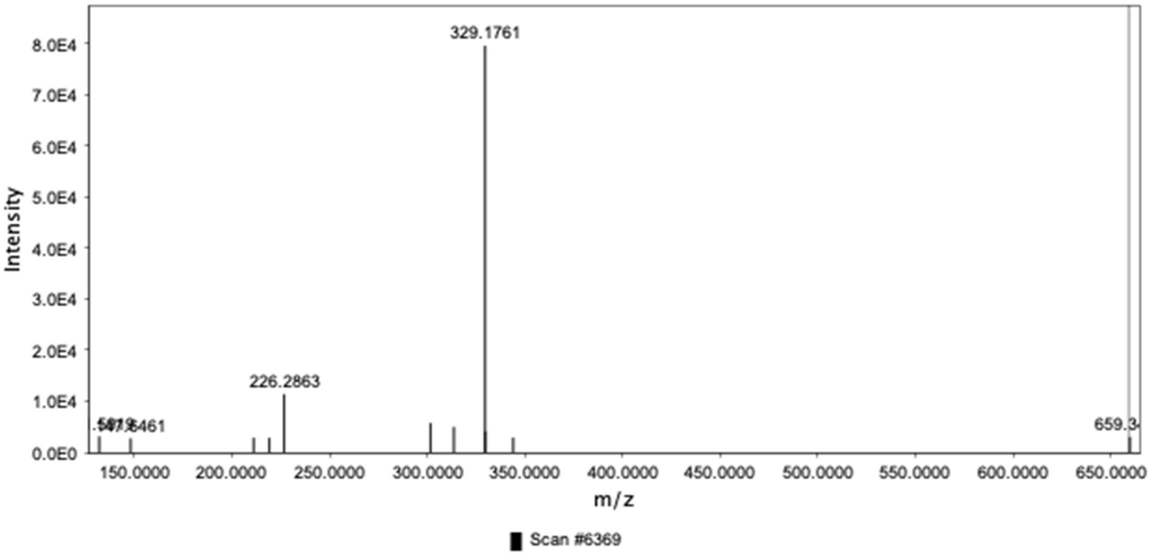

(23) Taxodium mucronatum - 5mg/mL – NEG @9.52 min MS2 - CE: 30.0 c - precursor *m/z*: 317.21185, base peak *m/z*: 317.21185 (2.5E7) Scan definition: FTMS – c ESI d Full ms2

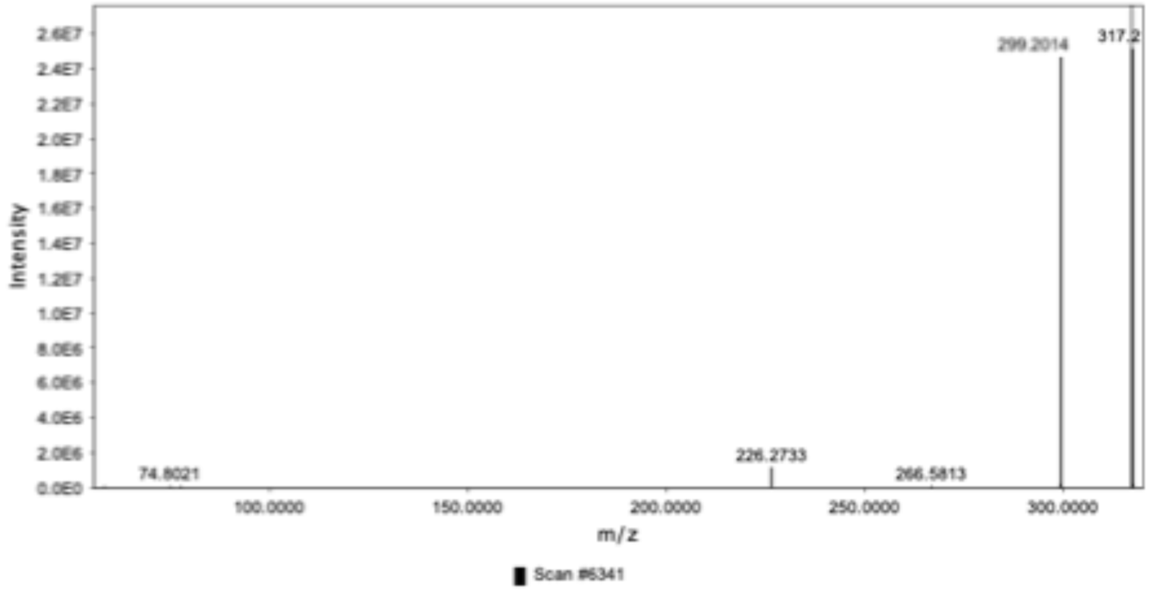

(24) Taxodium mucronatum - 5mg/mL – NEG @9.6 min MS2 - CE: 30.0 c - precursor *m/z*: 313.18056, base peak *m/z*: 313.18056 (1.1E7) Scan definition: FTMS – c ESI d Full ms2

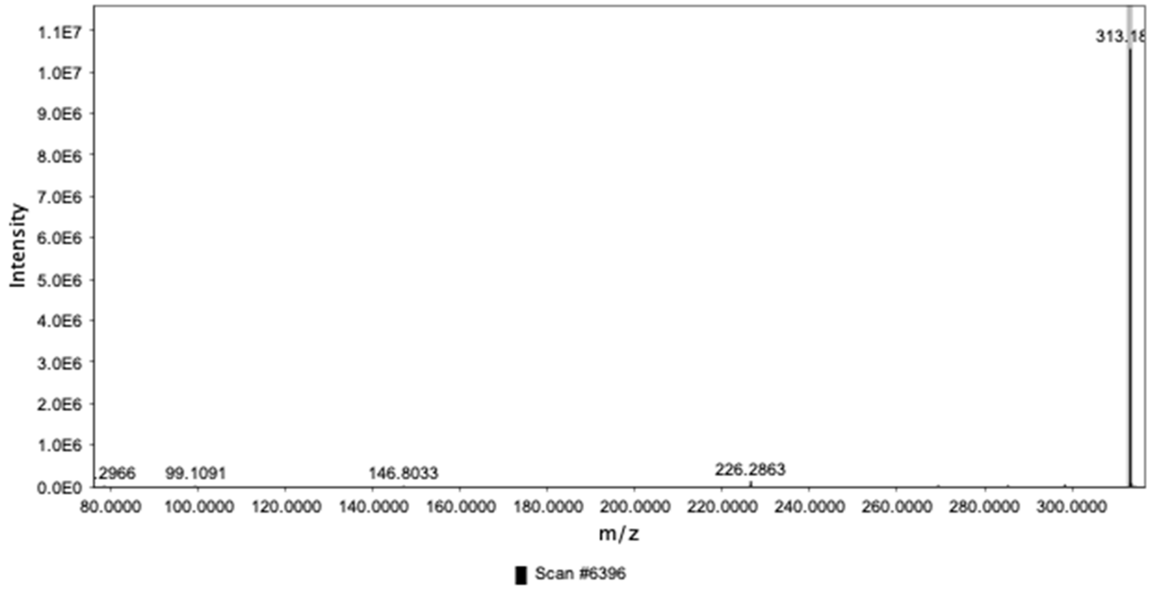

(25) Taxodium mucronatum - 5mg/mL – NEG @9.6 min MS2 - CE: 30.0 c - precursor *m/z*: 315.196, base peak *m/z*: 315.196 (2.9E6) Scan definition: FTMS – c ESI d Full ms2

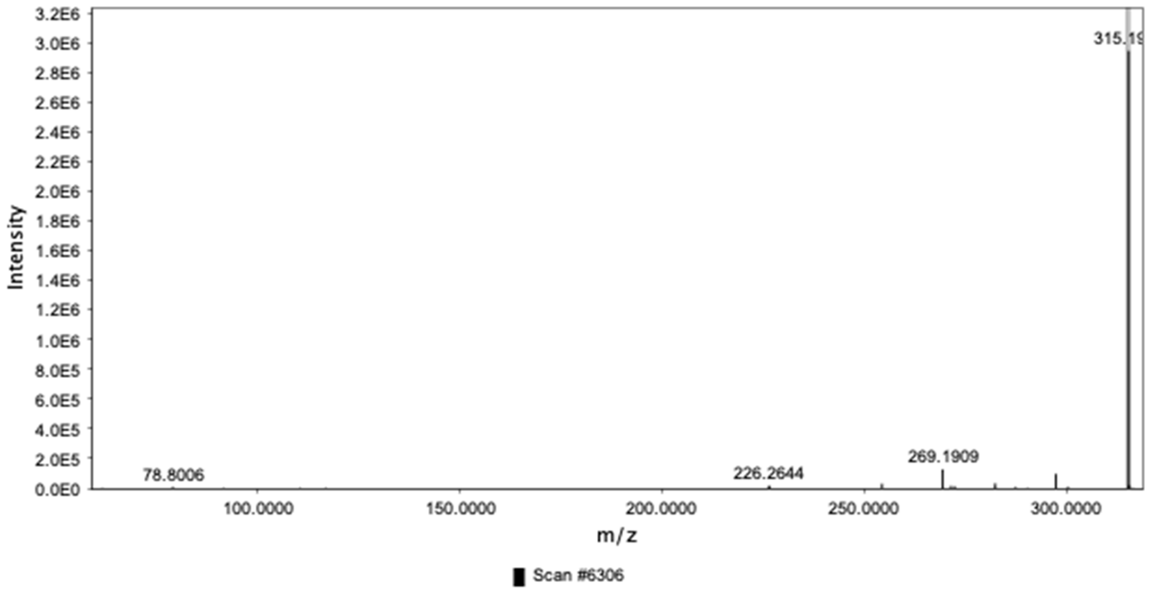

(26) Taxodium mucronatum - 5mg/mL – NEG @9.73 min MS2 - CE: 30.0 c - precursor *m/z*: 599.41004, base peak *m/z*: 299.2013 (5.6E6) Scan definition: FTMS – c ESI d Full ms2

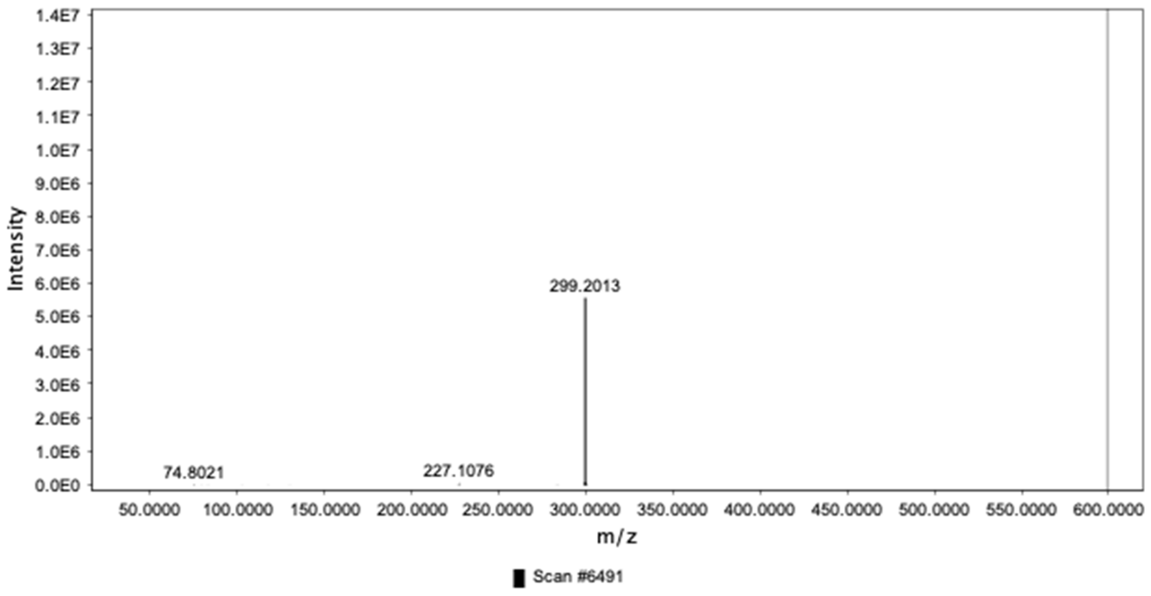

(27) Taxodium mucronatum - 5mg/mL – NEG @9.76 min MS2 - CE: 30.0 c - precursor *m/z*: 663.38969, base peak *m/z*: 331.1911 (9.4E5) Scan definition: FTMS – c ESI d Full ms2

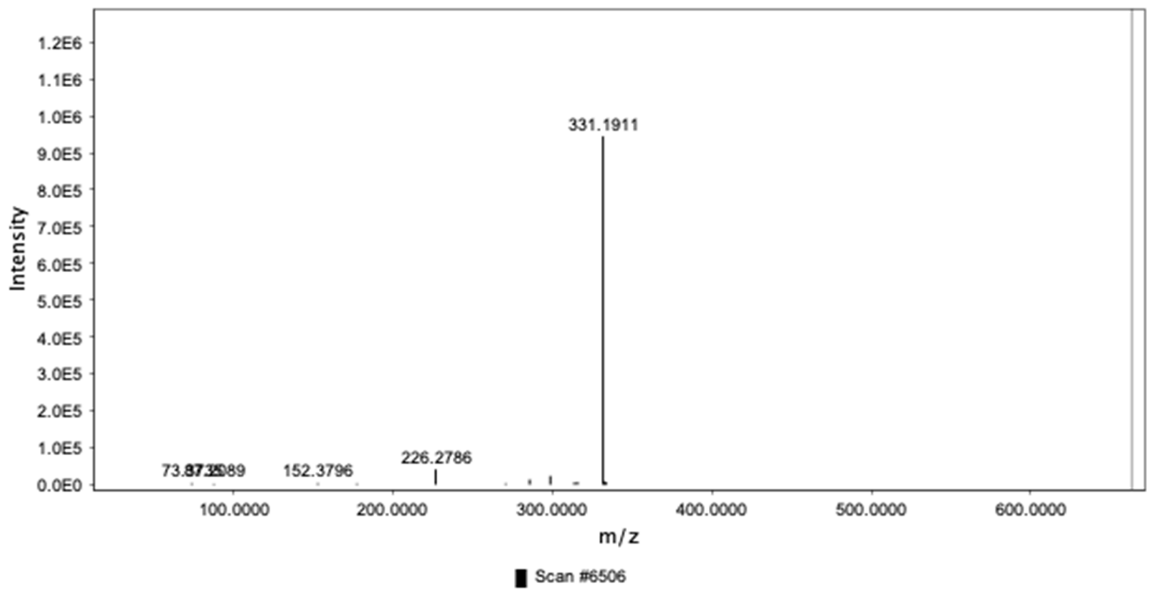

(28) Taxodium mucronatum - 5mg/mL – NEG @9.86 min MS2 - CE: 30.0 c - precursor *m/z*: 659.35809, base peak *m/z*: 329.1755 (9.4E5) Scan definition: FTMS – c ESI d Full ms2

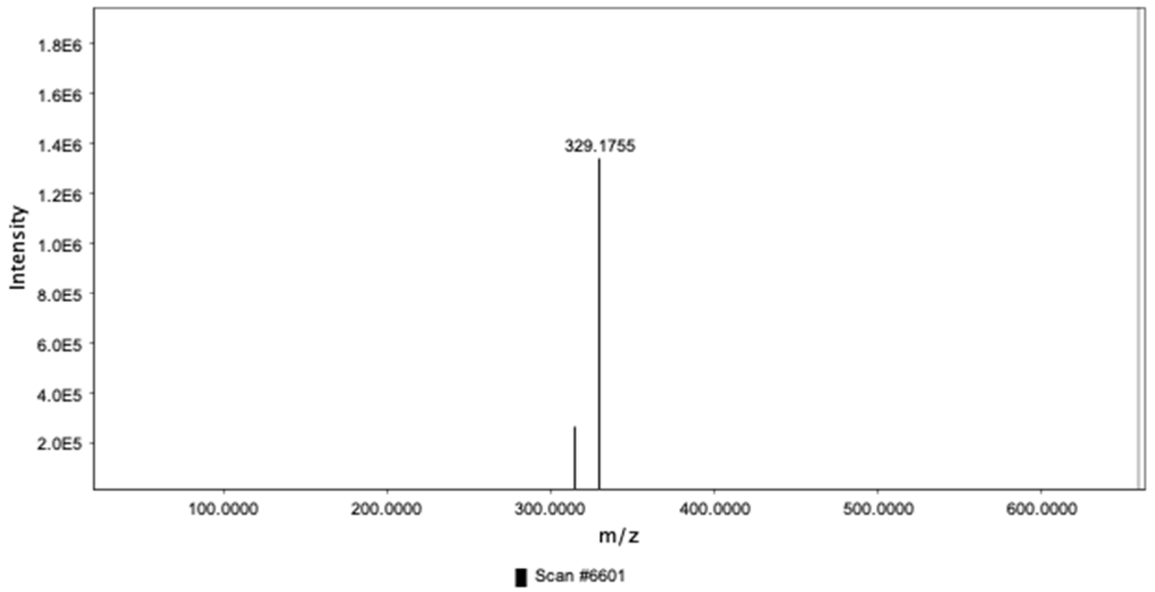

(29) Taxodium mucronatum - 5mg/mL – POS @9.96 min MS2 - CE: 30.0 c - precursor *m/z*: 315.19542, base peak *m/z*: 245.1173 (5.3E6) Scan definition: FTMS – c ESI d Full ms2

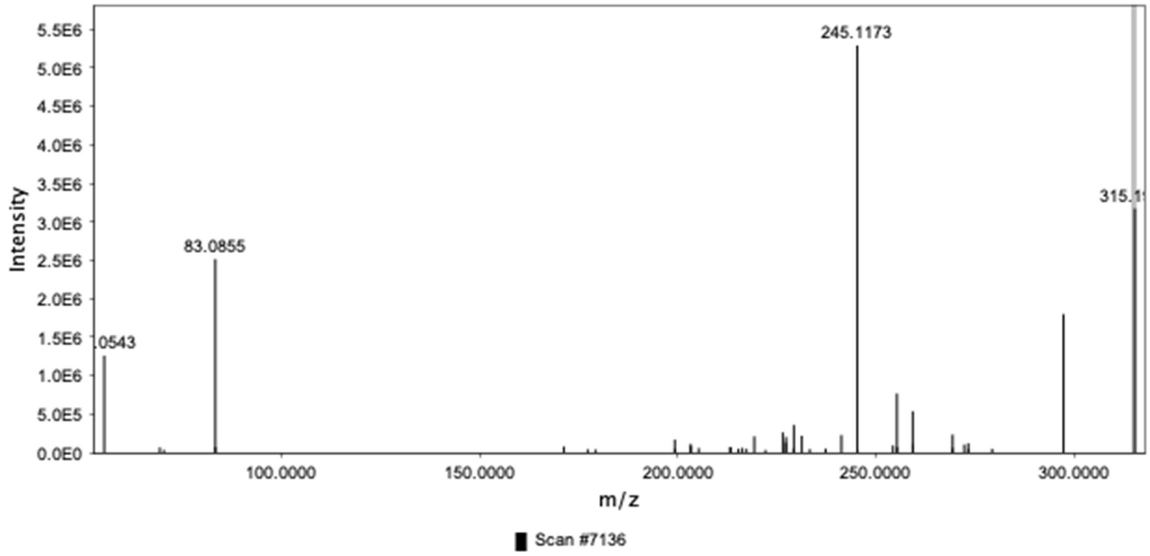

(30) Taxodium mucronatum - 5mg/mL – NEG @10.05 min MS2 - CE: 30.0 c - precursor *m/z*: 359.22188, base peak *m/z*: 298.1570 (1.0E6) Scan definition: FTMS – c ESI d Full ms2

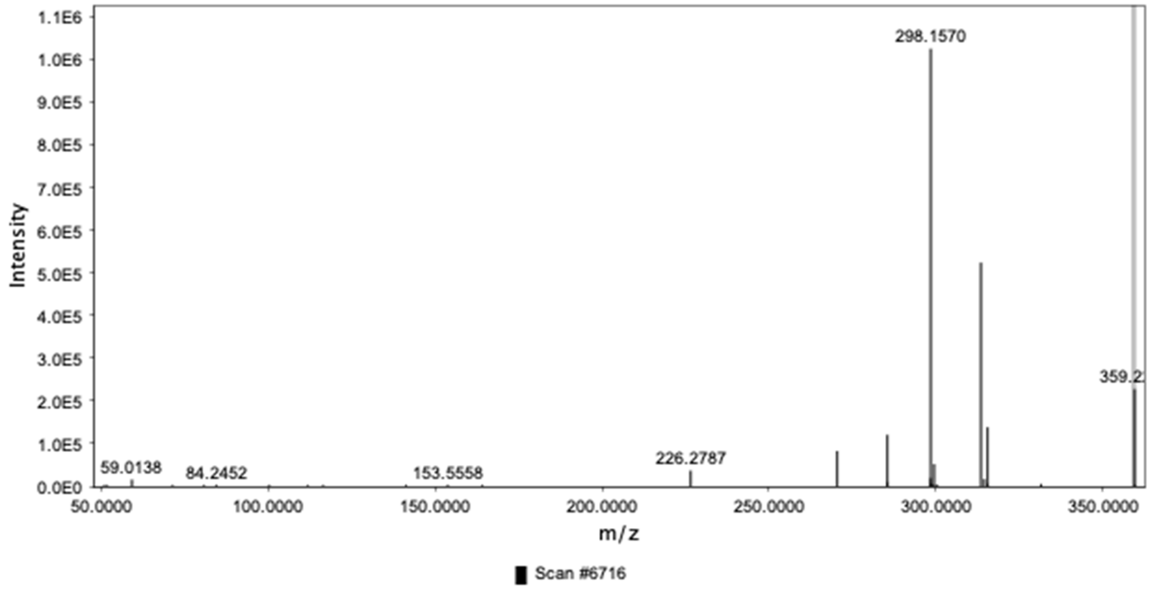

(31) Taxodium mucronatum - 5mg/mL – POS @10.15 min MS2 - CE: 30.0 c - precursor *m/z*: 333.20625, base peak *m/z*: 269.1903 (8.1E5) Scan definition: FTMS – c ESI d Full ms2

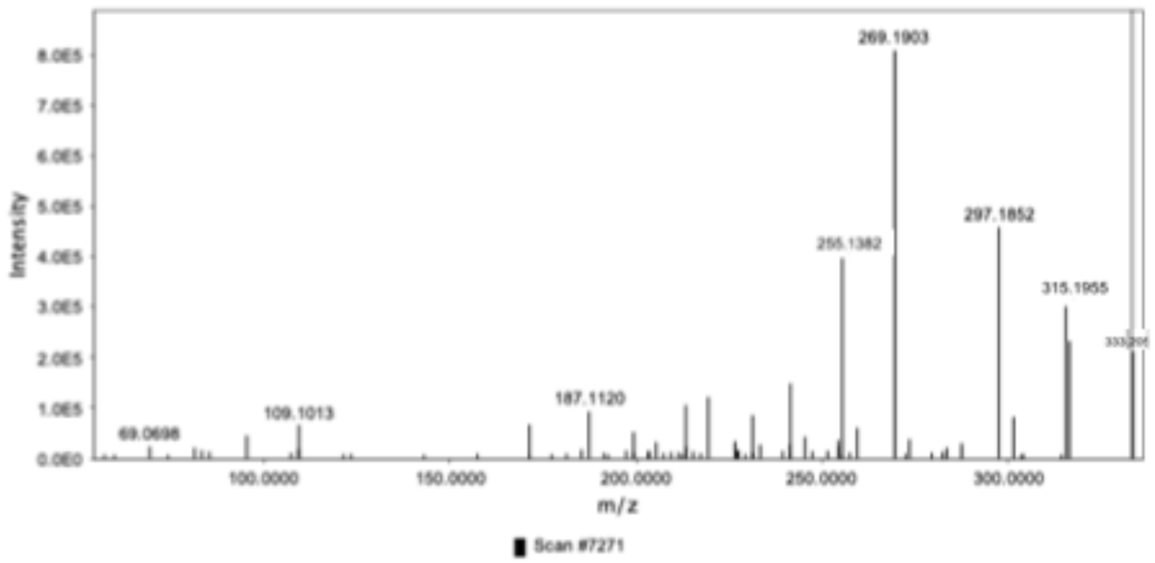

(32) Taxodium mucronatum - 5mg/mL – POS @10.49 min MS2 - CE: 30.0 c - precursor *m/z*: 301.21628, base peak *m/z*: 205.1225 (9.6E5) Scan definition: FTMS – c ESI d Full ms2

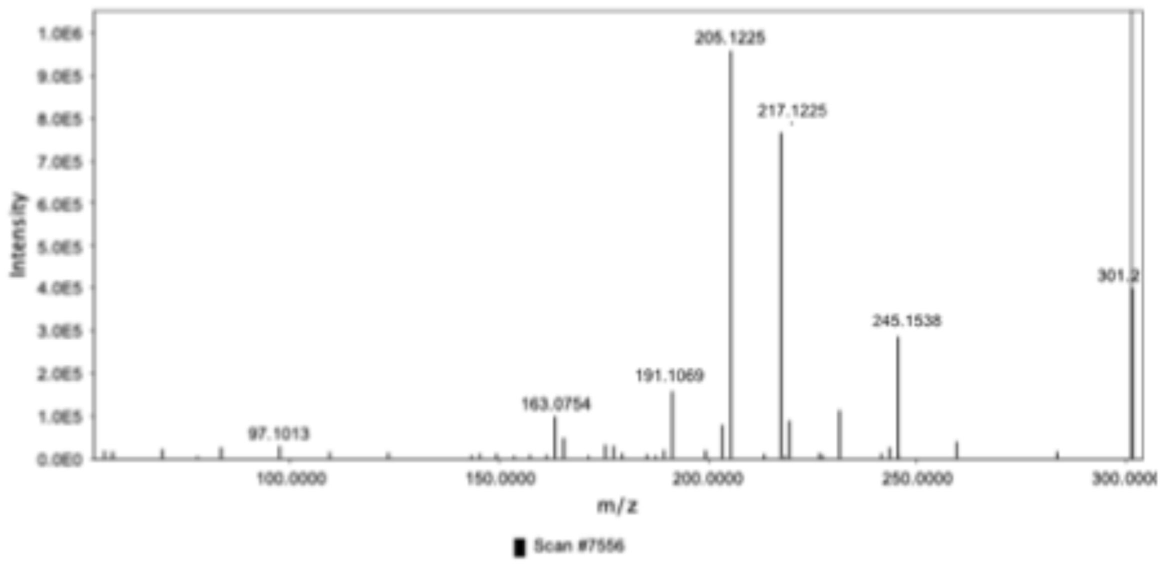

(33) Taxodium mucronatum - 5mg/mL – NEG @10.65 min MS2 - CE: 30.0 c - precursor *m/z*: 301.21703, base peak *m/z*: 301.21703 (6.9E5) Scan definition: FTMS – c ESI d Full ms2

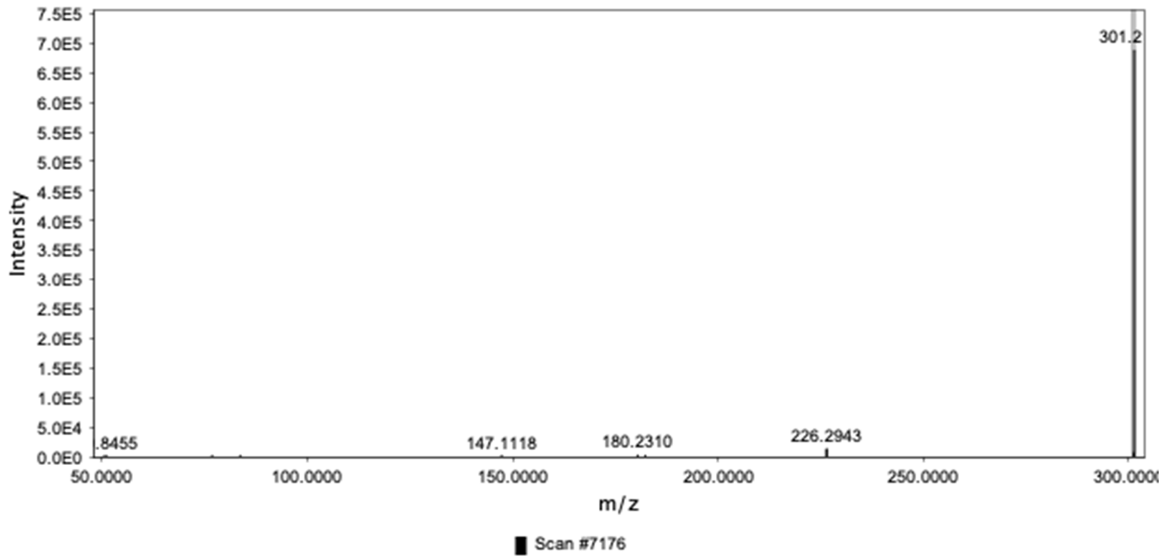

(34) Taxodium mucronatum - 5mg/mL – POS @10.79 min MS2 - CE: 30.0 c - precursor *m/z*: 301.21633, base peak *m/z*: 205.1225 (6.2E5) Scan definition: FTMS – c ESI d Full ms2

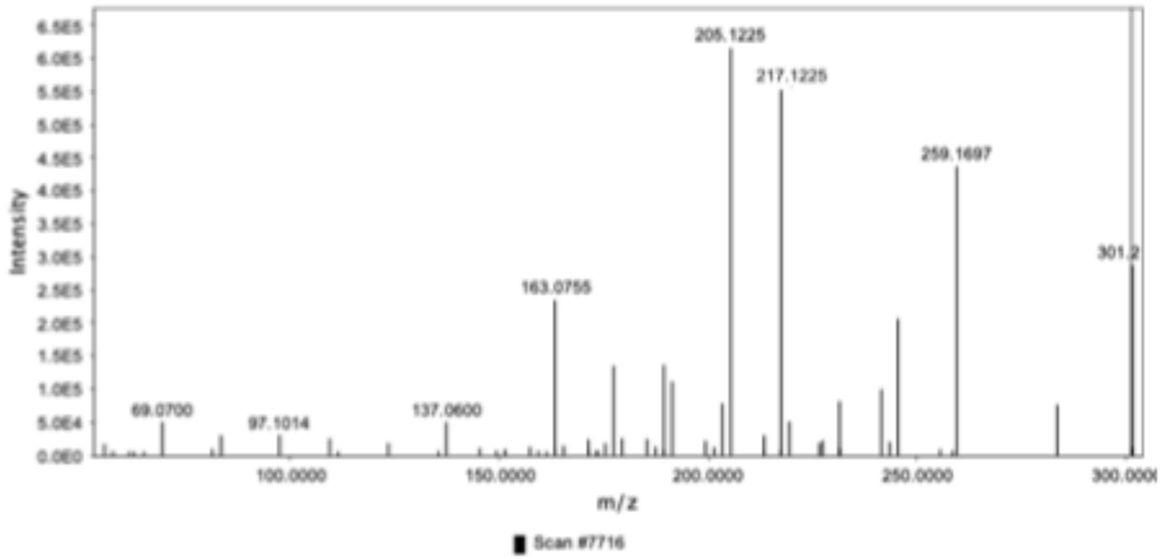

(35) Taxodium mucronatum - 5mg/mL – POS @11.18 min MS2 - CE: 30.0 c - precursor *m/z*: 301.21607, base peak *m/z*: 205.1224 (1.2E7) Scan definition: FTMS – c ESI d Full ms2

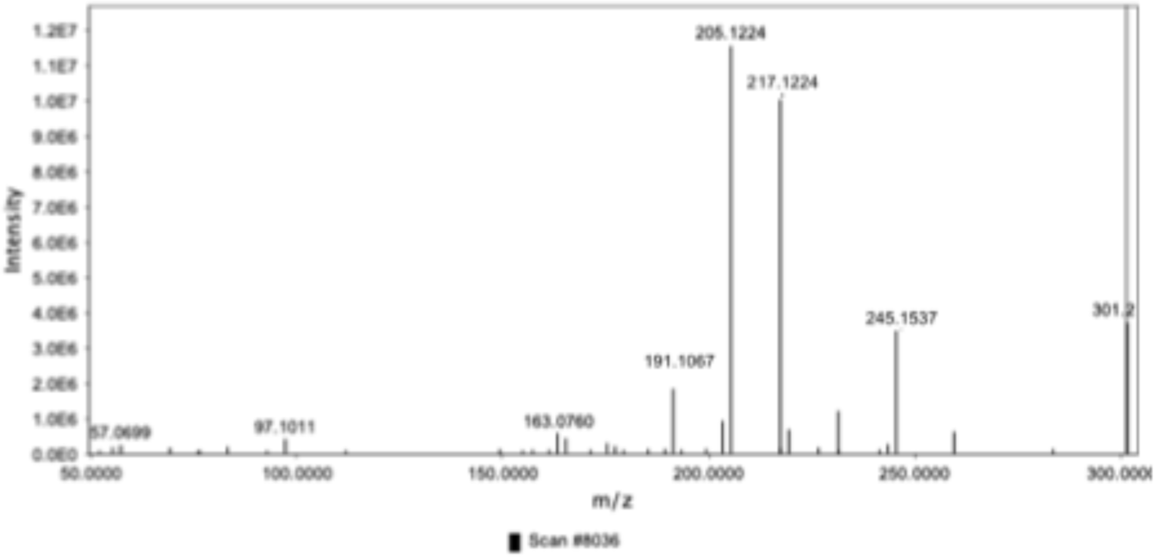

Supplement: Supplementary file 1 [file plants-15-03391-s001.zip › SMS1 - Annotated Mass Spectra_rev1.pdf]
