# Supplementary material for: Phenolic Profile, Antioxidant Activity, and Wound-Healing Potential of Oenothera rosea and Taxodium mucronatum Extracts
Source: Plants (Basel). 2026 Nov 2;15(21):3391. doi: 10.3390/plants15213391 (PMC13765647; doi:10.3390/plants15213391)
Supplement: Supplementary file 1 [file plants-15-03391-s001.zip › SMS2 - Plant Material_rev1.pdf]

Supplementary Material S2:
Plant Material Used in the Study

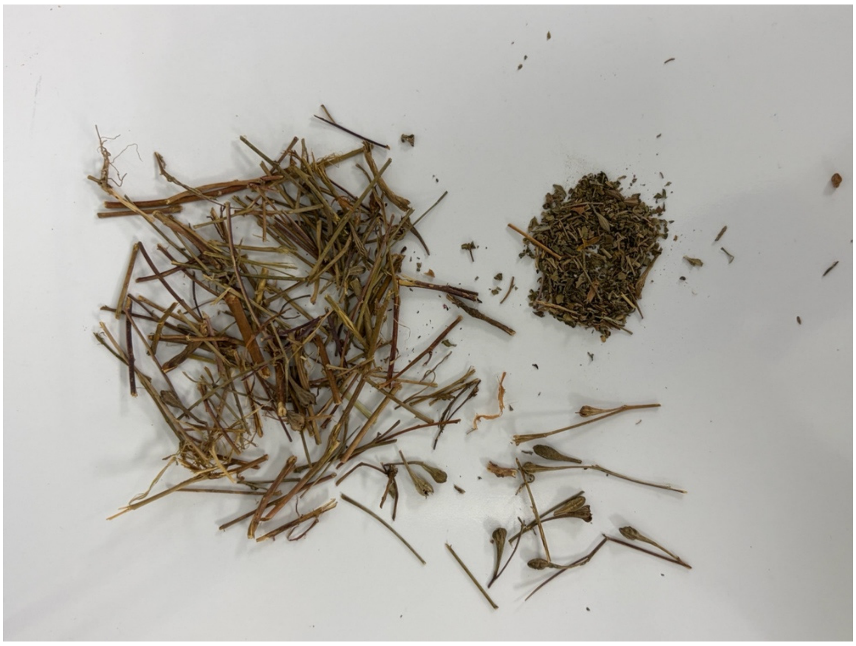

Figure S3. Representative photograph of commercially obtained Oenothera rosea ("hierba del golpe") prior to processing.

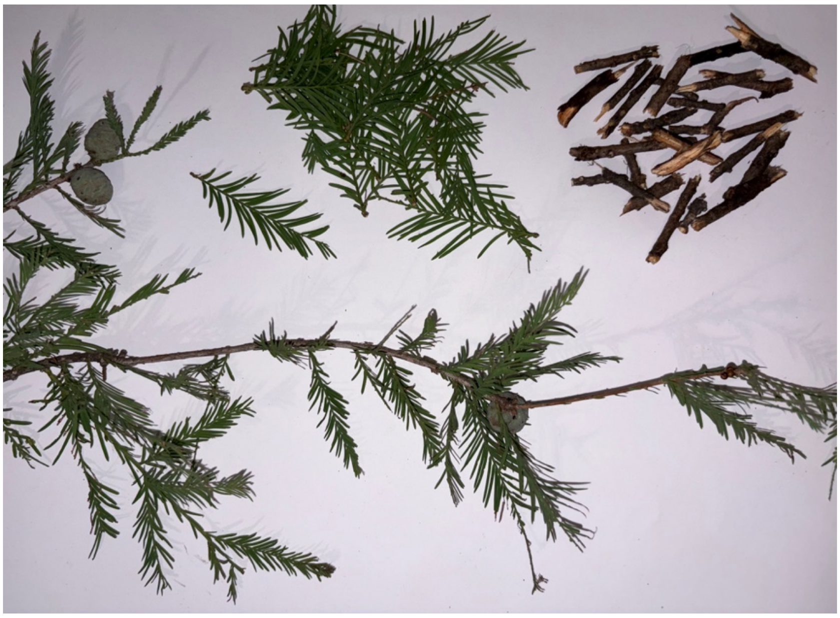

Figure S4. Representative photograph of Taxodium mucronatum collected near Monterrey, Nuevo León, México, showing the aerial tissues used for sample preparation. Plant identification was performed using standard taxonomic keys and floristic references.

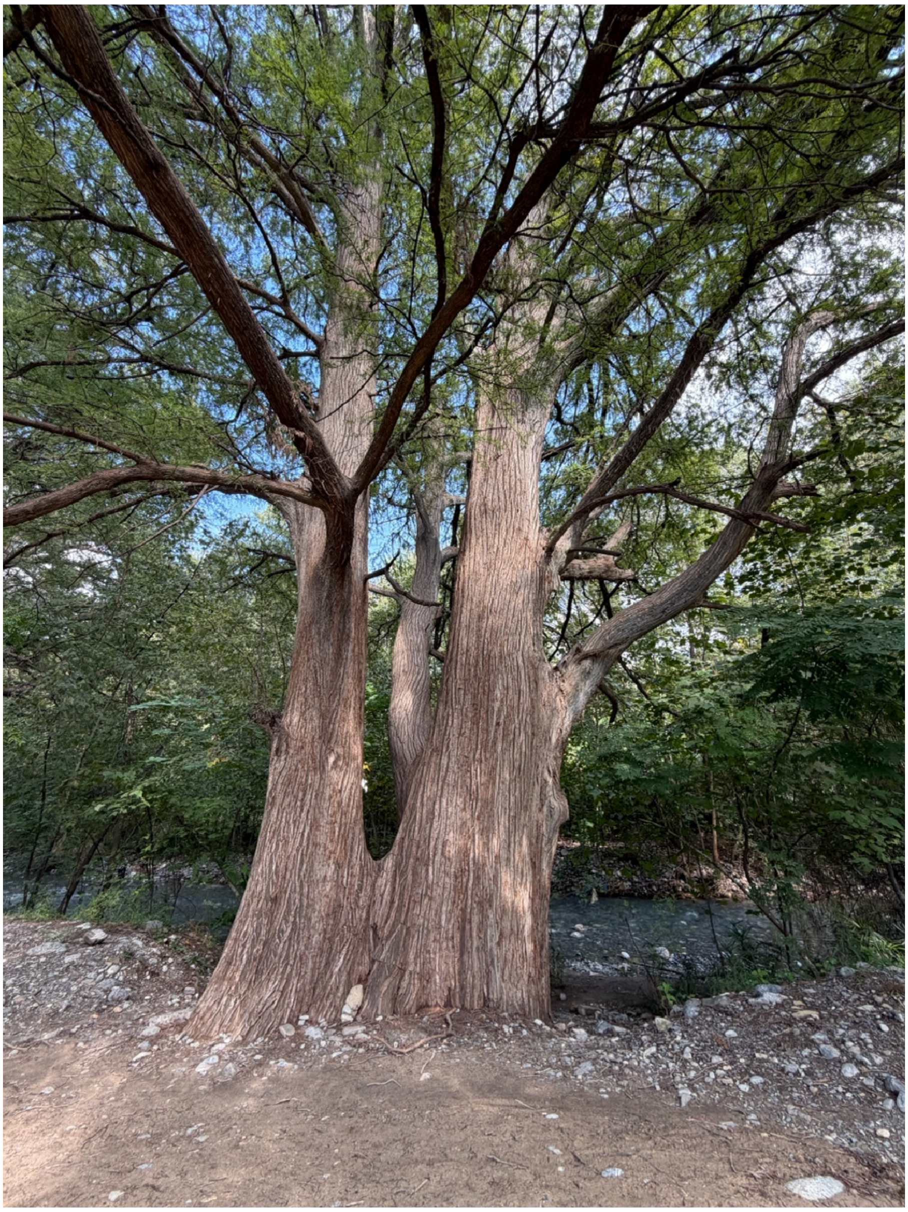

Figure S5. Taxodium mucronatum tree at the collection site in Monterrey, Nuevo León, Mexico.
